# EndoNUclease Heteroduplex cleavage typing a new technique for rapid typing of bacterial isolates in the context of nosocomial outbreaks: proof of concept with *Bacillus cereus*

**DOI:** 10.3389/fcimb.2026.1813523

**Published:** 2026-06-09

**Authors:** Elissa Maalouf, Eva Lopez, Bruno Coutard, Remi N. Charrel

**Affiliations:** 1 Unite des Virus Emergents (UVE: Aix-Marseille Univ, Universita di Corsica, IRD 190, Inserm 1207, IRBA), Marseille, France; 2 Le Service de Prévention du Risque Infectieux (LeSPRI), CLIN AP-HM Hôpitaux Universitaires de Marseille, Marseille, France; 3 Laboratoire des Infections Virales Aigues et Tropicales, AP-HM Hôpitaux Universitaires de Marseille, Marseille, France

**Keywords:** *Bacillus cereus*, ENUHCT, hospital-acquired infections (HAIs), molecular typing, outbreak investigation and control

## Abstract

**Introduction:**

*Bacillus cereus* is a Gram-positive, spore-forming bacterium implicated in foodborne illnesses and severe nosocomial infections, particularly in immunocompromised patients. In France, *B. cereus* has been associated with neonatal infections, including septicemia and central nervous system infections, with a significant mortality rate. The bacterium’s spores are highly resistant to disinfectants, contributing to hospital-acquired infections through environmental contamination. Current typing methods, such as Multi-Locus Sequence Typing (MLST) are time-consuming and not routinely performed in clinical settings, limiting real-time outbreak management.

**Methods:**

This study evaluates the EndoNUclease Heteroduplex Cleavage Typing (ENUHCT) as a rapid alternative to MLST for *B. cereus* strain typing. ENUHCT identifies single nucleotide polymorphisms by cleaving mismatched DNA heteroduplexes, enabling rapid discrimination between strains. 14 *B. cereus* strains (clinical and environmental) from two hospitals were analyzed using both MLST and ENUHCT. ENUHCT results were congruent with MLST, successfully distinguishing identical and closely related strains. ENUHCT provided results within hours, compared to days for MLST, and required no prior sequencing.

**Results:**

ENUHCT demonstrated high discriminatory power, with strains sharing identical STs showing no cleavage, while strains with differing STs exhibited distinct cleavage patterns. Phylogenetic analysis based on ENUHCT data closely matched MLST-derived trees, highlighting its potential for real-time outbreak tracking. However, ENUHCT’s accuracy decreased with high Single Nucleotide Polymorphisms (SNP) counts (≥4), as peak detection became less reliable. Despite this limitation, ENUHCT proved effective for initial strain screening, reducing the need for full genome sequencing in unrelated strains.

**Conclusion:**

In conclusion, ENUHCT offers a rapid, cost-effective alternative to MLST for *B. cereus* strain typing, particularly in outbreak settings. Its ability to provide real-time results supports timely infection control measures, though further validation is needed for broader application. ENUHCT’s adaptability to other pathogens with established MLST schemes underscores its potential as a first line typing tool in clinical microbiology.

## Introduction

Bacillus cereus is a spore forming Gram positive bacterium, ubiquitously present in the environment (food, soil, air, surfaces). In France, *B. cereus* is the second most frequent agent in bacterial food-borne epidemics after *Staphylococcus aureus* (Glasset et al., 2016). Diarrheal food poisoning is caused by *B. cereus* enterotoxins, and vomiting food poisoning is caused by emetic toxins. *B. cereus* is also implicated in cases of severe systemic bacteremia in immunocompromised patients including premature infants, causing death in 10% of neonatal cases (Lotte et al., 2017). In France, the public health agency reported 49 cases of neonatal B. cereus infection and 16 deaths in preterm infants from 2002 until 2016 (Fournier et al., 2018), *B. cereus* is responsible for intra-hospital systemic and local infections as well as cross-contaminations between hospitals. In neonates, *B. cereus* cases present as septicemia, endocarditis, pneumopathy and central nervous system infections. *B. cereus* spores that can persist in the environment (Kotiranta et al., 2000) and can resist to chemical disinfectants (Glasset et al., 2018; Lotte et al., 2022) are often involved in hospital associated infections (HAI). Contaminations from the environment often occur from hospital linen (Lee et al., 2021), ventilation and filtration systems, medical devices, hands of healthcare workers (Sasahara et al., 2011; Tsai et al., 2021). Contaminations of gloves, and hydro alcoholic gel have been documented (Lotte et al., 2022). It can also be found in contaminated breast milk as well as baby formula, where spores can resist to pasteurization (Zhuang et al., 2019). Epidemiological and molecular typing of clinical isolates have revealed close genetic relatedness with environmental samples (Okutani et al., 2019). On the other hand, failure to identify the source can be explained by the retrospective nature of environmental sampling done often too late after an outbreak (Lotte et al., 2022).

In hospital settings, current methods for typing *B. cereus* isolates consist in both sequence-based and molecular typing not based on sequencing. Sequence-based typing methods previously used in hospital diagnostics for *B. cereus* are 16S RNA sequencing, panC sequencing and Multi-Locus Sequence Typing (MLST). MLST and core-genome sequencing cg-MLST are some of the most discriminative methods for bacterial strain typing (Li et al., 2009). Several studies used molecular typing not based on sequencing such as pulsed-field gel electrophoresis to establish the genetic relatedness between clinical and environmental strains (Akamatsu et al., 2019).

MLST characterizes bacterial isolates through sequencing 450–500 bp regions of housekeeping genes (Maiden et al., 2013), ultimately generating an allelic profile also called sequence type (ST) (Jolley and Maiden, 2017). Each strain is characterized by a unique ST; STs can be compared between clinical strains, between environmental strains, between clinical and environmental strains to help understanding the mechanisms of transmission. ST can also be compared between laboratories and to those of the large central database (PubMLST.org). MLST can be directly applied on clinical samples without a prior isolation phase (Chiani et al., 2016).

MLST, along with others sequence-based methods, is limited by time consumption when performed manually. It can also be limited by the number of samples to study. Data analysis requires experience and knowhow in multiple areas such as DNA sequence analysis and sequence type assignment (Maiden et al., 2013). The turnaround time of methods like MLST can be up to 1 week including sample processing, sequencing and data analysis. Output of the sequences should be compared to experimental data for correctness and quality control. A recent study combined MLST of five genes of *B. cereus* (glpF, ilvD, gmk, pur, pta) together with rpoB to increase discrimination of within species that cannot be identified using 16rRNA typing (Hakovirta et al., 2016). Practically, B cereus MLST analysis is not routinely performed in microbiology or hygiene laboratories but rather done by the National Reference Center where strains are transmitted for analysis when an outbreak occurs. As a consequence, the results are not used for the “real-time” elucidation of transmission dynamics and cannot guide the curative and preventive measures implemented in the context of grouped cases of epidemics (Glasset et al., 2018).

To avoid sequencing-dependent technologies and shorten the time between the sampling and release of the results, the objective of the study was to investigate the use of a genotyping technology based on mismatch identification in PCR products from samples and reference material. We selected *B. cereus* as the focus of this study for several reasons. First, as mentioned before, it has emerged as an important nosocomial pathogen, particularly in neonatal and immuno-compromised patients. Second, rapid and accurate strain typing is critical for infection control in hospital settings. Finally, at the time of this study, our hospital was conducting another ongoing investigation of *B. cereus* cases, providing a timely opportunity to evaluate typing methods in a real-world context.

The technology has already been applied for the identification of mutations in *brca1* and *brca2* genes in the case of hereditary breast cancer (Pilato et al., 2012), and more recently to detect mutations within the coding sequence of the Receptor Binging Domain (RBD) of SARS-CoV-2 within hours after the swab sampling (Lopez et al., 2021) Here we present the EndoNUclease Heteroduplex Cleavage Typing (ENUHCT) applied to the multi-loci scheme as described in PubMLST.org for the rapid identification of clonal and non-clonal *B. cereus* strains.

## Materials and methods

### Selected bacterial strains

A total of 14 *B. cereus* isolates were included in this study, comprising strains from the neonatology department of Hospital 1 (H1) in Marseille as well as unrelated strains from other hospital sites (**Table 1**). Seven isolates were obtained from clinical samples, and seven were derived from environmental specimens. Among these, 12 isolates originated from Hospital 1 (H1). These H1 isolates had been previously characterized and grouped into clusters, defined as two or more isolates sharing identical sequence types (STs) based on MLST. Two additional isolates were obtained from Hospital 2 (H2), located approximately 30 km from H1.

**Table 1:** Description of isolates used in this study.

Strain	Hospital	Outbreak	Sampling date	Source	Type	ST
BCER1	1	Yes	October 2021	Clinical	Blood culture	ST127
BCER2	1	No	August 2020	Clinical	Blood culture	ST1120
BCER3	1	Yes	September 2020	Clinical	Blood culture	ST24
BCER4	1	Yes	November 2021	Clinical	Blood culture	ST795
BCER5	2	No	April 2022	Clinical	Blood culture	ST26
BCER6	1	Yes	March 2022	Environmental	Incubator (Thermal seal)	ST999
BCER7	1	Yes	October 2019	Environmental	Water analysis	ST8
BCER8	1	Yes	October 2019	Environmental	Water analysis	ST127
BCER9	1	Yes	October 2019	Environmental	Water analysis	ST73
BCER10	1	Yes	October 2019	Environmental	Water analysis	ST1366
BCER11	1	Yes	October 2019	Environmental	Water analysis	ST18
BCER12	2	No	April 2022	Clinical	Blood culture	ST144
BCER13	1	Yes	August 2021	Clinical	Blood culture	ST26
BCER14	1	Yes	October 2019	Environmental	Storage room trolley	ST257

### MLST PCR, sequencing and ST

One colony of each of the 14 clinical and environmental samples of *B. cereus* were suspended in 200µL of general lysis buffer G2 (Qiagen REF1014636). The strains were incubated for 10 min at 100 °C. DNA was then purified using the DNA Tissue kit (Qiagen) onto the EZ2 Biorobot (Qiagen) and eluted into a 200µL volume of elution buffer TE. PCR products corresponding to *glpF, gmk, ilvD, pta, pur, pycA and tpi* were amplified as described in the MLST Database (*http://www.pubmlst.org/bcereus*) using standard primers, and *ilvD_2b* primer option replacing *ilvD*, for toxin emetic strains (**Table 2**). After performing conventional MLST, The Hunter-Gaston discriminatory index (D) was calculated for the MLST scheme to assess its ability to distinguish between *B. cereus* strains. D is defined with *N* as the total number of unrelated strains, *S* as the number of distinct types, and *x j* ​as the number of strains of type *j*. A D value of 1 indicates complete discrimination of all strains.

**Table 2:** Primer sequences, size of sequences and PCR products.

Gene*	Primer Sequence	Analyzed sequence (bp)	PCR product (bp)
*glpF*	F-GCGTTTGTGCTGGTGTAAGT	372	549
	R-CTTCTTCCTTCCGATTGCAG		
*gmk*	F-GCAATGTTCGCCAACCACAA	504	600
	R-CCTACCCTTCCTCACTTAACT		
IlvD*	F-CGGGGCAGACGTTAAGAGAGAA	393	556
	R-GAATGGAAACGACCAGAACC		
*pta*	F-TGCAATGCGAGTTGCTTCTA	414	576
	R-TTCTTTTGCTAAACGCTCTGC		
*Pur*	F-CTGCTGCGAAAAATCACAAA	348	536
	R-TTATTGCAGCGAATCGTGAG		
*pycA*	F-CGCGTCCAAGTTTATGGAAT	363	550
	R-CTTTCGTTTCCACCTAACGC		
*Tpi*	F-CCGAAACCGTCAAGAATGAT	435	558
	R-GTCGCTAAGTGCTACTGGGC		
*IlvD_2b*	F-AGATCGTATTACTGCTACGG	393	586
	R-GTTACCATTTGTGCATAACGC		

* glpF, glycerol uptake facilitator protein; gmk, guanylate kinase; ilvD, dihydroxy-acid dehydratase; pta, phosphate acetyltransferase; pur, Phosphoribosyl-amin o-imidazole carboxamide; pycA, pyruvate carboxylase; tpi, triosephosphate isomerase.

Once PCR products in the selected loci are obtained, they are mixed with one another and incubated with a mismatch-specific enzyme that detects and cleaves mismatches in the double stranded DNA, that are synonymous with SNPs. PCR reaction consisted of 12.5µL AmpliTaq Gold 360 Master Mix (Thermo Fisher Scientific), 4.5µL of PCR-grade water and 1.5µL of each primer (**Table 2**) at 10µM (Eurogentec); a total of 5µL of DNA was added to a 25µL final volume. PCR was performed onto a C1000™ thermal cycler with a CFX96™ detection module (BIORAD) with initial denaturation for 3 minutes at 95 °C followed by 30 cycles of denaturation at 94 °C for 30s, annealing at 55 °C for 60s, and extension at 72 °C for 60s. PCR products were purified using Nucleospin gel and PCR clean-up (Macherey-Nagel REF740609.250), and nucleic acid concentration was measured using Nanodrop. Amplicons sizes between 536 and 600 bp for the 7 genes were observed using Amersham ImageQuant 800 imager after migration and gel electrophoresis.

**Figure 1:**
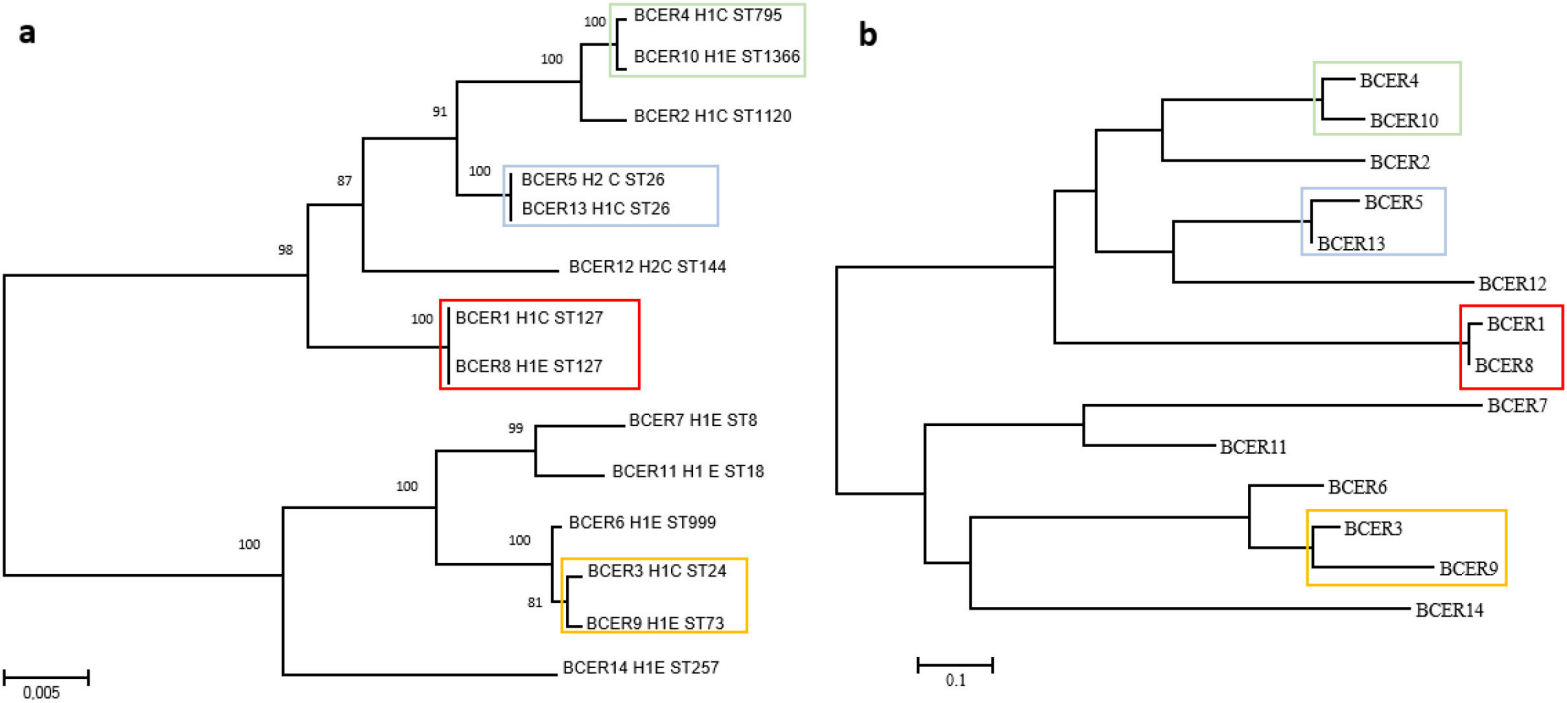
Phylogenetic analysis of *B. cereus* isolates. **(a)** Maximum likelihood phylogenetic tree constructed from concatenated sequences of the MLST scheme for the *B. cereus* isolates. **(b)** Neighbor-joining phylogenetic tree derived from ENUHCT data for the same set of *B. cereus* isolates.

PCR products were sequenced using the Sanger methodology (Azenta Life Sciences). Sequences were edited as recommended (*http://www.pubmlst.org/bcereus*) and consensus were obtained using CLC Genomics workbench 22 software. The MLST database of *B. cereus* was used for ST assignment. We constructed a Maximum Likelihood (ML) tree based on the phylogenetic analysis of the concatenated sequences of the seven alleles from both clinical and environmental isolates together with concatenated sequences from selected ST from the database (**Figure 1a**). Sequence alignment and phylogenetic analysis were done using MEGA11 (Molecular Evolutionary Genetics Analysis version 11, Tamura, Stecher, and Kumar 2021).

### EndoNUclease heteroduplex cleavage typing

**Figure 2:**
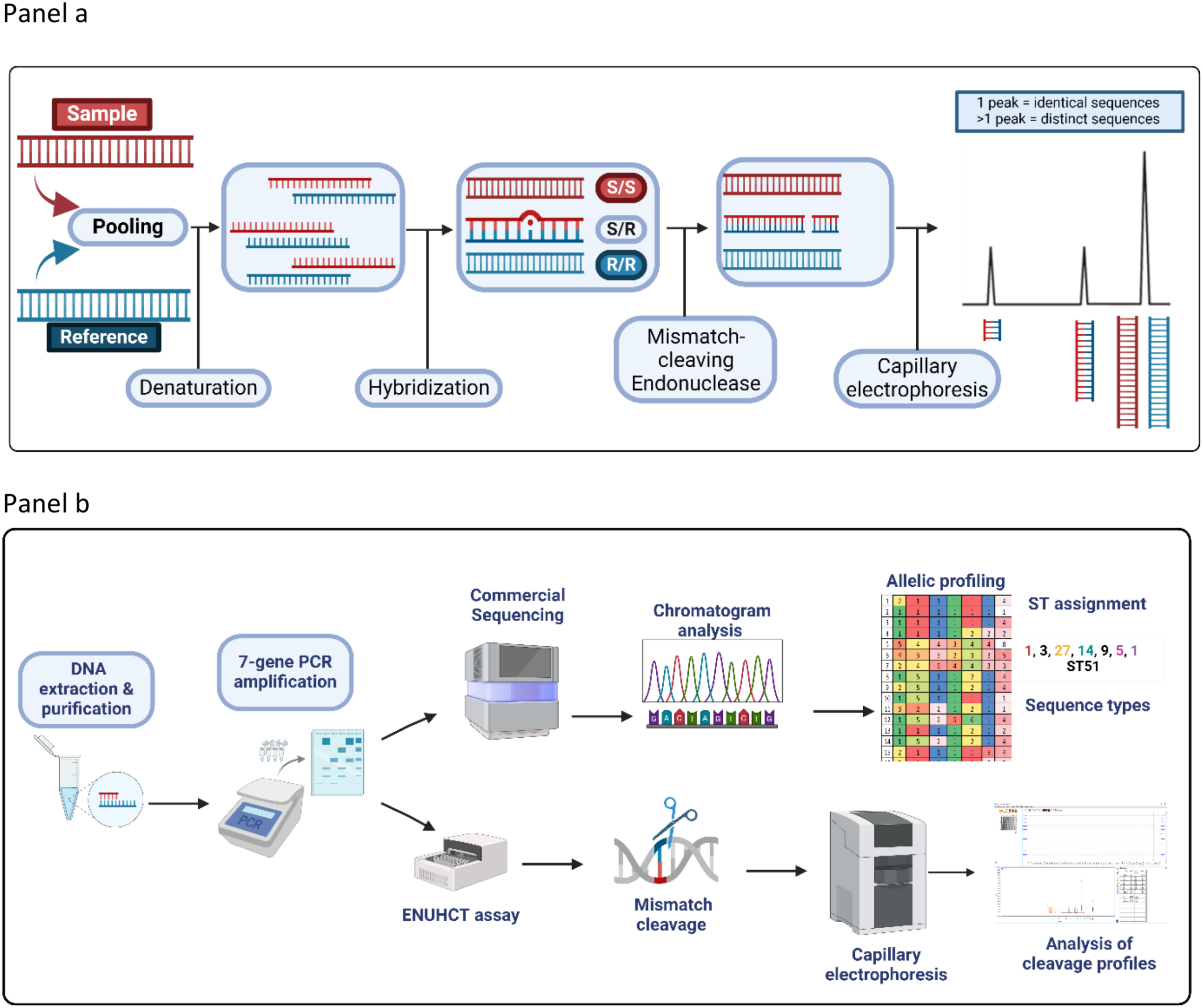
Experimental set up. **(a)** Schematic diagram illustrating the EndoNUclease Heteroduplex Cleavage Typing technique. **(b)** Comparison of sequencing-based MLST and ENUHCT-based MLST workflows.

Typing using a mismatch-specific endonuclease relies on the pooling, denaturation and re-hybridization of two PCR products to be compared, followed by their incubation with a mismatch-specific endonuclease that cleaves in the mismatch vicinity (**Figure 2a**). This technology, applied to microbial samples, named ENUHCT, was previously described (Pilato et al., 2012).

Briefly, 125-400ng of DNA from each of the two PCR products to be compared were mixed in a 10µL final volume and incubated for denaturation/hybridization starting at 95 °C for 10min, 85 °C for 60s, 75 °C for 60s, 65 °C for 60s, 55 °C for 60s, 45 °C for 60s, 35 °C for 60s, 25 °C for 60s. Then, enzymatic digestion was performed by adding 1µl Surveyor S endonuclease, 1µl enhancer, and 1,2µl MgCl_2_ (Surveyor^®^ IDT Kit) and a 60min incubation at 42 °C. Last, 1/10^th^ volume of the blocking solution was added to each reaction. Patterns of cleavage were observed by capillary electrophoresis using a fragment analyzer (Agilent Fragment Analyzer 5300) (**Figure 2**).

### ENUHCT theoretical profiles

When the pooled pair of amplicons have nucleotide sequences that are identical, they form homoduplexes after denaturation/hybridization cycles, therefore no endonuclease cleavage occurs which results in a single peak. When pooled amplicons have nucleotide variations, both homoduplexes (self-hybridization of PCR products, thus with no mismatch) and heteroduplexes (producing mismatches) are formed. Single nucleotide variations are cleaved and detected mismatch sites in the heteroduplexes, leading to cleavage patterns appearing after capillary electrophoresis and observed by distinct peaks as well as the entire fragment peak corresponding to the homoduplex.

From the MLST sequences, it was possible to predict cleavage profiles when PCR products of two strains were mixed and submitted to ENUHCT reactions. These theoretical cleavage profiles were generated in order to choose the most appropriate genes for ENUHCT testing, depending on the number of mismatches between the strains.

### ENUHCT experimental profiling

For pairwise comparison of each locus, the number of peaks observed on the electropherogram was automatically determined by the capillary electrophoresis software and validated by a visual inspection. The peaks corresponding to the homoduplexes were not considered. Only the number of peaks corresponding to cleaved heteroduplexes were retained for analysis. We next compiled the numbers of peaks for the 7 loci of each strain to obtain the value “T”, as the total number of peaks for the seven concatenated loci (total length L = 4095 bp). “T” was used to define a second value P= $\frac{100\cdot T}{L}$ reflecting the percentage of divergence between a pair of strains. In order to illustrate the experimental distance monitored by ENUCHT, a third value “I” where I = 100-P, defined as the percentage of identity between the sets of strains.

### Phylogenetic reconstruction using sequence analysis or ENUHCT peaks analysis

Gene sequences were concatenated and aligned using MEGA11. Panel a represents the phylogenetic analysis using the concatenated sequences of the MLST-derived scheme. The phylogenetic reconstruction is inferred by using the Maximum Likelihood method based on the Tamura-Nei model. The tree with the highest log likelihood is shown. The percentage of trees on which the associated taxa are clustered together is shown next to the branches (Bootstrap 100). Panel b is derived from the ENUHCT experimental profiling. The phylogenetic tree is inferred by using Neighbor-joining method (NJ). Bootstrap values are not applicable for the precomputed distance matrix directly subjected to NJ statistical method. Initial tree(s) for the heuristic search are obtained automatically by applying BioNJ algorithms to a matrix of pairwise distances estimated using the Maximum Composite Likelihood (MCL) approach and then selecting the topology with superior log likelihood value. The tree is drawn to scale, with branch lengths measured in the number of substitutions per site. Evolutionary analyses were conducted in MEGA11.

## Results

### Selected bacterial strains

The characteristics of the 14 strains of *B. cereus* used in this study are presented in **Table 1**. Hospital 1 (H1) faced a *B. cereus* infection cluster. One of the objectives of the study was to define whether this cluster was associated with a single strain, using MLST and ENUHCT and to compare the results obtained by the 2 techniques. For this, 11 samples from patients or from the environment, and associated with the epidemic, were collected (4 clinical and 7 environmental samples). In parallel, 3 samples unrelated to the epidemic were obtained from H1 (one sample) and from a second hospital (H2, two samples).

### PCR, multilocus sequencing, and ST assignment

Systematic amplification of the 7 loci of each of the 14 samples was performed. In addition, intraclonal variation was tested by typing 5 subcultures (SC) from each strain to determine the potential genetic heterogeneity within a single bacterial colony. Allele sequences and STs were analyzed, and identical profiles were obtained, confirming the absence of intra-clonal heterogeneity between isolated colonies in this study, at least in the pool of sequenced loci. We calculated the D value of the whole 7-gene MLST scheme for the 14 strains, using the formula considering the number of unrelated strains (N = 14) and number of identified STs (S = 12) as well as the proportion of each strain within each ST to obtain D = 0.978. The results of the strain identification by MLST sequencing are presented in **Table 3a**; it includes the numbers of alleles assigned to each ST. The combination of 7 alleles (*glpF*7*, gmk*8*, ilvD*16*, pta*13*, pur*2*, pycA*16 *and tpi*7) constitutes the barcode for the ST (e.g., ST8). This barcode is an allelic profile. Each strain is identified based on its allelic profile. Given their allelic profiles, the 14 strains were attributed to 12 different ST. **Table 3b** shows a detailed analysis of the number of polymorphic sites from each locus and provides the D values for each locus site. This was calculated for each locus separately to showcase the diversity of each gene. In H1, two strains (from one clinical and one environmental sample) were identified as ST127. Two other strains belong to ST26, with one being part of the ‘neonatology H1 cluster’ and the second isolated from a 1–5-year-old patient in a different hospital (H2). The remaining 10 ST each correspond to a single strain and were isolated either from a clinical sample or from an environmental specimen.

**Table 3a:** Allelic profiles of the 14 *B. cereus* strains.

Sequence type	Nb of strains	Alleles						
		*glpF*	*gmk*	*ilvD*	*pta*	*pur*	*pycA*	*tpi*
ST-8	1	7	8	16	13	2	16	7
ST-18	1	11	9	14	12	12	14	7
ST-24	1	12	8	9	14	11	12	10
ST-26	2	3	2	31	5	16	3	4
ST-73	1	13	8	9	14	9	12	31
ST-127	2	6	4	53	4	16	6	3
ST-144	1	67	2	63	5	36	3	4
ST-257	1	15	7	7	2	5	10	13
ST-795	1	51	2	21	5	19	3	2
ST-999	1	13	8	8	11	11	12	10
ST-1120	1	19	2	31	17	19	3	2
ST-1366	1	19	2	21	5	19	3	183

**Table 3b:** Discrimination index and the number of variations of nucleotide sequences per locus.

Locus	Nb of alleles	Analyzed sequence (bp)	Nb of polymorphic sites	D
*glpF*	10	372	16	0.9560
*gmk*	5	504	25	0.7582
*ilvD*	9	393	64	0.9341
*pta*	7	414	21	0.8681
*pur*	8	348	44	0.8901
*pycA*	6	363	43	0.7912
*tpi*	8	435	15	0.9231

The phylogenetic tree based on concatenated sequences showed that the 14 strains can be grouped into 7 clusters consisting of either 100% identical sequences ((BCER5 and BCER13 [ST26], BCER1 and BCER8 [ST127]) or most closely related strains (BCER4 and BCER10 [ST795 and ST1366] together with BCER2 [ST1120], BCER3 with BCER9 [ST24 and ST73]) together with BCER6 [ST999], BCER 7 and BCER11 [ST8 and ST18]); 2 strains remained as singletons (BCER12 [ST144] and BCER14 [ST257] (**Figure 1a**).

The MLST sequences were then used to produce a pairwise matrix and a phylogenic tree. To anticipate the comparison of the MLST by sequencing with the MLST by ENUCHT, we draw a matrix showing the number of SNPs. Each set of amplicons from the 14 strains was then run against the 13 others. **Table 4** showed the expected number of mismatches for each combination of strains based on the sequencing. The glpF, gmk, pta, pycA and tpi genes presented a relatively low variability rate compared to ilvD and pur, where the number of pairwise SNPs was 14 and 30 among two distant strains such as BCER12 [ST144] and BCER14 [ST257], respectively. We next generated a MLST sequence-based phylogenetic tree using the Maximum Likelihood method (**Figure 1**). The tree highlights the clustering of BCER1 and BCER8, and BCER5 and BCER13 in ST127 and ST26, respectively. In addition, it shows that ST795 and ST1366 on one hand, as well as ST24 and ST73 on the other hand, are related ST. In addition, BCER14 is the most distant strain compared to the others.

**Table 4:** Theoretical profiling of strains using the 7-gene scheme applied for ENUHCT. The tables are color coded (Purple: 0 SNP, red: 1 SNP, yellow: 2 SNP, green: 3 SNP, blue: 4 SNP, grey: 5SNP).

	*glp*															*pur*													
	1	2	3	4	5	6	7	8	9	10	11	12	13	14		1	2	3	4	5	6	7	8	9	10	11	12	13	14
1	0														1	0													
2	6	0													2	20	0												
3	6	6	0												3	11	13	0											
4	7	1	7	0											4	20	0	13	0										
5	5	2	6	3	0										5	0	20	11	20	0									
6	6	6	2	7	3	0									6	11	13	0	13	11	0								
7	3	3	5	6	5	5	0								7	16	12	7	12	16	7	0							
8	0	6	6	7	5	6	3	0							8	0	20	11	20	0	11	16	0						
9	6	6	2	7	3	0	5	6	0						9	10	11	1	11	10	1	6	10	0					
10	6	0	6	1	2	6	3	6	6	0					10	20	0	13	0	20	13	12	20	11	0				
11	6	6	3	7	1	3	2	6	3	6	0				11	10	12	9	12	10	9	8	10	8	12	0			
12	2	6	6	7	6	6	3	2	6	6	6	0			12	28	27	27	27	28	27	27	28	27	27	23	0		
13	5	2	6	3	0	4	5	5	4	2	1	6	0		13	0	20	11	20	0	11	16	0	10	20	10	28	0	
14	6	6	2	7	6	2	5	6	2	6	4	6	6	0	14	15	13	8	13	15	8	11	15	7	13	11	30	15	0
	*gmK*															*pycA*													
	1	2	3	4	5	6	7	8	9	10	11	12	13	14		1	2	3	4	5	6	7	8	9	10	11	12	13	14
1	0														1	0													
2	2	0													2	2	0												
3	22	22	0												3	31	20	0											
4	2	0	22	0											4	2	0	20	0										
5	2	0	22	0	0										5	2	0	20	0	0									
6	22	22	0	22	22	0									6	31	20	0	20	20	0								
7	22	22	0	22	22	0	0								7	31	20	4	20	20	4	0							
8	0	2	22	2	2	22	22	0							8	0	2	31	2	2	31	31	0						
9	22	22	0	22	22	0	0	22	0						9	31	20	0	20	20	0	4	31	0					
10	2	0	22	0	0	22	22	2	22	0					10	2	0	20	0	0	20	20	2	20	0				
11	23	23	1	23	23	1	1	23	1	23	0				11	31	20	4	20	20	4	5	31	4	20	0			
12	2	0	22	0	0	22	22	2	22	0	23	0			12	2	0	20	0	0	20	20	2	20	0	20	0		
13	2	0	22	0	0	22	22	2	22	0	23	0	0		13	2	0	20	0	0	20	20	2	20	0	20	0	0	
14	22	20	3	20	20	3	3	22	3	20	4	20	20	0	14	31	20	13	20	20	13	14	31	13	20	17	20	20	0
	*ilvD*															*tpi*													
	1	2	3	4	5	6	7	8	9	10	11	12	13	14		1	2	3	4	5	6	7	8	9	10	11	12	13	14
1	0														1	0													
2	14+	0													2	1	0												
3	14+	14+	0												3	18	7	0											
4	14+	5	14+	0											4	1	0	7	0										
5	14+	0	14+	5	0										5	3	2	5	2	0									
6	14+	14+	1	14+	14+	0									6	18	7	0	7	5	0								
7	14+	14+	14+	14+	14+	14+	0								7	7	6	1	6	3	1	0							
8	0	14+	14+	14+	14+	14+	14+	0							8	0	1	18	1	3	18	7	0						
9	14+	14+	0	14+	14+	1	14+	14+	0						9	9	8	1	8	6	1	2	9	0					
10	14+	14+	14+	0	14+	14+	14+	14+	14+	0					10	2	1	8	1	3	8	7	2	9	0				
11	14+	14+	14+	14+	14+	14+	2	14+	14+	14+	0				11	7	6	1	6	3	1	0	7	2	7	0			
12	14+	14+	14+	14+	14+	14+	14+	14+	14+	14+	14+	0			12	3	2	5	2	0	5	3	3	6	3	3	0		
13	14+	0	14+	5	0	14+	14+	14+	14+	5	14+	14+	0		13	3	2	5	2	0	5	3	3	6	3	3	0	0	
14	14+	14+	14+	14+	14+	14+	14+	14+	14+	14+	14+	14+	14+	0	14	11	10	7	10	10	7	6	11	8	11	6	10	10	0
								*pta*																					
								1	2	3	4	5	6	7	8	9	10	11	12	13	14								
							1	0																					
							2	7	0																				
							3	6	11	0																			
							4	1	6	6	0																		
							5	1	6	6	0	0																	
							6	6	11	2	7	7	0																
							7	8	14	5	10	10	3	0															
							8	0	7	6	1	1	6	8	0														
							9	6	11	0	6	6	2	5	6	0													
							10	1	6	6	0	0	7	10	1	6	0												
							11	6	12	2	8	8	1	2	6	3	8	0											
							12	1	6	6	0	0	7	10	1	6	0	8	0										
							13	1	6	6	0	0	7	10	1	6	0	8	0	0									
							14	9	12	11	10	10	9	12	9	11	10	10	10	10	0								

### EndoNUclease heteroduplex cleavage typing

To assess the relevance of ENUHCT in identifying polymorphism among samples, systematic pairwise experiments were performed between the 7 PCR amplified gene regions of the 14 samples. The results of capillary electrophoresis after pairwise PCR mixing, annealing and cleavage by mismatch-specific endonuclease (description in **Figures 2a, b**) were collected, and peaks corresponding to each experiment were recorded. The analysis of the results was performed with no *a priori*, with three objectives: identification of strains clustered in common ST, identification of related ST, evaluation of the genetic distance diversity.

### MLST-derived ENUHCT theoretical profiling

**Table 4** shows the theoretical profiling of the *B. cereus* strains using the 7-gene scheme applied for ENUHCT representing the number of mismatches between 2 strains. The table is color coded (Purple: 0 SNP, red: 1 SNP, yellow: 2 SNP, green: 3 SNP, blue: 4 SNP, grey: 5SNP).

### ENUHCT experimental profiling

The pairwise matrix is presented in **Table 5a** where the samples were classified as (1) identical strains, (2) related strains and (3) independent strains. Strains clustered in a common ST can be identified with I = 100 and P = 0.

**Table 5a:** Distance matrix representing the estimates of evolutionary divergence between nucleotide sequences.

	BCER1	BCER2	BCER3	BCER4	BCER5	BCER6	BCER7	BCER8	BCER9	BCER10	BCER11	BCER12	BCER13	BCER14
BCER2	0.021													
BCER3	0.045	0.049												
BCER4	0.019	0.004	0.047											
BCER5	0.012	0.011	0.046	0.011										
BCER6	0.045	0.049	0.002	0.047	0.046									
BCER7	0.048	0.049	0.015	0.047	0.048	0.015								
BCER8	0	0.021	0.045	0.019	0.012	0.045	0.048							
BCER9	0.045	0.049	0.001	0.047	0.046	0.002	0.015	0.045						
BCER10	0.019	0.004	0.047	0.001	0.011	0.047	0.047	0.019	0.047					
BCER11	0.046	0.049	0.014	0.047	0.046	0.014	0.007	0.046	0.014	0.047				
BCER12	0.018	0.019	0.052	0.019	0.016	0.052	0.051	0.018	0.052	0.019	0.051			
BCER13	0.012	0.011	0.046	0.011	0	0.046	0.048	0.012	0.046	0.011	0.046	0.016		
BCER14	0.047	0.049	0.026	0.047	0.049	0.026	0.028	0.047	0.026	0.047	0.028	0.052	0.049	0

**Table 5b:** Experimental ENUHCT matrix representing the I value between the 14 strains (top right) and the percentage of divergence between sequences (bottom left).

	BCER1	BCER2	BCER3	BCER4	BCER5	BCER6	BCER7	BCER8	BCER9	BCER10	BCER11	BCER12	BCER13	BCER14
BCER1		99.072	98.608	99.096	99.048	98.266	98.095	100	98.413	99.170	98.608	99.048	99.096	98.291
BCER2	0.928		98.950	99.341	99.292	98.535	98.437	98.877	98.755	99.585	99.048	99.048	99.389	98.559
BCER3	1.392	1.050		98.632	98.730	99.707	98.681	98.486	99.805	98.559	99.341	98.437	98.462	98.559
BCER4	0.904	0.659	1.368		99.536	98.535	98.339	98.926	98.413	99.902	98.755	99.145	99.609	98.364
BCER5	0.952	0.708	1.270	0.464		98.486	98.266	98.974	98.413	99.292	98.755	99.292	100	98.657
BCER6	1.734	1.465	0.293	1.465	1.514		98.926	98.901	99.731	98.364	99.292	98.388	98.901	99.023
BCER7	1.905	1.563	1.319	1.661	1.734	1.074		98.510	98.437	98.315	99.292	98.462	98.388	98.730
BCER8	0.000	1.123	1.514	1.074	1.026	1.099	1.490		98.510	99.048	98.413	98.803	99.219	98.535
BCER9	1.587	1.245	0.195	1.587	1.587	0.269	1.563	1.490		98.584	98.779	98.291	98.364	98.974
BCER10	0.830	0.415	1.441	0.098	0.708	1.636	1.685	0.952	1.416		98.779	99.121	99.414	98.584
BCER11	1.392	0.952	0.659	1.245	1.245	0.708	0.708	1.587	1.221	1.221		98.730	98.828	98.632
BCER12	0.952	0.952	1.563	0.855	0.708	1.612	1.538	1.197	1.709	0.879	1.270		99.536	98.388
BCER13	0.904	0.611	1.538	0.391	0.000	1.099	1.612	0.781	1.636	0.586	1.172	0.464		98.510
BCER14	1.709	1.441	1.441	1.636	1.343	0.977	1.270	1.465	1.026	1.416	1.368	1.612	1.490	

**Figure 3:**
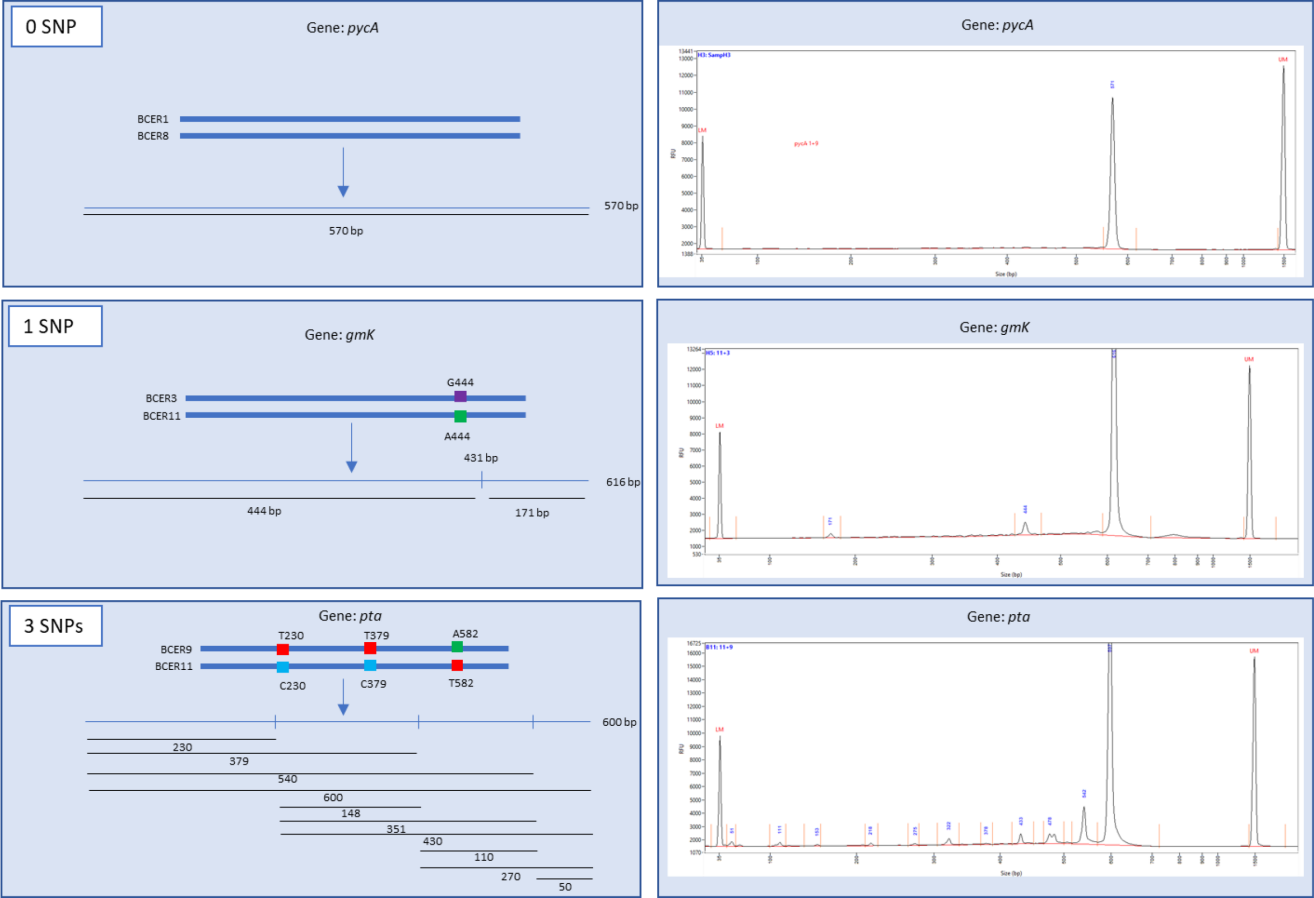
ENUHCT cleavage profiles for varying SNP differences. Examples of results obtained by ENUHCT showing theoretical (left) and experimental (right) cleavage profiles for 0, 1, and 3 single-nucleotide polymorphisms (SNPs), respectively.

**Figure 4:**
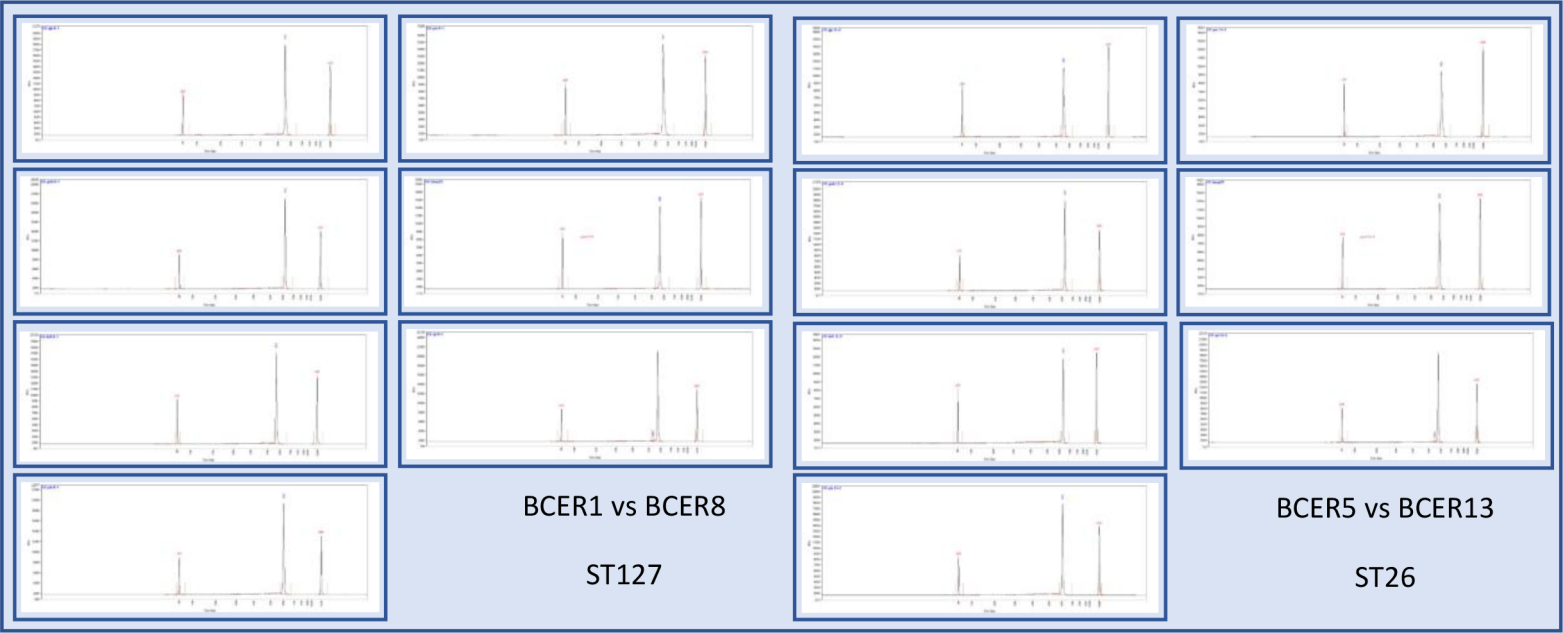
Examples of absence of cleavage at specific sequence types. Representative electropherograms showing absence of ENUHCT cleavage for ST26 (comparison of BCER5 vs BCER13) and ST127 (comparison of BCER1 vs BCER8).

**Figure 5:**
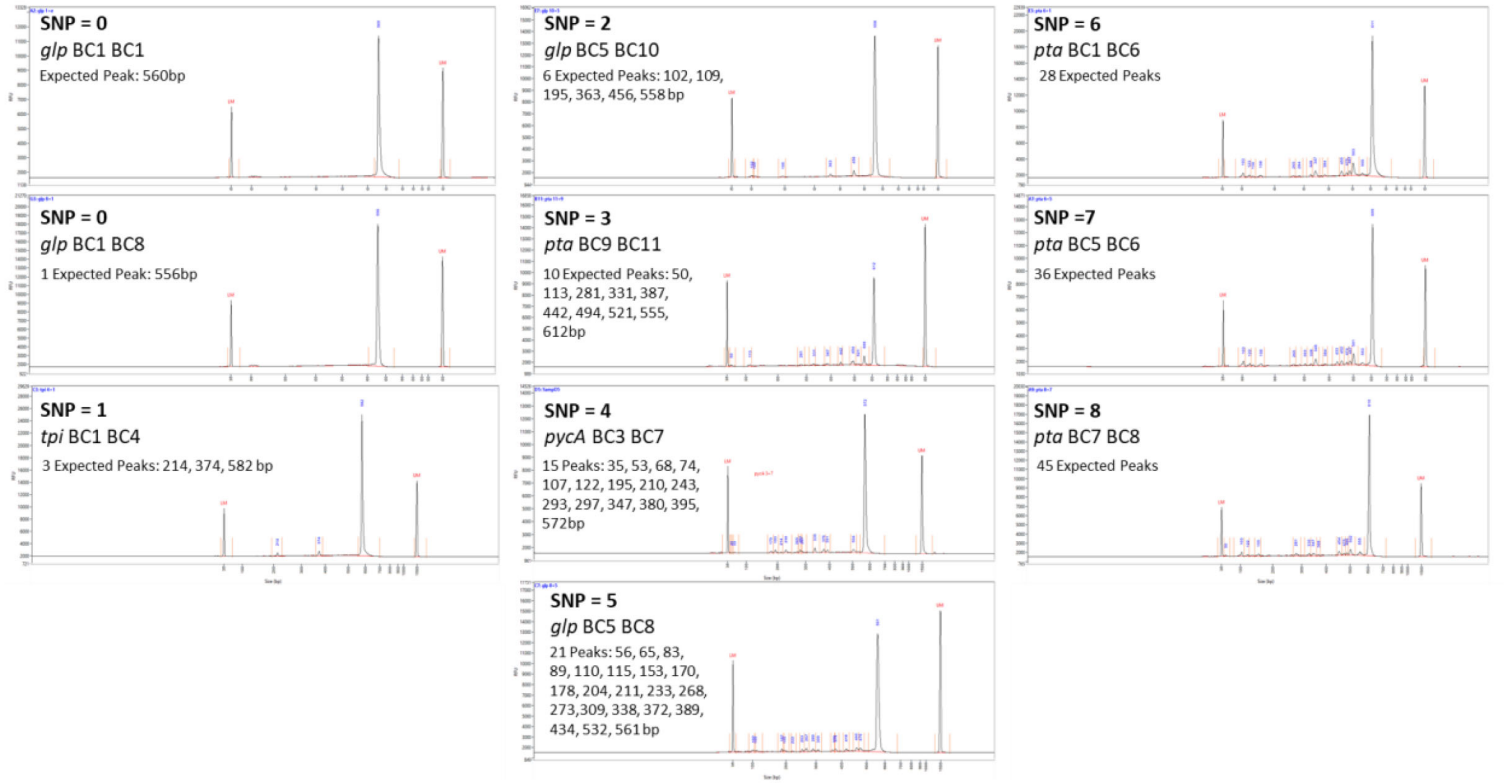
Correlation of SNP number with observed peak patterns. Correlation between the number of SNPs and the corresponding observed electrophoretic peak profiles for selected *B. cereus* strains.

**Figure 3** shows an example of the cleavage profiles obtained when PCR products corresponding to sequences resulting in 0, 1 and 3 SNPs were compared theoretically and experimentally: the theoretical prediction was confirmed experimentally. The absence of mismatch resulted in an absence of cleavage displayed by a unique peak (also shown in **Figure 4** for BCER1 *vs* BCER8 [ST127] and BCER5 *vs* BCER13 [ST26]). When one SNP was identified by MLST, at least two products of the mismatch-specific cleavage were observed leading to the systematic detection of the SNP. The existence of 2, 3, 4, and 5 SNPs between the two sequences (**Figure 5**) resulted in the production of 6, 10, 15 and 21 peaks. **Figure 5** represents examples of the profiles observed (i) when a strain was challenged against itself (0 cleavage), (ii) when a strain was challenged against a strain belonging to the same ST (0 cleavage), (iii) when a strain was challenged against strains displaying an increasing level of diversity (1–5 cleavages). **Supplementary Figure C** showed 68 profiles of paired comparative analysis for the seven genes.

**Figure 6:**
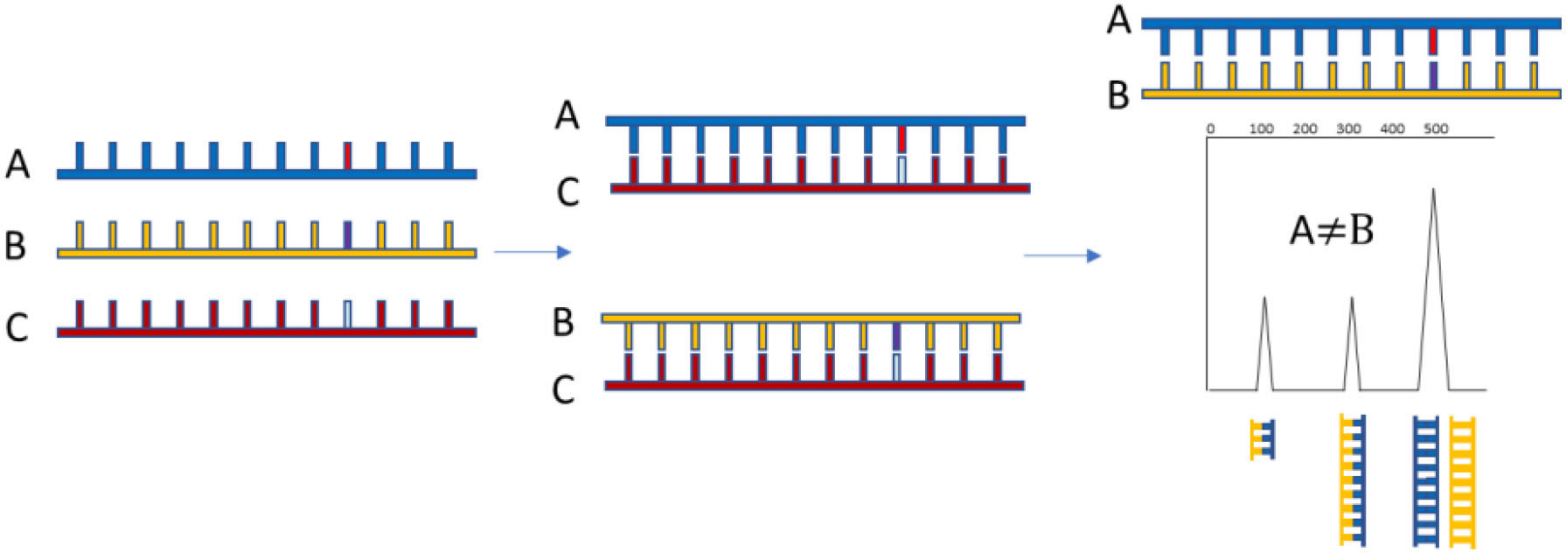
Effects of multiple mutations at the same site. Illustration of the case where two different point mutations occur at the same nucleotide position, as revealed by ENUHCT cleavage pattern analysis.

We also explored how enzymatic cleavage can differentiate between two DNA sequences (strains A and B) that have substitution mutations at the same position, but with different nucleotides. When PCR products from sequences A and B for example were each hybridized with the one of a different sequence C, the enzyme cleaves at the mismatched site, producing identical-length fragments for A and C as well as B and C, which may falsely suggest A and B are the same (**Figure 6**).

To resolve this, A and B are hybridized directly. Since A and B had different mutations, their heteroduplex formed a mismatch, allowing the enzyme to cleave, hence confirming they were distinct sequences. This strategy clarified the ambiguity from the identical-length cleavage products.

### Sequence based matrix vs ENUHCT based matrix

**Figure 7:**
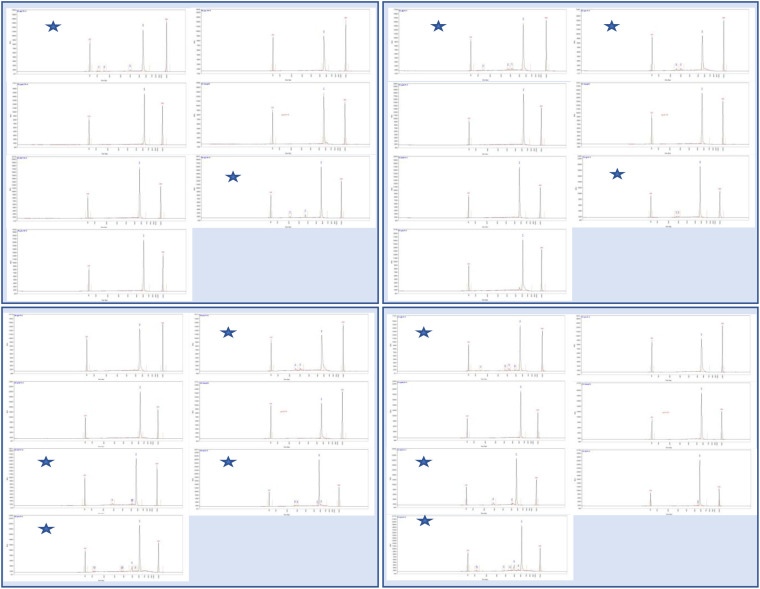
ENUHCT profiles of related *B. cereus* strains. Profiles of related strains: Top left: BCER4 vs BCER10; Top right: BCER9 vs BCER3; Bottom left: BCER9 vs BCER6; Bottom right: BCER3 vs BCER6. Different loci are marked with an asterisk (*).

Using the MLST sequences, 10 *B. cereus* strains were distinct from each other; on another hand, BCER5 and BCER13 belonged to ST26 whereas BCER1 and BCER8 belonged to ST127. Although slightly different from each other BCER4 and BCER10 were most closely related as well as BCER3 and BCER9. Using the ENUHCT results and corresponding I-value matrix BCER the overall grouping was very similar to that using sequence data (tight grouping of the 4 aforementioned pairs) although matrix analysis was more informative than phylogenetic reconstruction for defining pairs belonging to the same ST. BCER4 and BCER10 (I = 99,902) for example, differ by 2 out of 7 loci (*glpF and tpi*) but showed identical cleavage profiles for the 5 other loci (**Figure 7**). Similarly, BCER9 and BCER3 differed by 3 loci (*glpF, pur*, *tpi*) out of 7 (**Figure 7**). BCER9 and BCER6 also shared 3 identical loci (*glpF, gmk, pycA*), whereas BCER6 shared 4 loci with BCER3.

### Phylogenetic reconstruction using sequence analysis or ENUHCT peaks analysis

The phylogenetic tree derived from the distance matrix (**Table 5b**) is presented in **Figure 1a**. The 14 strains grouped into two main clusters of 8 strains and 6 strains, respectively, with bootstrap values >81%. The two pairs of identical strains BCER5/BCER8 (ST26) and BCER1/BCER8 (ST127) belonged to the 8-strain group. Beside the two pairs BCER4/BCER10 on one hand and BCER3/BCER9 on the other hand were most closely related to each other although they were attributed to different ST. Finally, the two strains corresponding to Hospital 2 are distinct from each other.

The topology observed from ENUHCT I-value based phylogenetic reconstruction data analysis (**Figure 1b**) was clearly highly similar compared with that of **Figure 1a**. Due to the innate nature of the ENUHCT dataset, bootstrap pseudo-replication was not possible. Although the initial objective was not to use this dataset for phylogenetic reconstruction, the comparison of the shape of both trees suggests that ENUHCT data could be used to go farther than the simple pairwise comparison that was the primary goal of the study.

In summary, both trees showed ST clustering and relatedness among strains, but they differed in genetic distance and branching patterns for distant strains. These results indicated that while the ENUHCT could generate a phylogenetic tree like the MLST-derived one, it might not fully capture the genetic relationships among strains without prior sequencing. The clustering of strains with identical or related sequence types was evident, but the weaker support for some relationships pointed out the limitations of relying solely on ENUHCT for thorough phylogenetic analysis.

## Discussion

As a poof of principle for the applicability of ENUHCT to epidemiological typing, we selected the example of *B. cereus* for several reasons: First, due to the importance of *B. cereus* as a recent nosocomial pathogen and the need for rapid typing in hospital settings. Secondly, the efficiency of the chosen scheme, in fact, 3354 *B. cereus* profiles were indexed in pubMLST until February 2025, with new profiles of *B. cereus* regularly updating the database. Thirdly, for species with high mutation rates like *B. cereus*, related strains identified within a short period of time are likely to be linked epidemiologically. Finally, developing a double-matrix system to analyze results by comparing *B. cereus* samples one by one eliminates the need for a reference sample, as each sample is run against all the others.

The aim of this study was to assess **(i)** whether ENUHCT was able to provide rapid analysis that is congruent with MLST analysis, **(ii)** whether the MLST-derived ENUHCT theoretical results were matching with the ENUHCT experimental results, **(iii)** and whether ENUHCT was adapted to timely screening for sorting the strains with identical profiles in order to detect patient to patient or environment to patient transmission in hospital associated infections (HAI) context and to select strains needing full genome sequencing confirmation. In this context, we took advantage of a real-life situation (14 strains of *B. cereus* that had been typed by MLST for tracing purpose) in order to demonstrate the feasibility and possible added value of ENUHCT as an option to replace MLST or filter strains needing to be typed further with MLST or other genomic techniques.

- The first point was to assess whether ENUHCT was able to provide rapid analysis that was congruent with MLST results. ENUHCT can provide results within hours after the strain is isolated on agar plate; it is adapted to a timely tracking of cases to define whether further full genome analysis is needed and for what strains further investigation is necessary. Typing results on the 7 loci of the typing scheme of MLST with no prior sequencing data, were similar to those obtained using sequencing results from MLST. Namely, the strains belonging to the same ST were indistinguishable when looking at the ENUHCT profiles; in contrast strains belonging to different ST displayed distinct ENUHCT profiles. This result was not unexpected from the theoretical point of view since ENUHCT is using the same PCR products as the ones generated with the MLST primers. However, there is no previous study validating the use of mismatch-cleavage enzyme for MLST, and limitations could be anticipated during the process, such as, efficacy of the PCR, unbalanced mix of PCR products, non-specific activity of the enzyme, low sensitivity of the capillary electrophoresis, or automatic threshold applied for the selection of the peaks. None of these potential technical limitations was observed and we could validate for the first time the use of mismatch identification for the clustering of bacterial strains in STs. The main advantage of ENUHCT resides in the fact that strains are compared by pairs for each gene so that they can be declared distinct as soon as there is at least one peak denoting at least one cleavage indicative of genetic difference in the two sequences. Hence, the comparison of two strains can be stopped as soon as the analysis of one gene demonstrates that both sequences are different; in this case there is no need to pursue the analysis for other genes. Regarding this point, only the MLST identical strains will need to be compared by using the 7 genes through ENUHCT analysis.
- The second point was to assess whether the MLST-derived ENUHCT theoretical results based on MLST data were matching with the ENUHCT experimental results. We targeted 3 main objectives to evaluate our methodology. (1) Identifying strains with identical sequence types (STs). Of the 11 strains that were assumed to be epidemiologically linked, two belonged to ST127 (BCER1 and BCER8) and were not cleaved at the ENUHCT level for any of the 7 genes (**Figures 1**, **3**). The same applied for BCER5 and BCER13, both belonging to ST26 (**Figures 1**, **3**) despite they were not epidemiologically related (**Table 1**). These strains also clustered in the phylogram based on the ENUHCT matrix (I = 100) (**Figure 1b**). It is important to distinguish between the percentage of identity in MLST (theoretical analysis) and the identity value ‘I’ (experimental distance) in ENUHCT. In theory, the ‘I’ value represents the percentage of identity obtained by identifying mismatches between two sequences. However, in the pairwise matrix of ENUHCT, to reach the first objective of identifying identical sequences, identity is inferred from the absence of peak detection. (2) ENUHCT experimental results were also able to generate closely related STs, helping to infer genetic relationships between strains. Beyond grouping the samples into identical STs, limited polymorphism among closely related strains was observed, despite them having distinct sequence types. (3) Last, we demonstrated that a phylogenetic tree can be constructed from the distance matrix generated by ENUHCT, which is based on the number of peaks. This is of utmost importance in the routine application of the ENUHCT as phylogenetic representations are very easy to understand for non-experts and are sound for anybody in the medical and paramedical and administration staff. Phylogenetic analysis without the need for sequencing allows for prioritizing distinct or ambiguous strains for further selection and sequencing and possibly leading to the discovery of new STs. Although ENUHCT yields similar results compared to MLST for identical and related strains; it has a limitation: when the number of mismatches is ≥4, the number of automatically collected peaks tends to be underestimated (**Supplementary Figure C**), thus modifying the distances between the strains, and deviating the distances, and possibly the branching in the phylogram (**Figures 1a, b**). Consequently, for more than 4 SNPs in a single fragment, where more than 15 fragments were obtained, we had to halt peak count even though we still were able to observe the aspect of the different peaks for the pairs of strains. An additional aim was to explore the potential for reducing the number of loci required for accurate clustering. ENUHCT relies on quality and quantity of DNA fragments for optimal peak detection and accurate analysis. When genes have high number of mismatches, they can create numerous small fragments, making it harder to identify peaks due to reduced fluorescence. BCER14 was identified as the most genetically distinct strain among the 13 examined (**Table 4**); this reduced peak number can affect phylogenetic analysis, evident when BCER14 clustered distantly with BCER6, BCER3, and BCER9 in experimental results, unlike its unrelated grouping in the theoretical model (**Figures 1**, **3**). To reduce the number of selected gene candidates, we evaluated the experimental variability of each gene. Among our samples, *gmk* and *pycA* were the least variable genes which is in line with the online data available for these alleles. Genes such as *glpF*, *pta* and *tpi* were typically seen as moderately stable among the selected strains (**Table 3b**) as well as in the public database. Their consistent amplification and sequence divergence help produce clearer peaks, improving the reliability of clustering results. On the other hand, genes like *ilvD* tend to have higher variability, hindering peak detection and clustering accuracy. A combination of stable genes like *pycA* with one or more other moderately variable genes, such as *tpi*, *glpF* or *pta* (respectively) could be considered for a first line screening with the aim of identifying the presence of non-related strains (**Supplementary Table B**).
- The third point was to assess whether ENUHCT was adapted to timely screening for sorting the strains with identical profiles to detect patient to patient or environment to patient transmission in HAI context and to select strains needing full genome sequencing confirmation. Regarding the turnaround time, ENUHCT is obviously faster than MLST with results obtained in less than 4 hours for the first plate; on a practical point of view, comparative analysis of the strains can be obtained within one day after the colonies are visible on the agar plate. MLST analysis needs to perform 14 strains x 7 genes, so that 98 PCR products must be sequenced in both directions ending with a total of 196 sequences to be verified, aligned and analyzed. With a total of 14 strains, each ENHUCT plate can incorporate the 91 samples (in the 96-well microplate) corresponding to the 91 possibilities of the matrix for each gene (**Supplementary Table A**). ENUHCT analysis is 70 min per run. The 7 genes can be tested sequentially, a strategy that offers the opportunity to identify strains that are different from each other and can be excluded from nosocomial acquisition. ENUHCT profiles can be obtained within 4 hours after PCR amplification that is a shorter turnaround time (1 working day) than that observed for MLST (more commonly 3–4 working days). These results showed that the genetic diversity of *B. cereus* can be timely studied during the outbreak, without additional steps like sequencing, to better match with the epidemic progression. Since strains that are displaying different profiles cannot belong to the same ST, they do not require further genetic analysis such as whole genome sequencing to confirm that they are distinct from one each other. Therefore, efforts can concentrate on strains displaying no ENUHCT cleavage to continue testing for additional genes and ultimately for complete genome analysis.

The benefit of ENUHCT is that it enables quick screening of strains to determine which ones will need further investigation for identity confirmation. ENUHCT analysis can be applied to any micro-organisms for which MLST primers have been described without the need for technical adjustment. Although it was not the objective of this study, ENUHCT has since been applied locally to Staphylococcus haemolyticus with the same success (**Supplementary Data D**).

The limits of ENUHCT are the limits of MLST. Bacteria species that can exchange resistance genes plasmid-mediated or transposon-mediated do not appear as efficient types. The latter seems to be less suited for ENUHCT since they are less suited for MLST analysis. In contrast, the interest of ENUHCT appears for bacterial species such as Neisseria meningitidis, Listeria monocytogenes, Escherichia coli O157:H7, group B Streptococcus and antibiotic-resistant strains of S. aureus. In the case of *B. cereus*, many factors contribute to the relatedness between clinical and environmental strains, including hospital location, degree of compliance with hospital hygiene practices, and the nature of the *B. cereus* strains present in the hospital. A recent study found that many *B. cereus* isolates in a neonatal hospital outbreak exhibited little to no significant genetic relatedness, highlighting the organism’s ubiquitous nature, and suggesting a high degree of genetic diversity among the isolates sampled from different sources of a neonatal environment. This genetic diversity was highlighted in previous studies, underscoring the value of high-resolution typing, in understanding the genetic diversity of *B. cereus* (Liu et al., 2017; Tönnies et al., 2024). Since the mutation rate differs from one species to another, typing methods should be tested accordingly. *B. cereus* exhibits high but relatively stable rates compared to other species, indicating that similar isolates related in short periods of time are presumably linked epidemiologically (Ceuppens et al., 2013). Additionally, ENUHCT requires access to a capillary electrophoresis machine that is not commonly distributed in diagnostic laboratories. An increasing number of compact and cost-effective CE platforms is on the market (Qsep 1 Bio−Fragment Analyzer and Qsep 1 Plus Advanced Portable Bio−Fragment Analyzer [CravitySci, USA], ChipGenie E2 and ZipChip Microfluidic [Repligen, USA], Bioanalyzer 2100 [Agilent, USA]). Although these systems were not evaluated in the present study, they are based on broadly similar underlying technical principles.

## Conclusion

This study demonstrates that ENUHCT is a rapid and effective alternative to MLST for *B. cereus* strain discrimination. ENUHCT successfully identified identical and closely related strains, aligning with MLST results, while providing results within hours instead of days. Its ability to detect single nucleotide polymorphisms (SNPs) through mismatch cleavage makes it a valuable tool for real-time outbreak tracking and infection control. However, ENUHCT’s accuracy diminishes with high SNP counts (≥4), limiting its resolution for highly divergent strains. Despite this, ENUHCT serves as an efficient first line screening method, reducing the need for full genome sequencing in unrelated strains. Its adaptability to other pathogens with established MLST schemes further enhances its utility in clinical microbiology. ENUHCT’s rapid turnaround supports timely decision-making during outbreaks, though further validation is needed for broader application. Overall, ENUHCT represents a significant advancement in rapid bacterial typing, offering a practical solution for real-time epidemiological investigations.

## Data Availability

The datasets presented in this study can be found in online repositories. The names of the repository/repositories and accession number(s) can be found in the article/**Supplementary Material**.
