# Supplementary material for: EndoNUclease Heteroduplex cleavage typing a new technique for rapid typing of bacterial isolates in the context of nosocomial outbreaks: proof of concept with Bacillus cereus
Source: Front Cell Infect Microbiol. 2026 Jun 9;16:1813523. doi: 10.3389/fcimb.2026.1813523 (PMC13287031; doi:10.3389/fcimb.2026.1813523)

**SUPPLEMENTARY TABLE A: Maximum capacity of testing per plate depending on number of strains**

| *Nb of strains* | *Nb of pairs per gene* | *Nb of gene(s) that can be processed per plate* |
| --- | --- | --- |
| *14* | *14x13 = 182/2 = 91* | *1* |
| *10* | *10x9 = 90/2 = 45* | *2* |
| *8* | *8x7 = 56/2 = 28* | *3* |
| *7* | *7x6 = 42/2 = 21* | *4* |
| *6* | *6x5 = 30/2 = 15* | *6* |
| *5* | *5x4 = 20/2 = 10* | *7* |

** 180 min TAT for processing one plate, each additional plate requires 70 min if*

*denaturation/rehybridization (20 min) and enzymatic digestion (60 min) have been done.*

This table shows the ENUHCT flow capacity for 7 genes analysis depending on the number of strains

under investigation. It indicates the number of strains and genes that can be included in one 4-

hour run of ENHUCT.

**SUPPLEMENTARY TABLE B: Classification of Allele Numbers (variability) in the paper vs. in the Database**

| Locus | Unique Allele Count in  sample population  (N=14) | Allele Count in  Database |
| --- | --- | --- |
| *glpF* | 9 | 452 |
| *gmK* | 9 | 499 |
| *ilvD* | 8 | 456 |
| *Pta* | 8 | 408 |
| *Pur* | 8 | 370 |
| *pycA* | 6 | 338 |
| *tpi* | 5 | 276 |

**SUPPLEMENTARY FIGURE C: Cleavage profiles of processed alleles: Representative peaks using the fragment analyzer system with dsDNA 910 Reagent Kit. Method: DNF-910-22 (ultra-short array)**

Gene: *glpF*

BCER12 (allele 67) vs BCER1 (allele 6): 2 SNP expected peaks 99, 200, 250, 299, 359, 556 bp

BCER12 (allele 67) vs BCER8 (allele 6): 2 SNP expected peaks 99, 200, 250, 299, 359, 556 bp


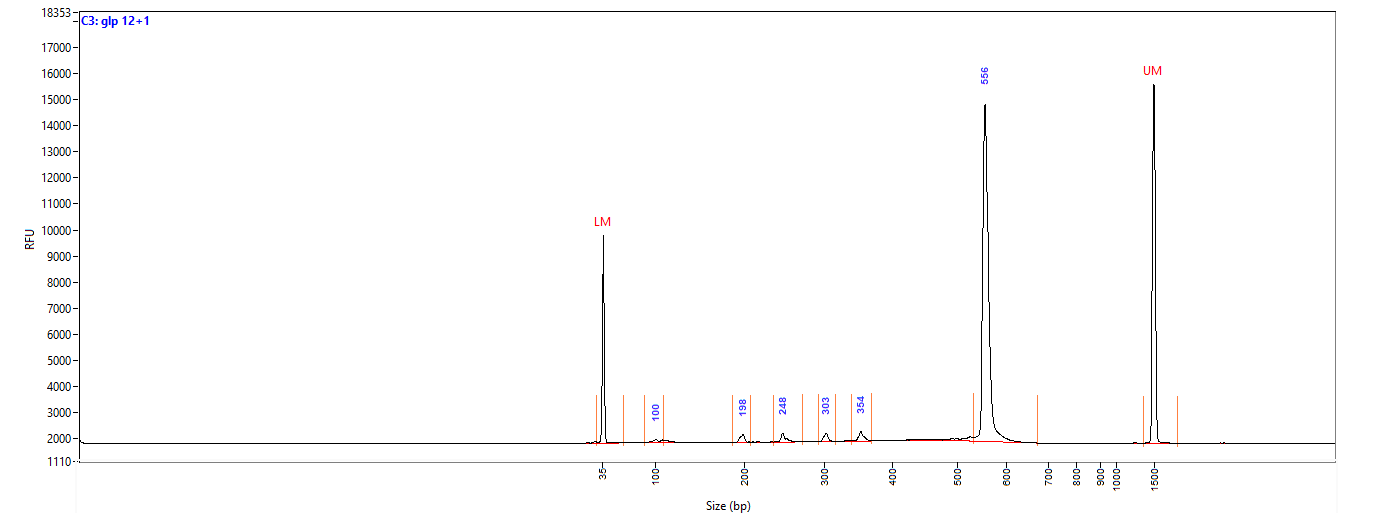

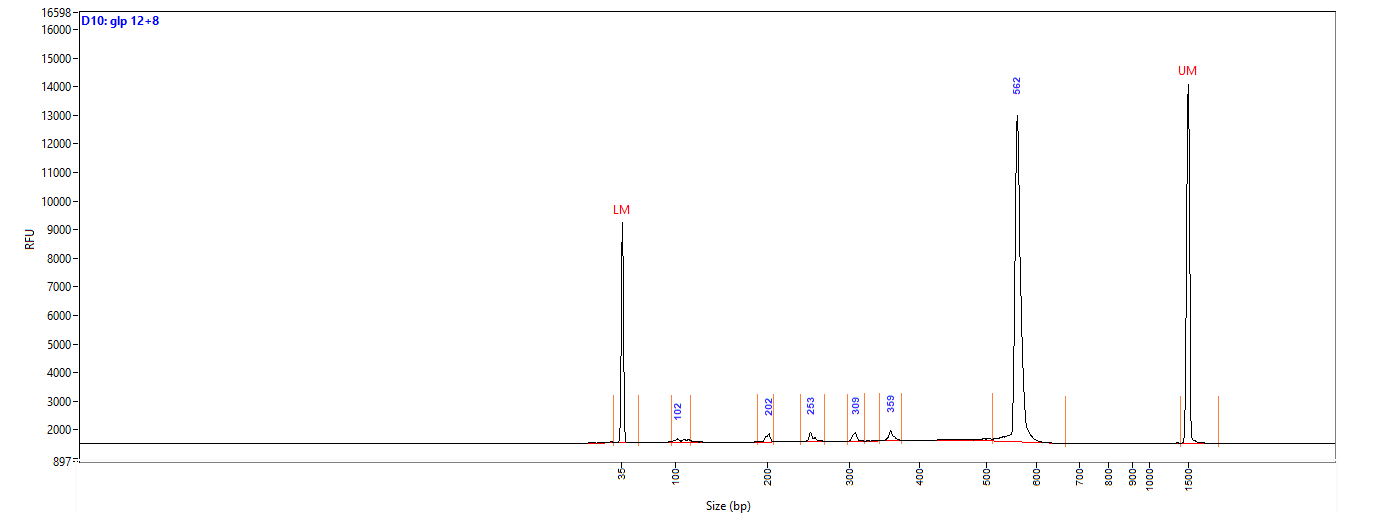


BCER1 (allele 6) vs BCER8 (allele 6): 0 SNP

BCER2 (allele 19) vs BCER5 (allele 3): 2 SNP expected peaks 25, 100, 110, 198, 365, 458, 560 bp


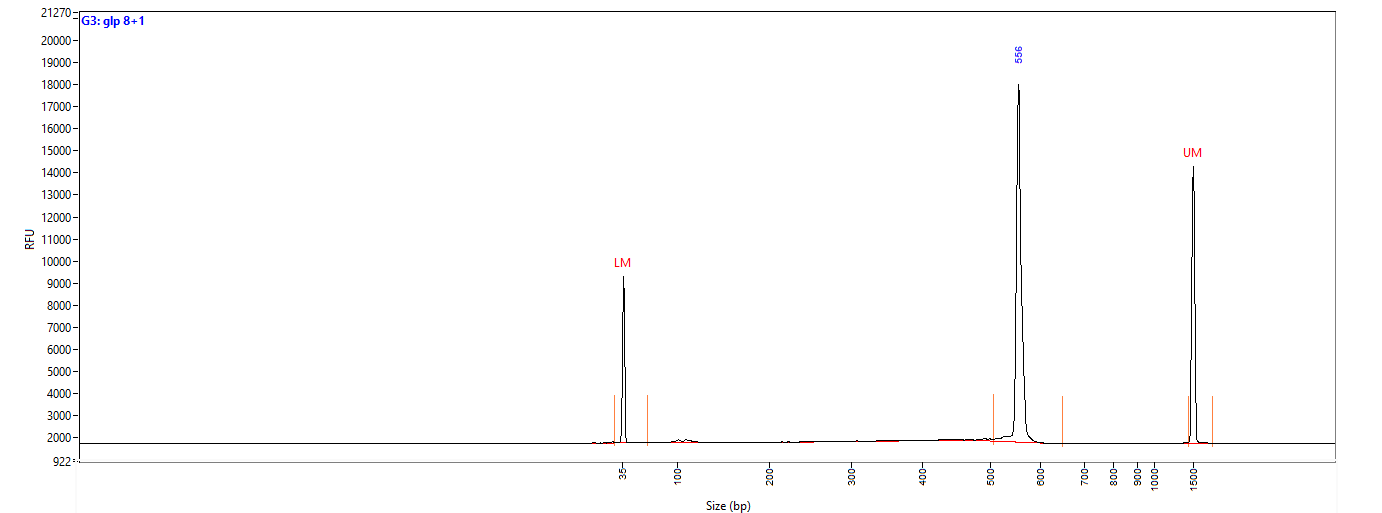

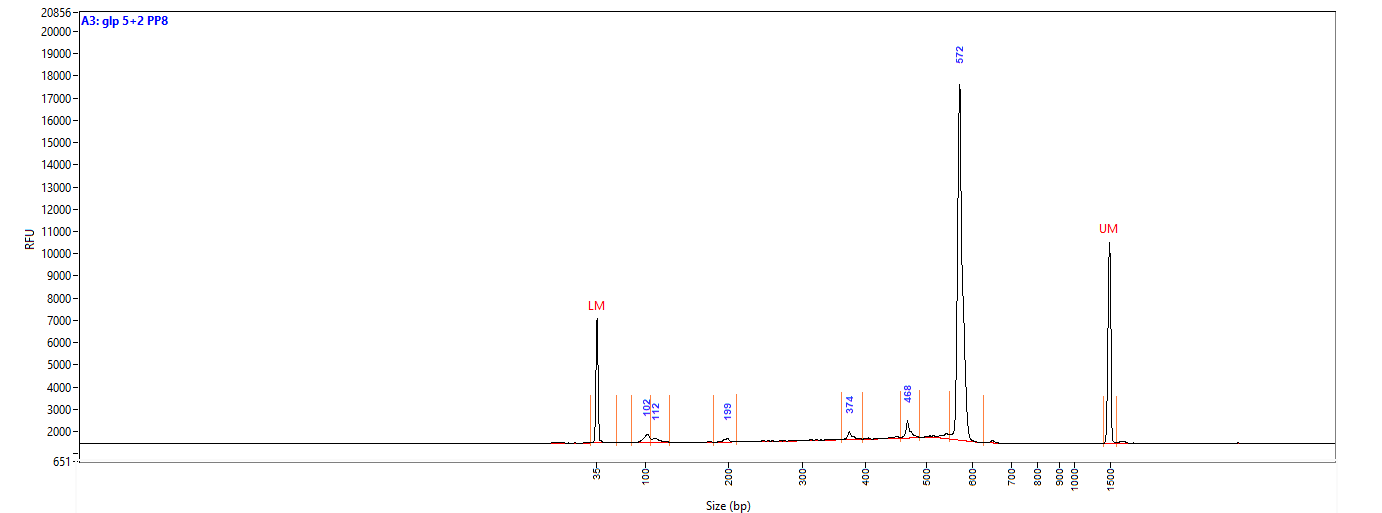

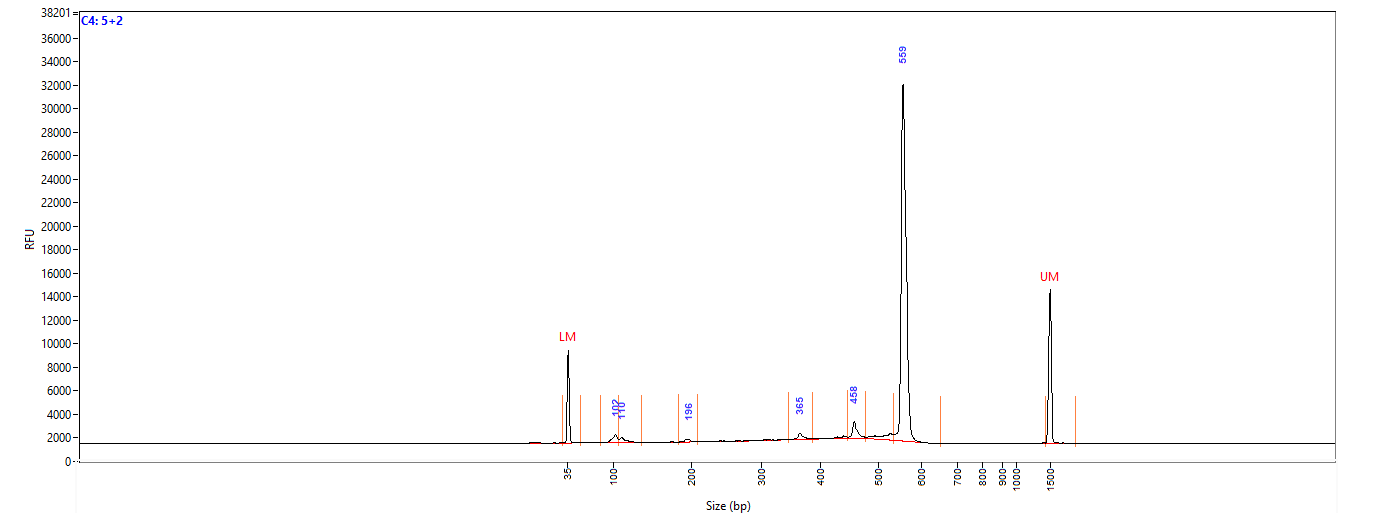


BCER10 (allele 19) vs BCER5 (allele 3): 2 SNP expected peaks 25, 100, 110, 198, 365, 458, 560 bp


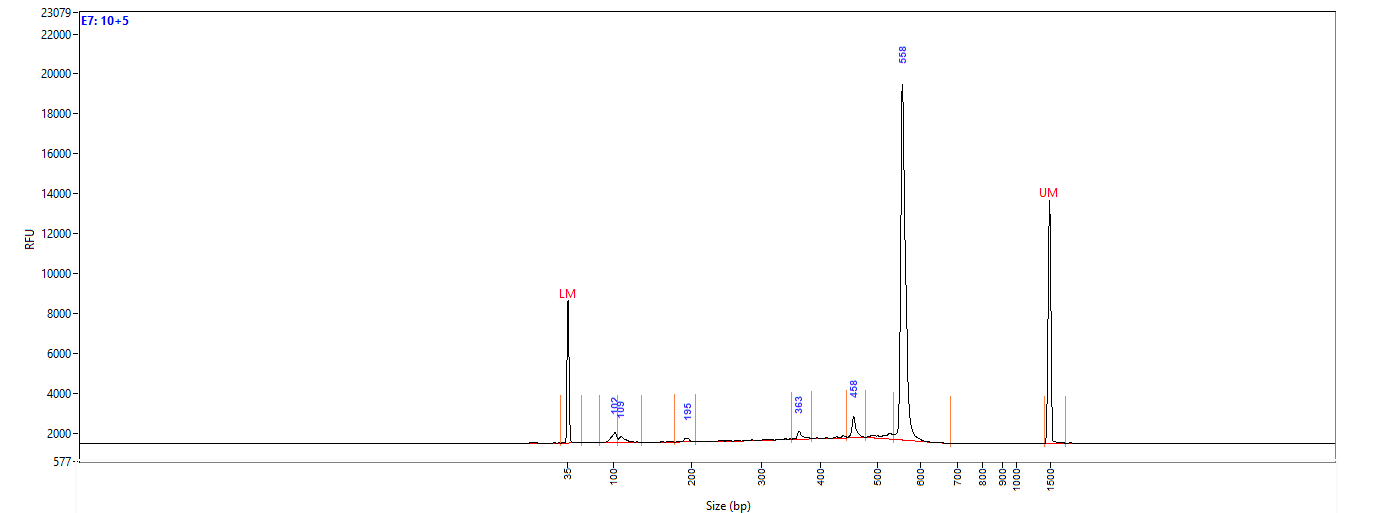


BCER2 (allele 19) vs BCER13 (allele 3): 2 SNP expected peaks 25, 100, 110, 198, 365, 458, 560 bp


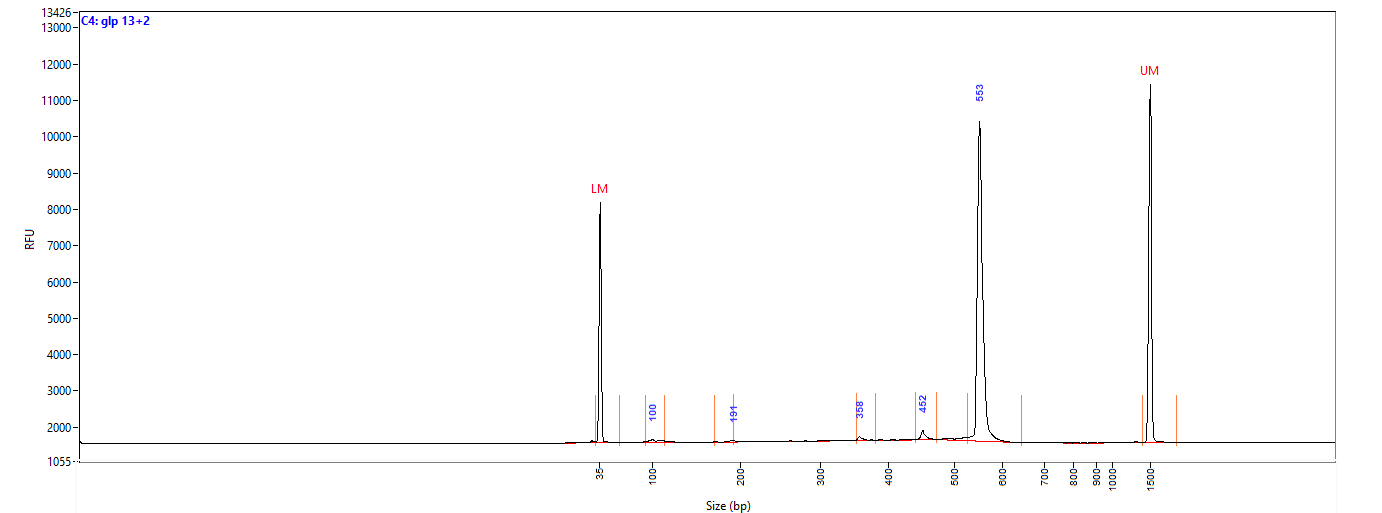


BCER12 (allele 67) vs BCER13 (allele 3): 6 SNP


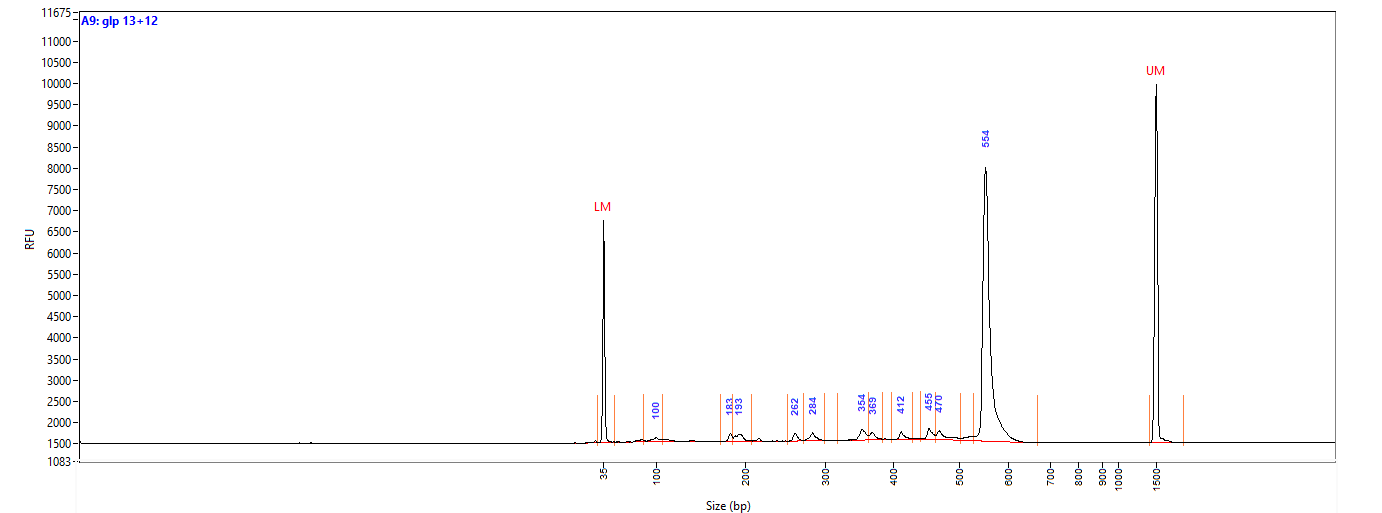


BCER12 (allele 67) vs BCER5 (allele 3): 6 SNP


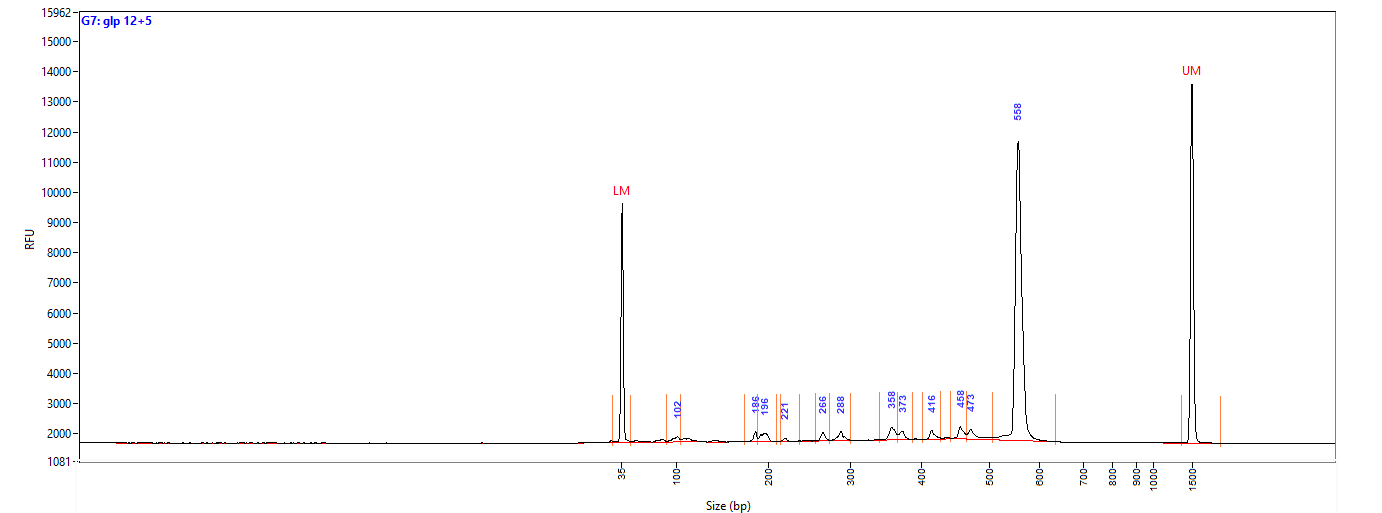


BCER10 (allele 19) vs BCER4 (allele 51): 1 SNP


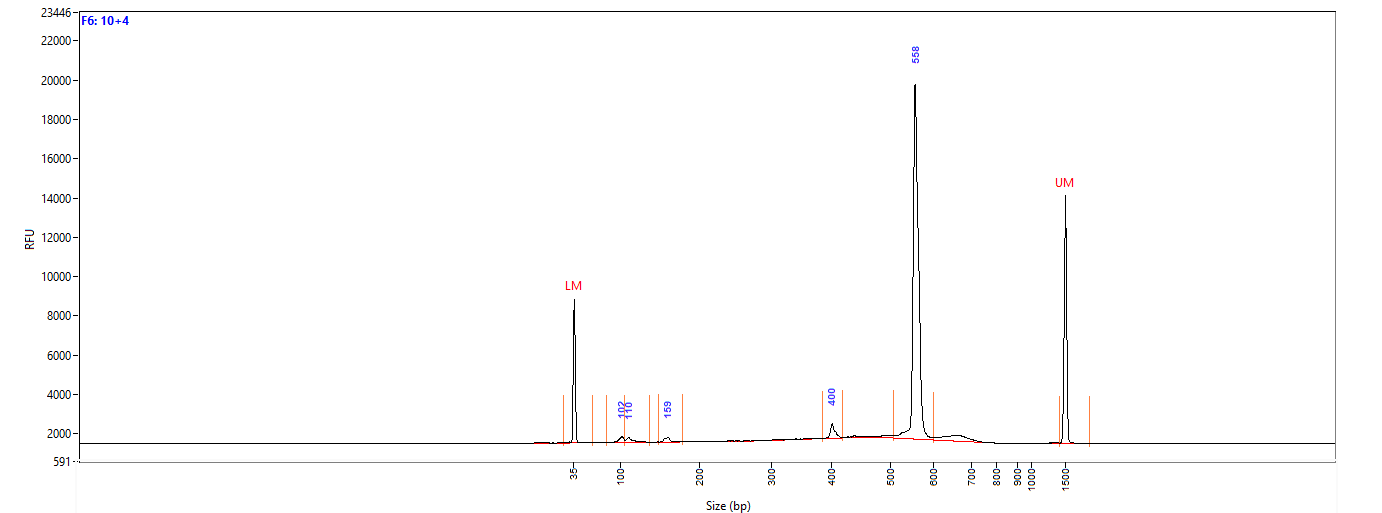


BCER2 (allele 19) vs BCER4 (allele 51): 1 SNP


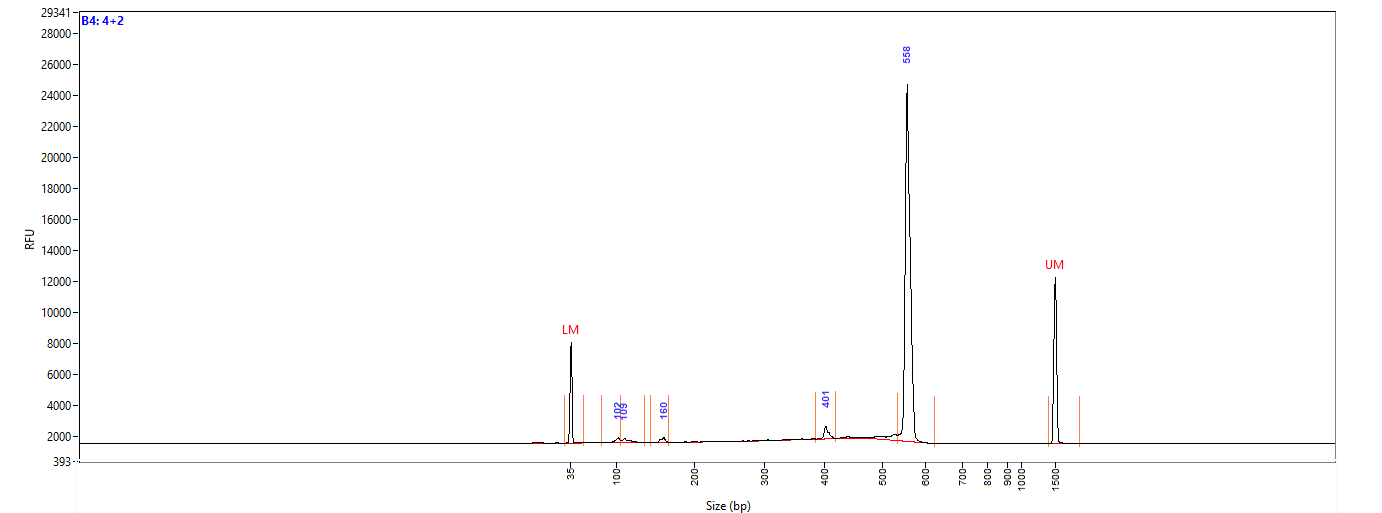


BCER5 (allele 3) vs BCER8 (allele 6): 5 SNP


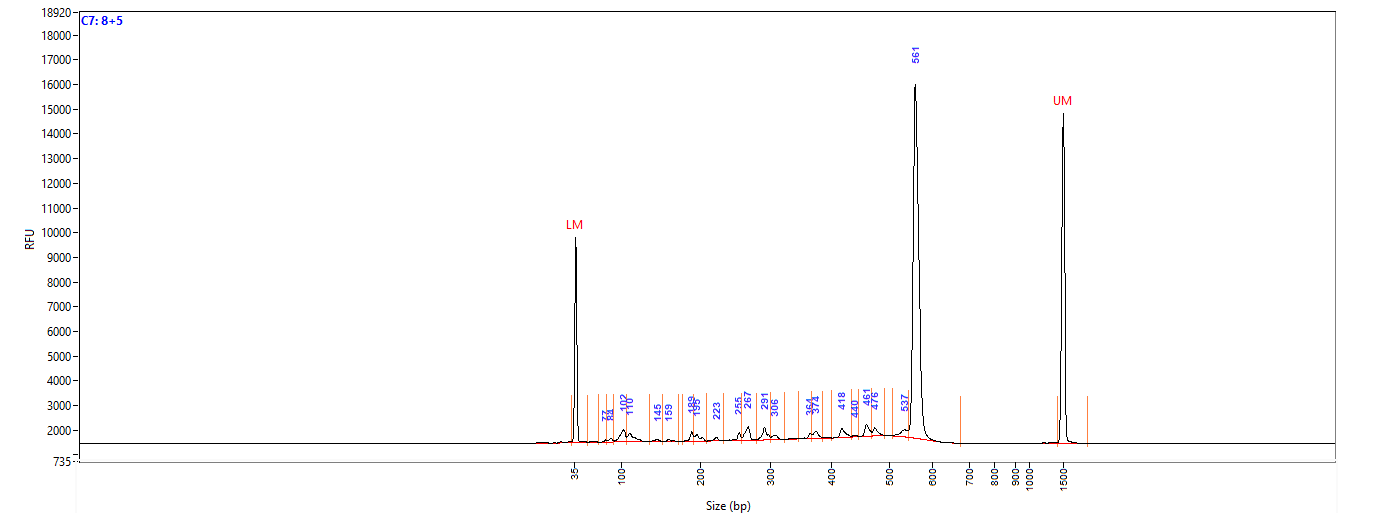


BCER5 (allele 3) vs BCER1 (allele 6): 5 SNP


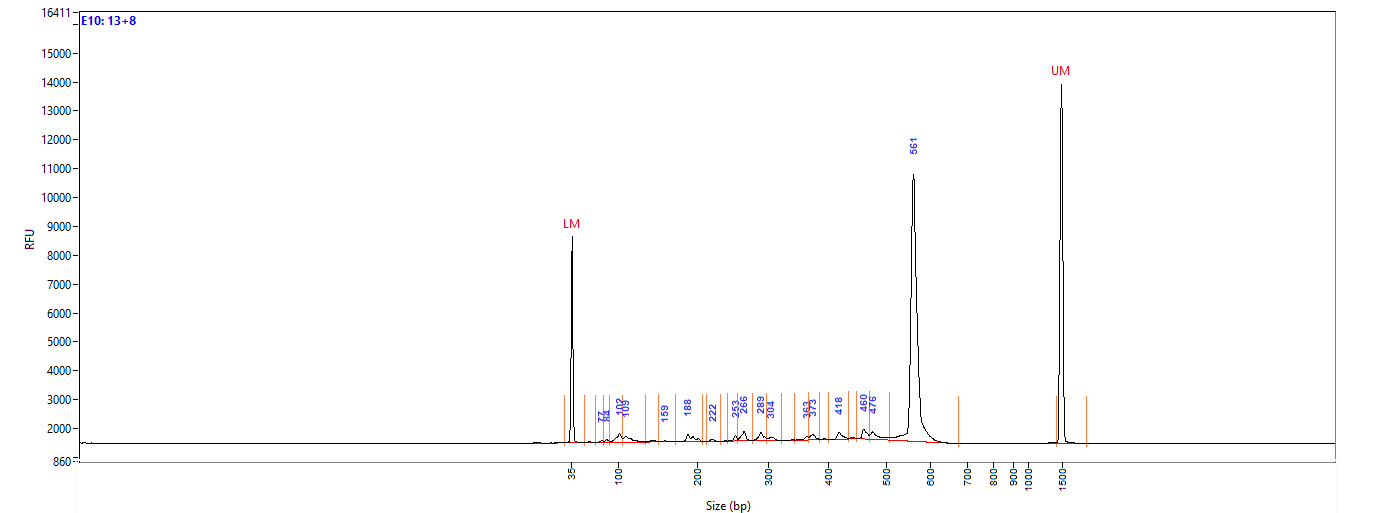


BCER13 (allele 3) vs BCER8 (allele 6): 5 SNP


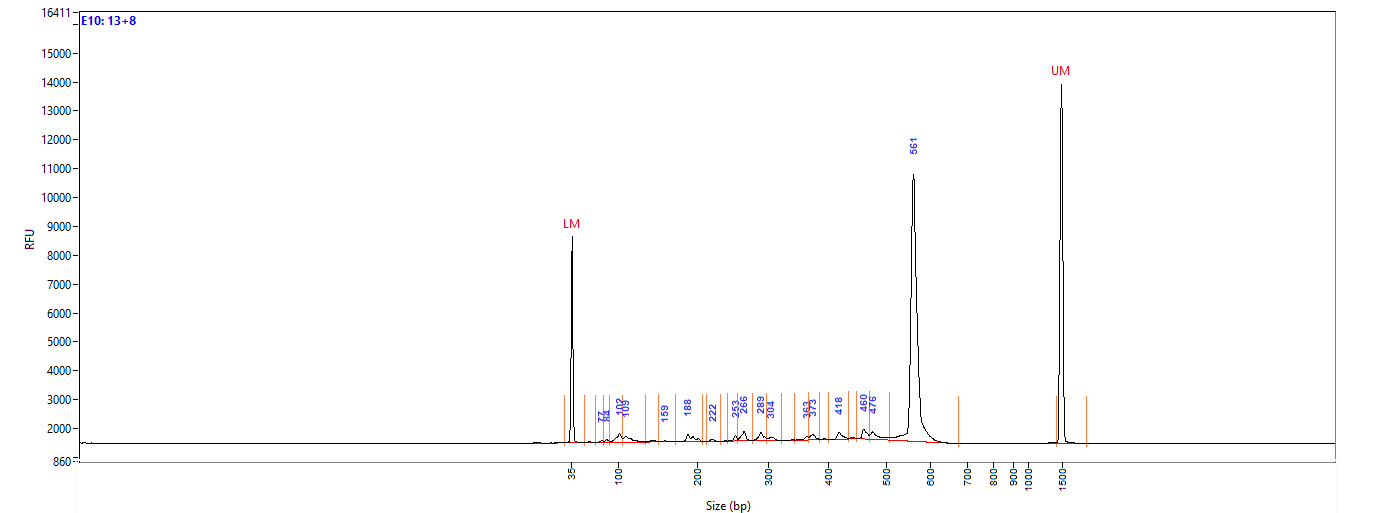


Gene: *gmk*

BCER3 (allele 8) vs BCER11 (allele 9): 1 SNP 173, 452, 620 bp


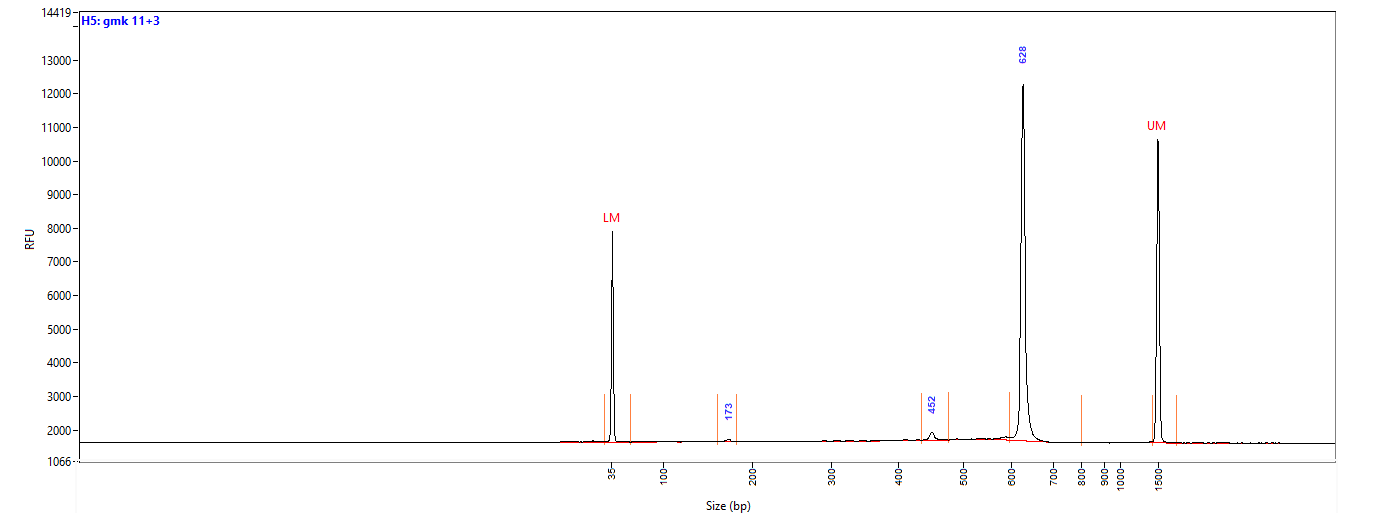


BCER6 (allele 8) vs BCER11 (allele 9): 1 SNP 173, 452, 620 bp


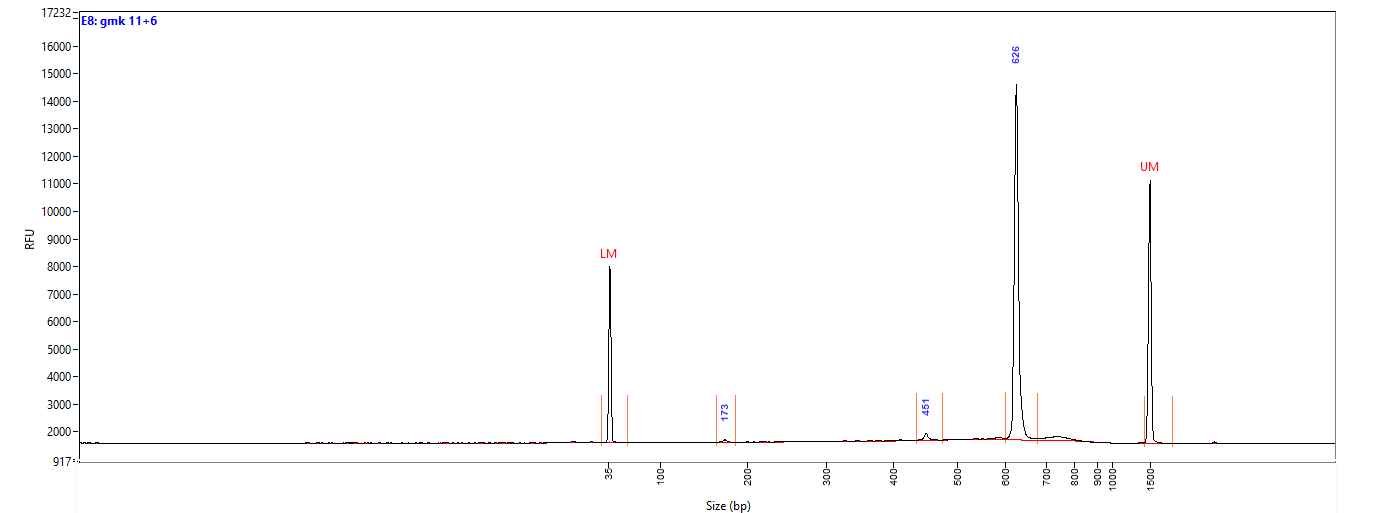


BCER7 (allele 8) vs BCER11 (allele 9): 1 SNP 173, 452, 620 bp


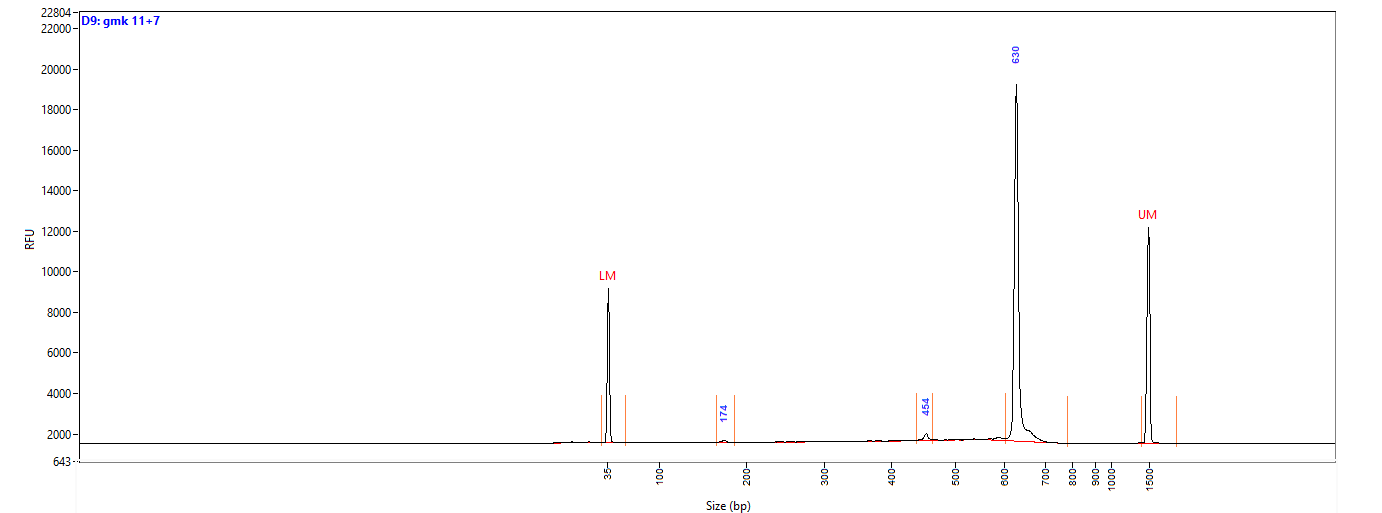


BCER9 (allele 8) vs BCER11 (allele 9): 1 SNP 173, 452, 620 bp


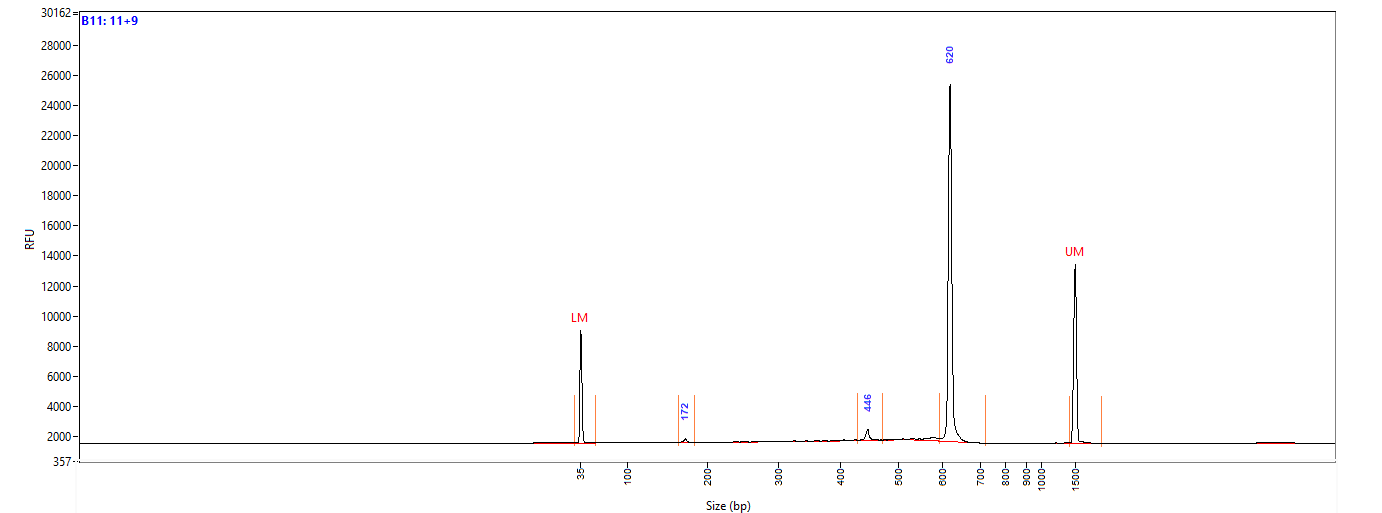


BCER14 (allele 7) vs BCER3 (allele 8): 3 SNP 93, 152, 153, 245, 354, 355, 467, 530, 620 bp


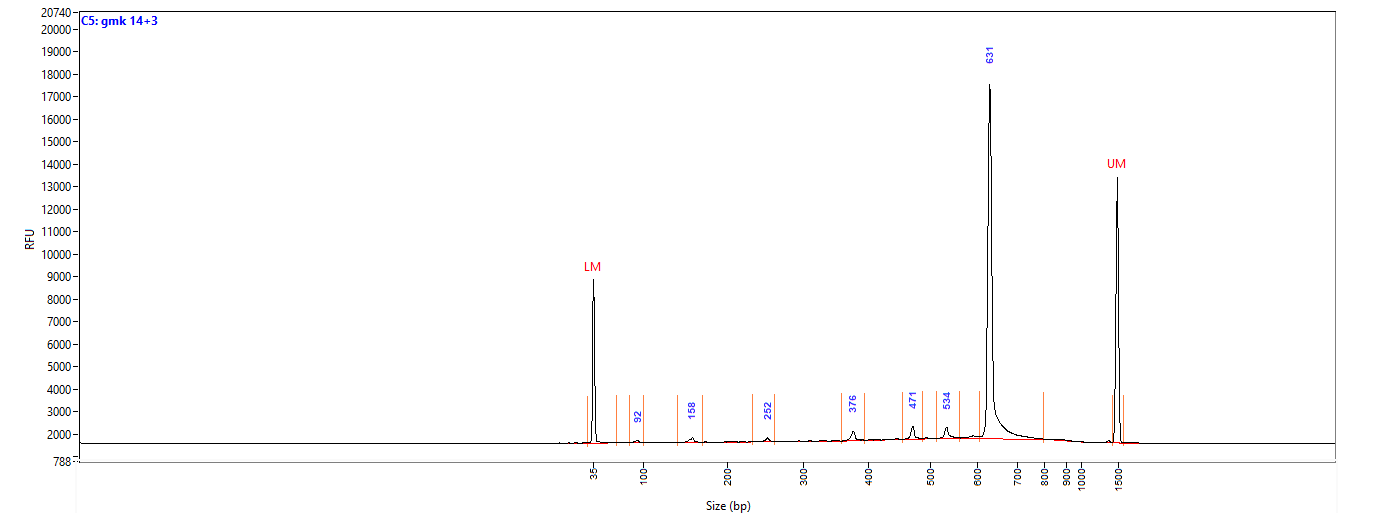


BCER14 (allele 7) vs BCER6 (allele 8): 3 SNP 93, 152, 153, 245, 354, 355, 467, 530, 620 bp


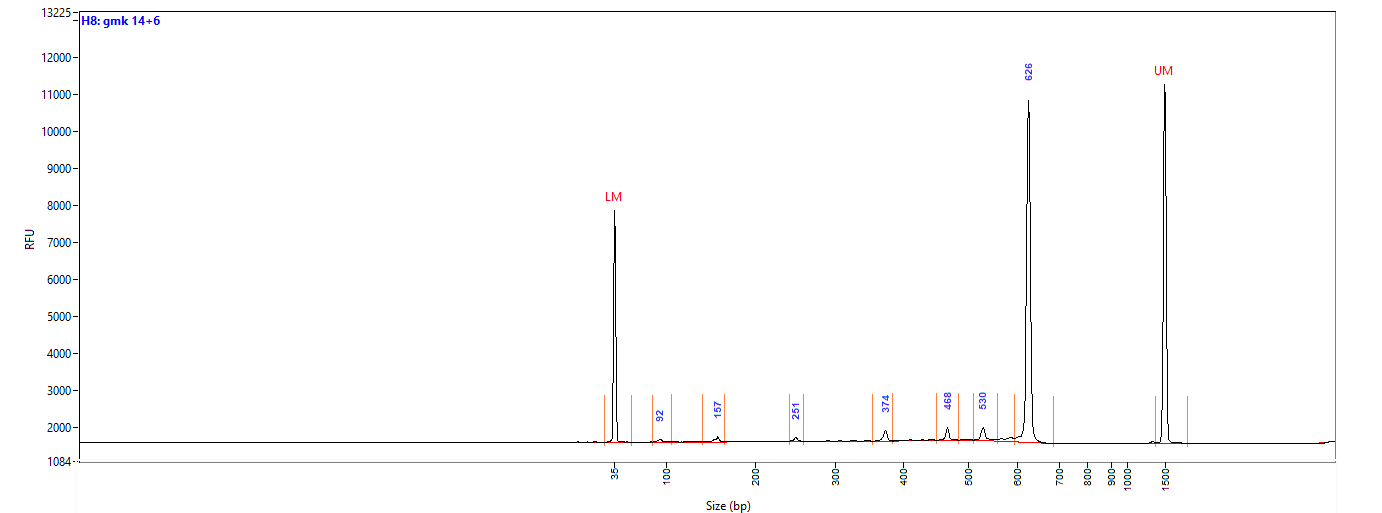


BCER14 (allele 7) vs BCER7 (allele 8): 3 SNP 93, 152, 153, 245, 354, 355, 467, 530, 620 bp


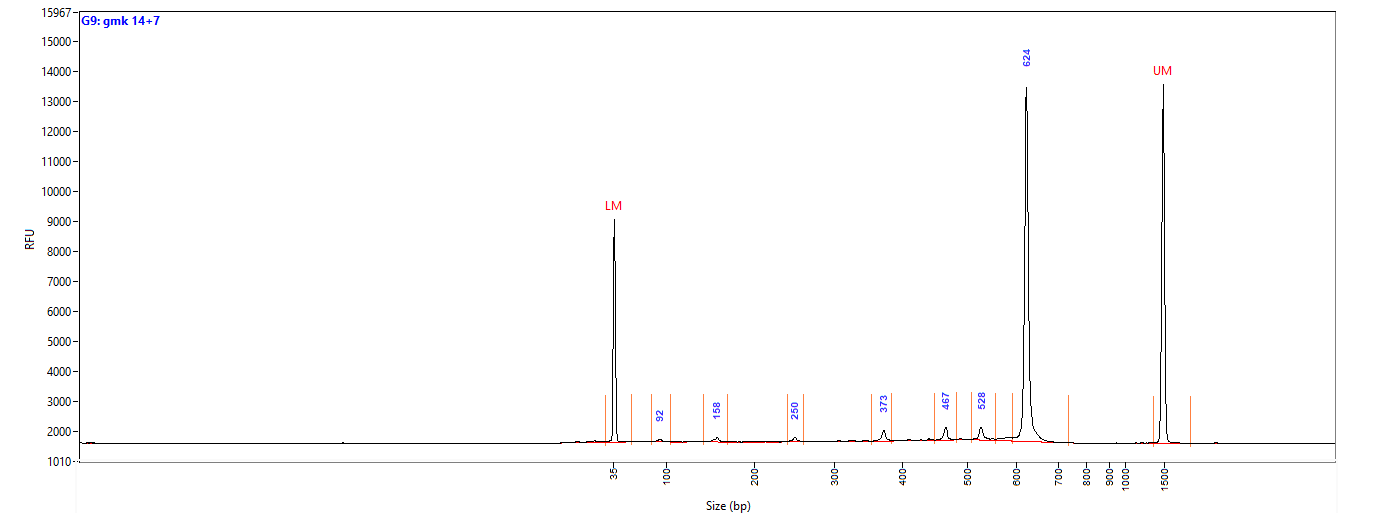


BCER14 (allele 7) vs BCER9 (allele 8): 3 SNP 93, 152, 153, 245, 354, 355, 467, 530, 620 bp


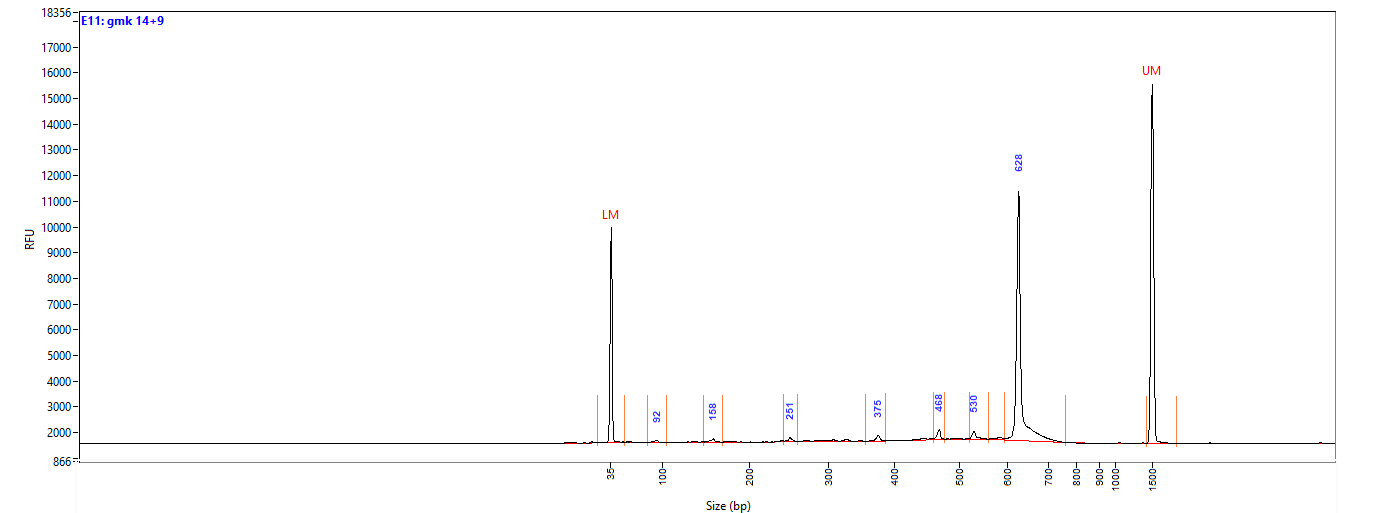


BCER9 (allele 8) vs BCER3 (allele 8): 0 SNP


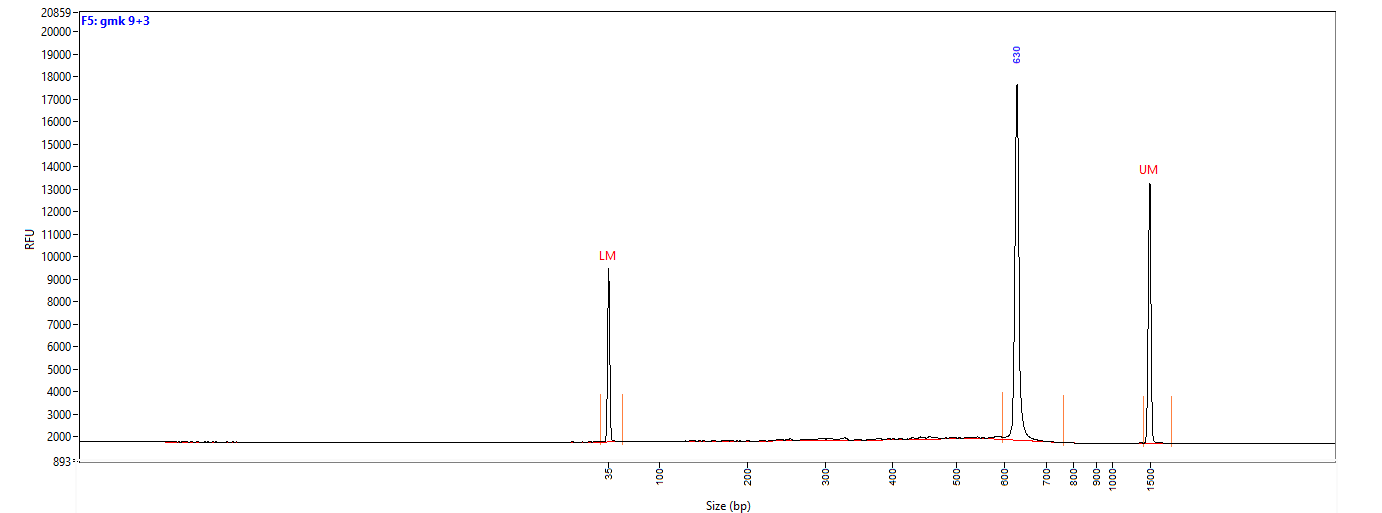


BCER9 (allele 8) vs BCER6 (allele 8): 0 SNP


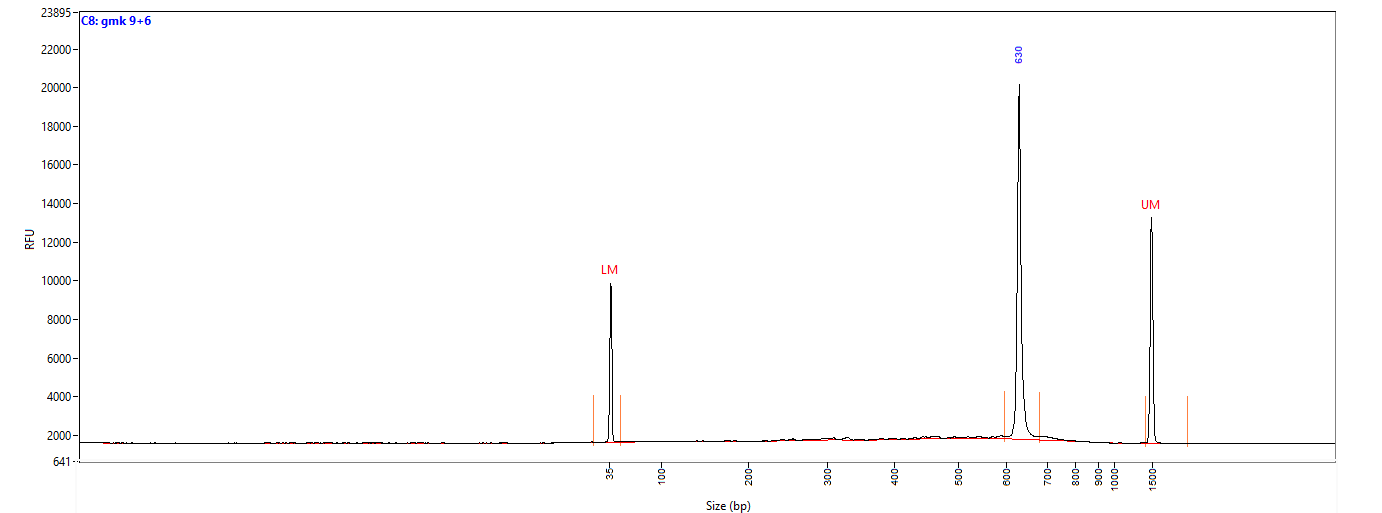


BCER9 (allele 8) vs BCER7 (allele 8): 0 SNP


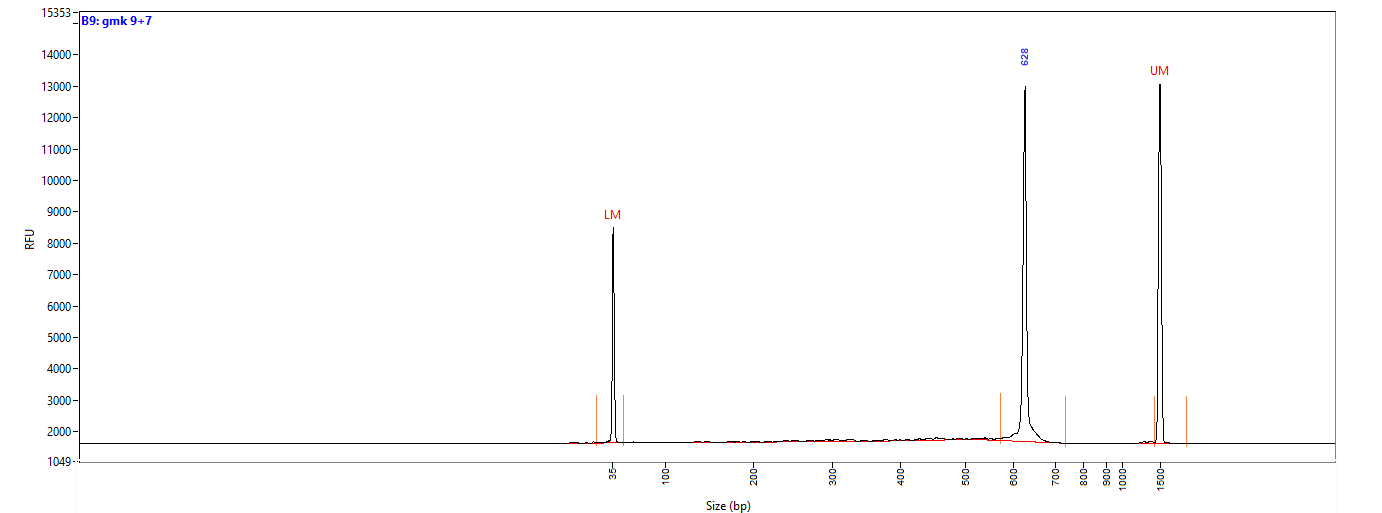


BCER3 (allele 8) vs BCER6 (allele 8): 0 SNP


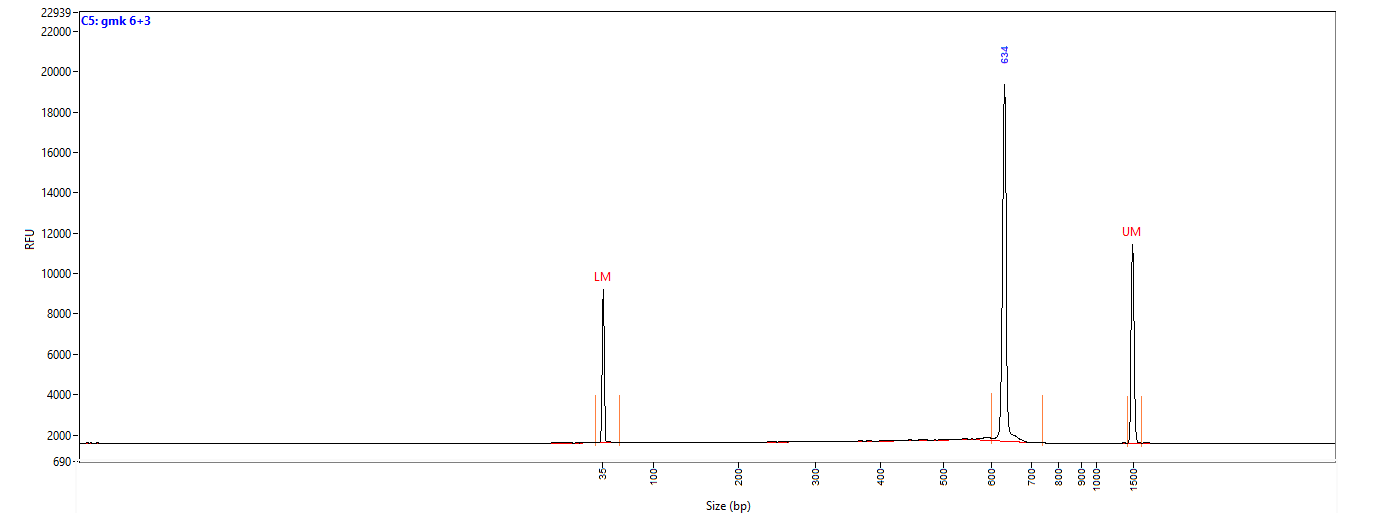


BCER3 (allele 8) vs BCER7 (allele 8): 0 SNP


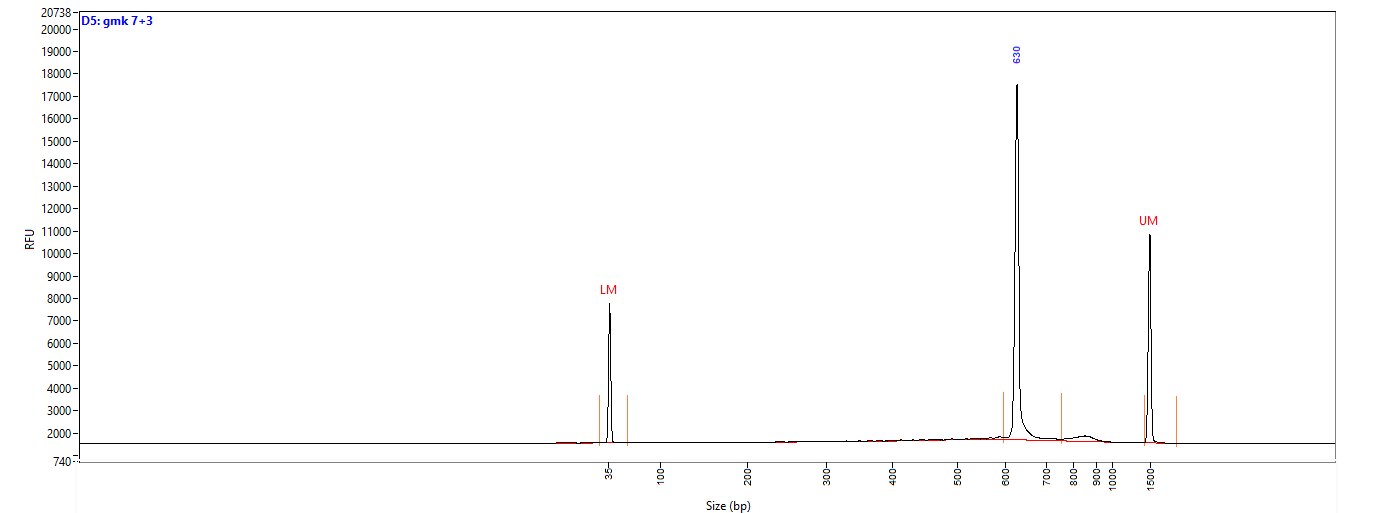


BCER6 (allele 8) vs BCER7 (allele 8): 0 SNP


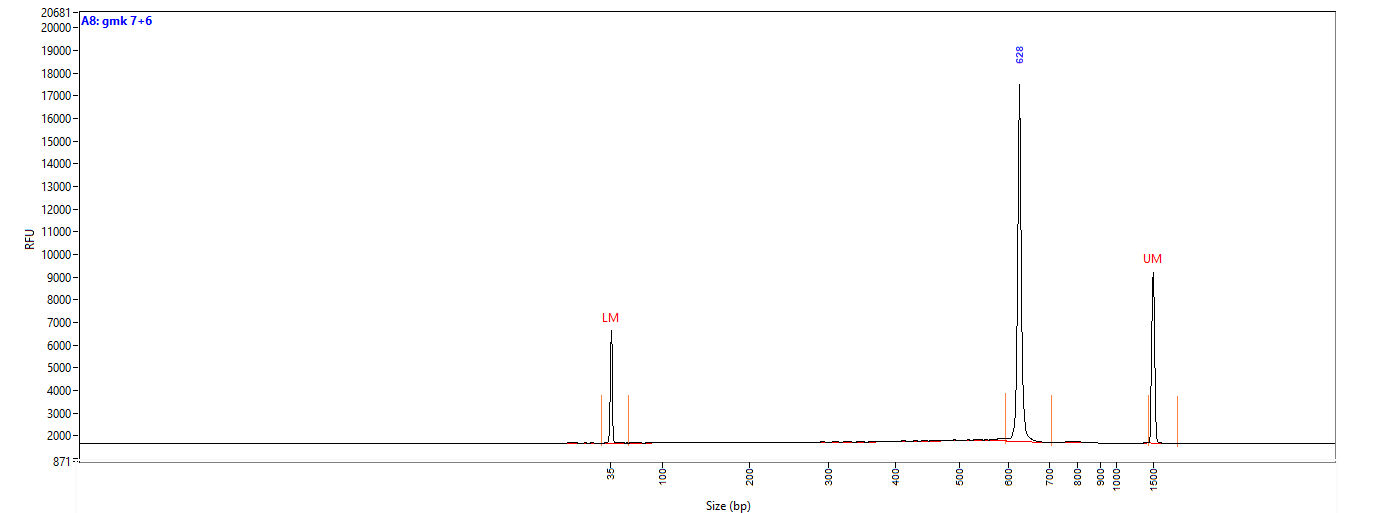


Gene: *tpi*

Strain BCER2 (allele 2) vs strain BCER1 (allele 3): 1 SNP: expected peaks 225, 373, 578 bp


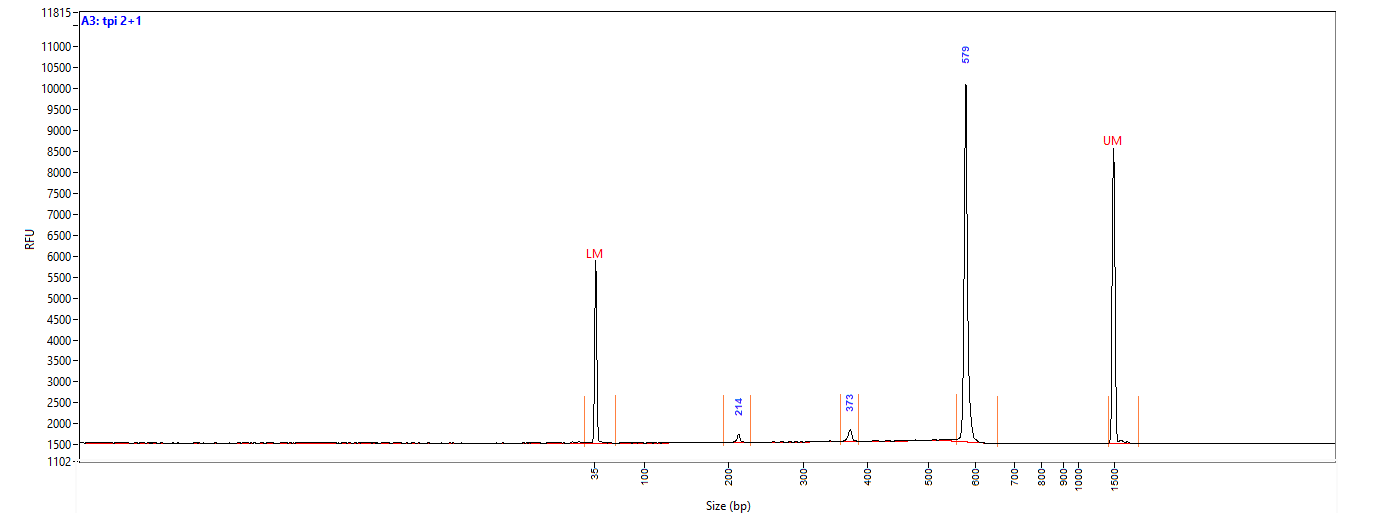


Strain BCER2 (allele 2) vs strain BCER8 (allele 3) vs: 1 SNP: expected peaks 225, 373, 578 bp


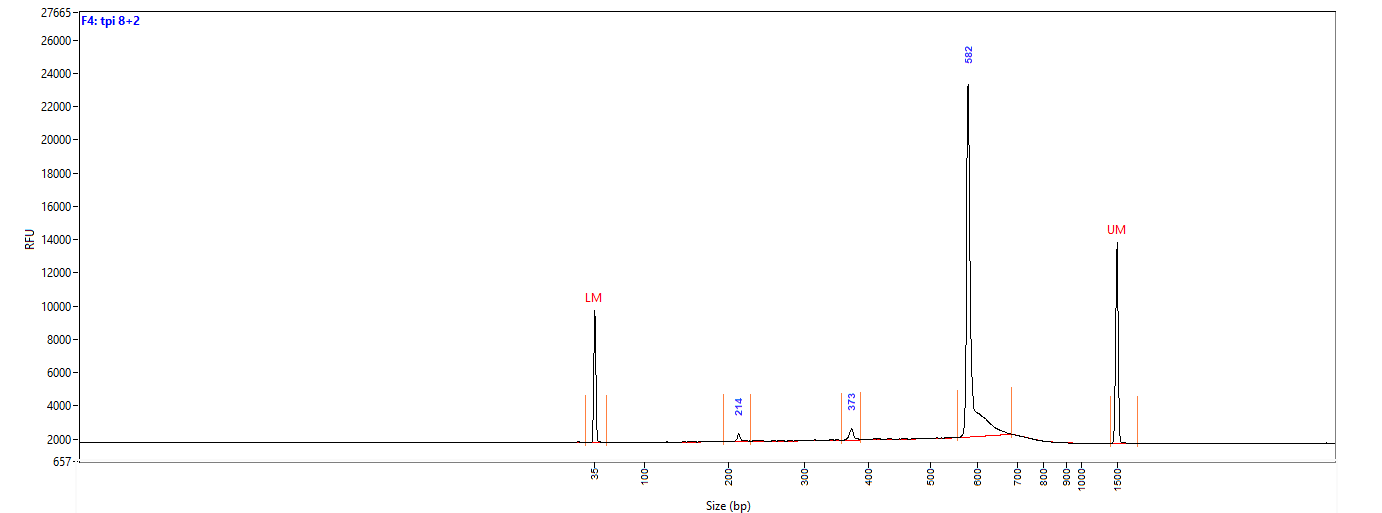


Strain BCER4 (allele 2) vs strain BCER1 (allele 3): 1 SNP: expected peaks 225, 373, 578 bp


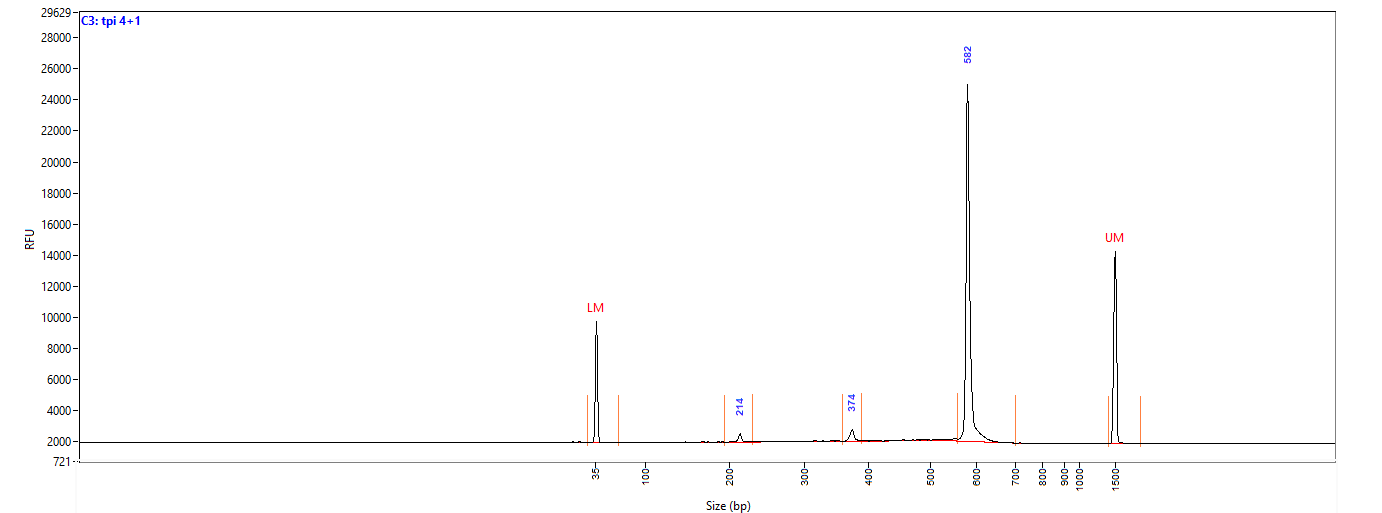


Strain BCER4 (allele 2) vs strain BCER8 (allele 3): 1 SNP: expected peaks 225, 373, 578 bp


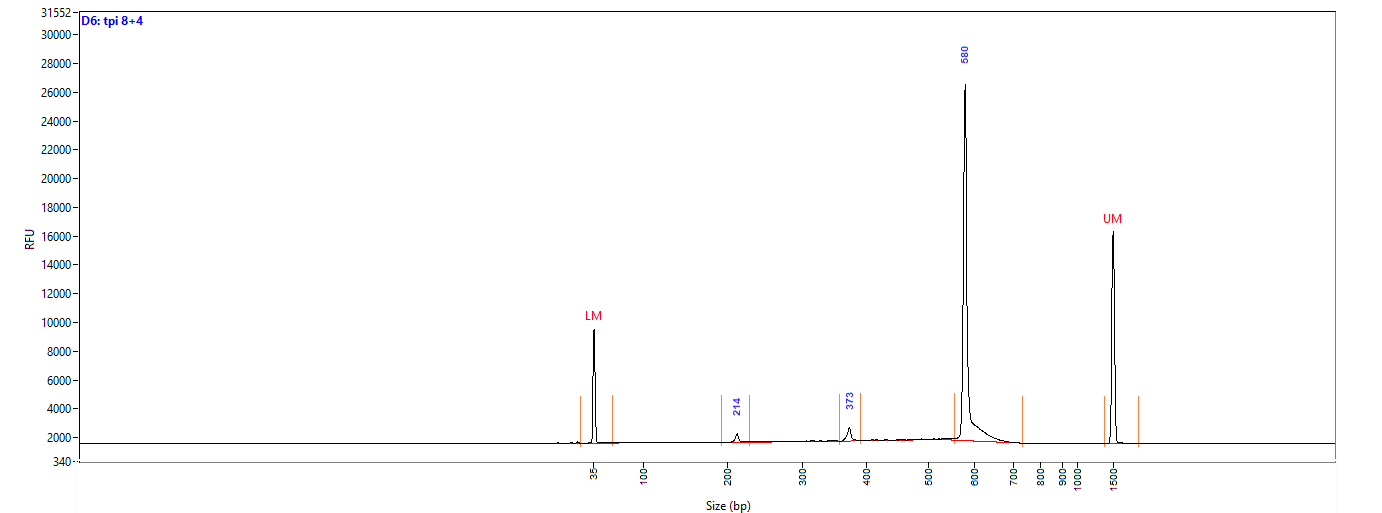


Strain BCER4 (allele 2) vs strain BCER2 (allele 2): 0 SNP: expected peak 578 bp


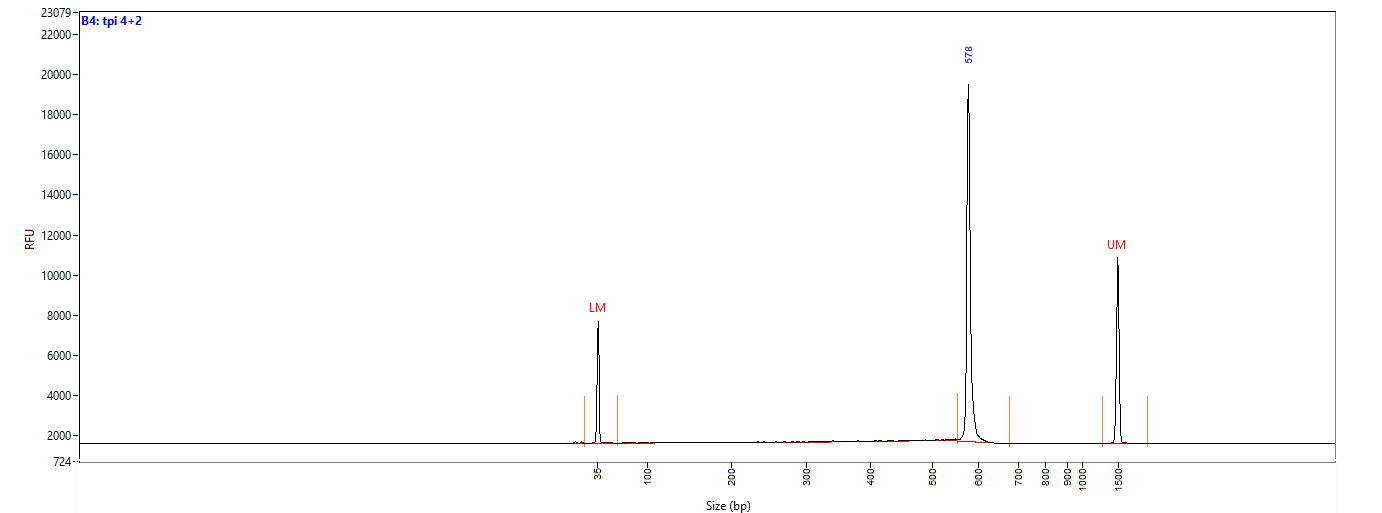


Strain BCER1 (allele 3) vs strain BCER8 (allele 3): 0 SNP: expected peak 578 bp


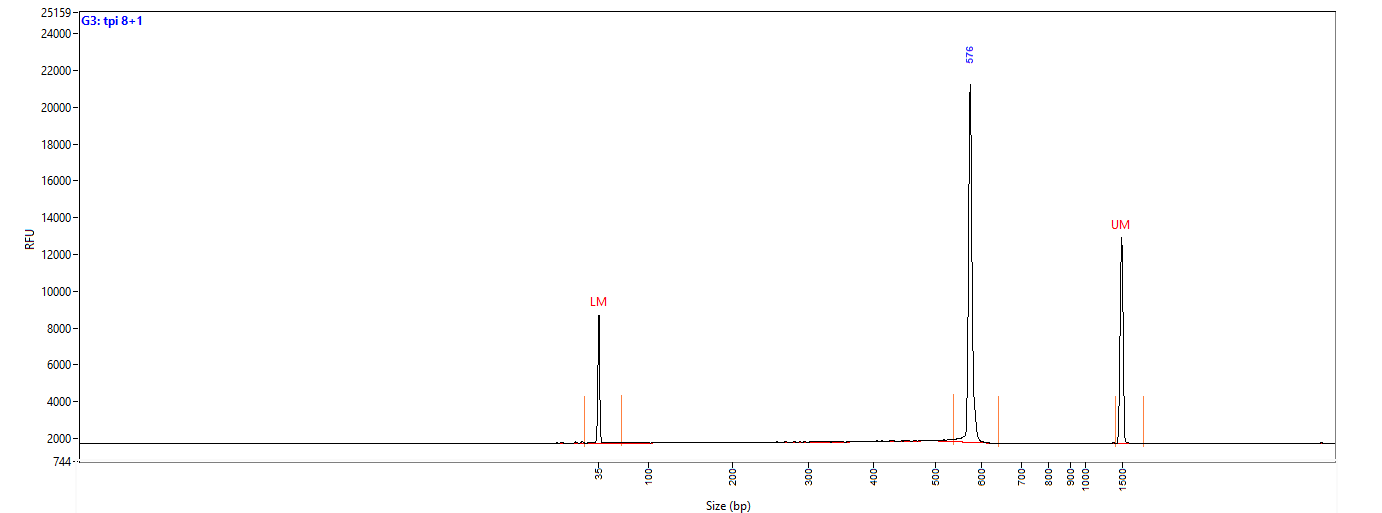


Strain BCER10 (allele 183) vs strain BCER7 (allele 7): 7 SNP


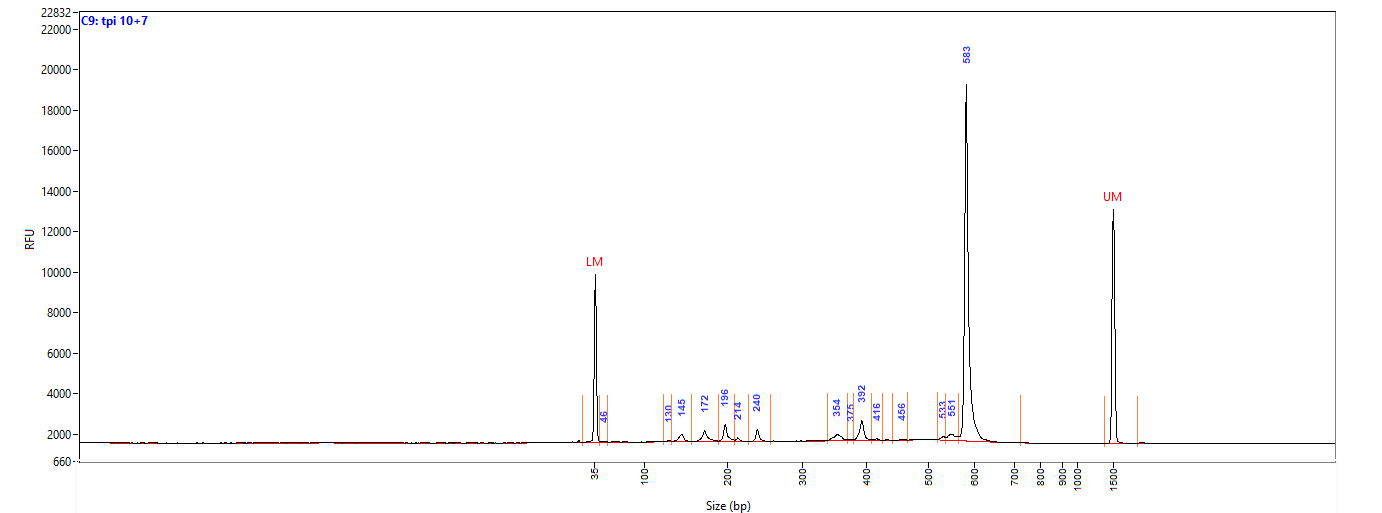


Strain BCER10 (allele 183) vs strain BCER11 (allele 7): 7 SNP


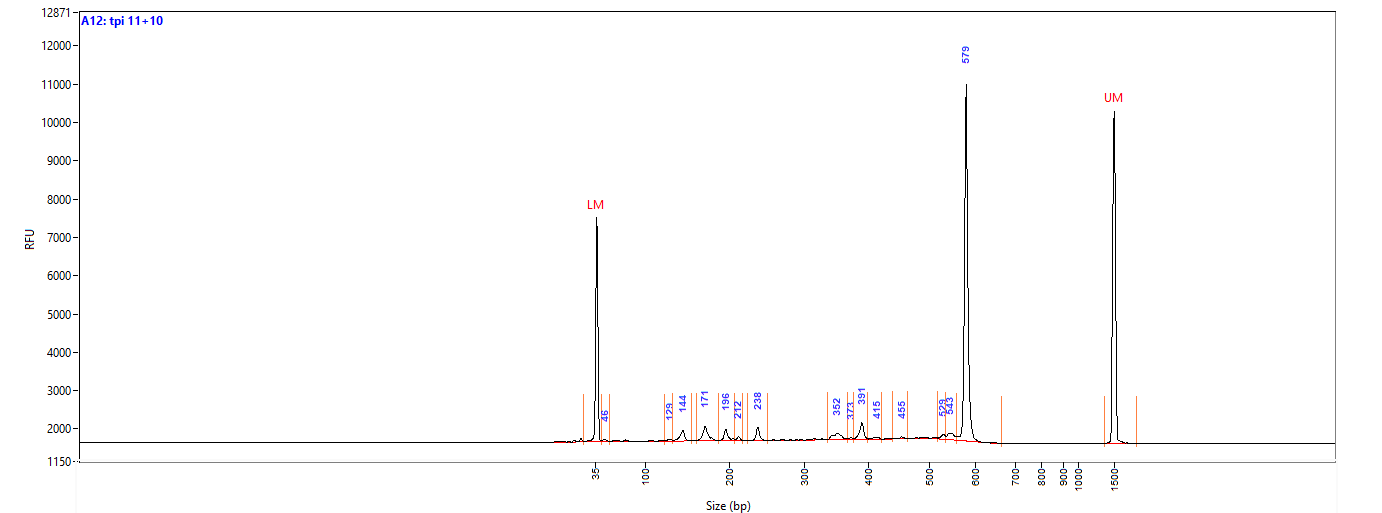


Strain BCER7 (allele 7) vs strain BCER11 (allele 7): 0 SNP


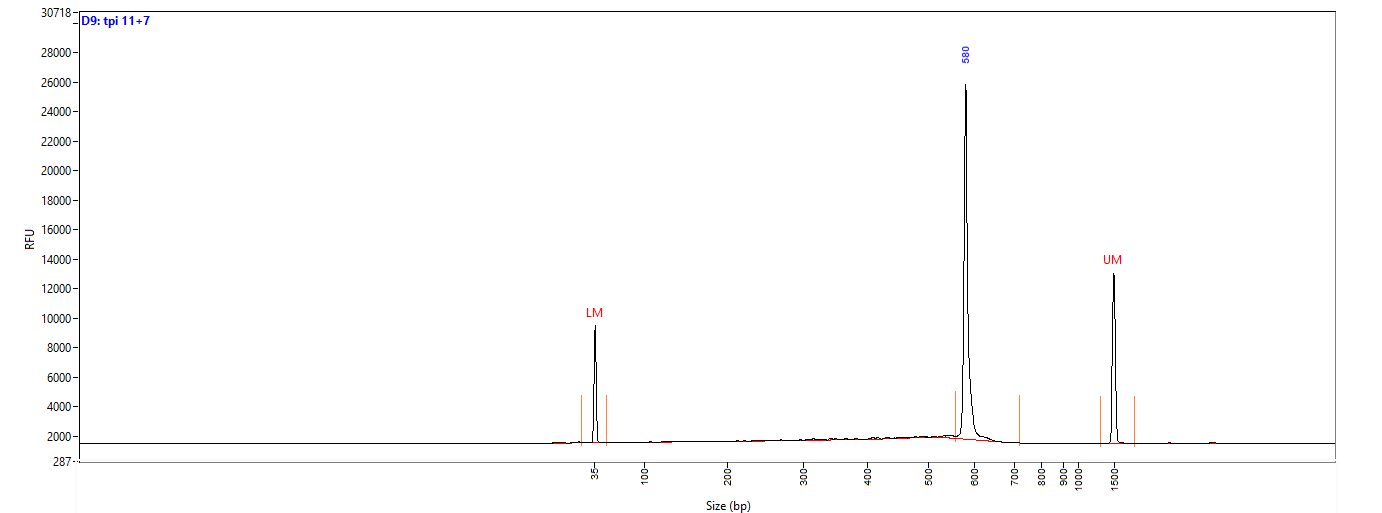


Gene: *ilvD*

BCER6 (allele 8) vs BCER3 (allele 9): 1 SNP expected peaks 282, 484, 576 bp


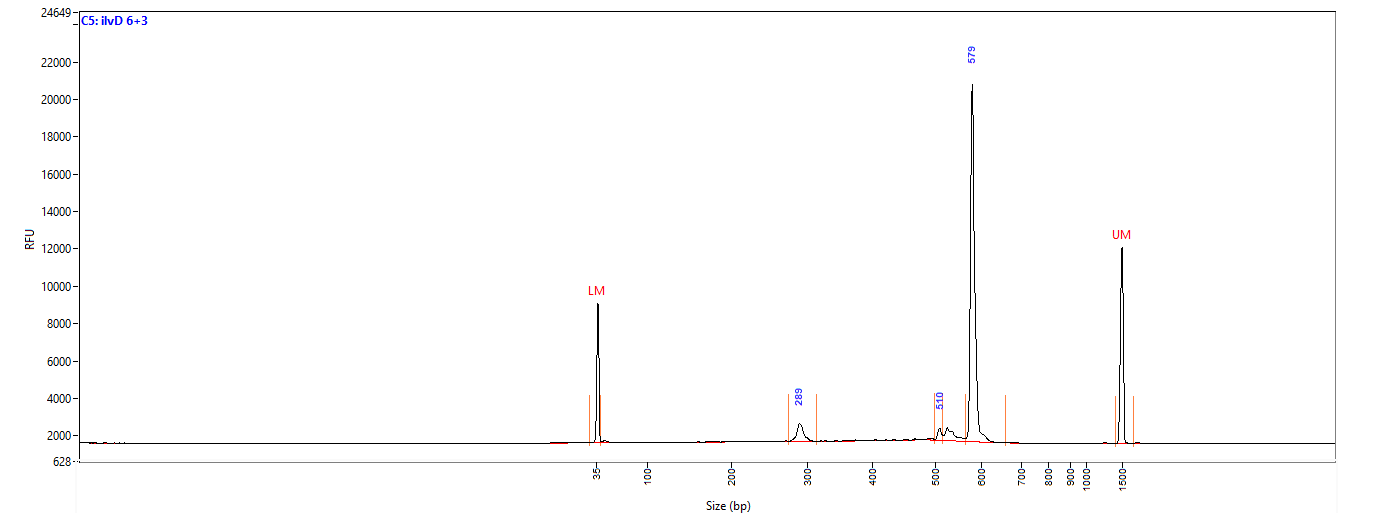


BCER6 (allele 8) vs BCER9 (allele 9): 1 SNP expected peaks 282, 484, 576 bp


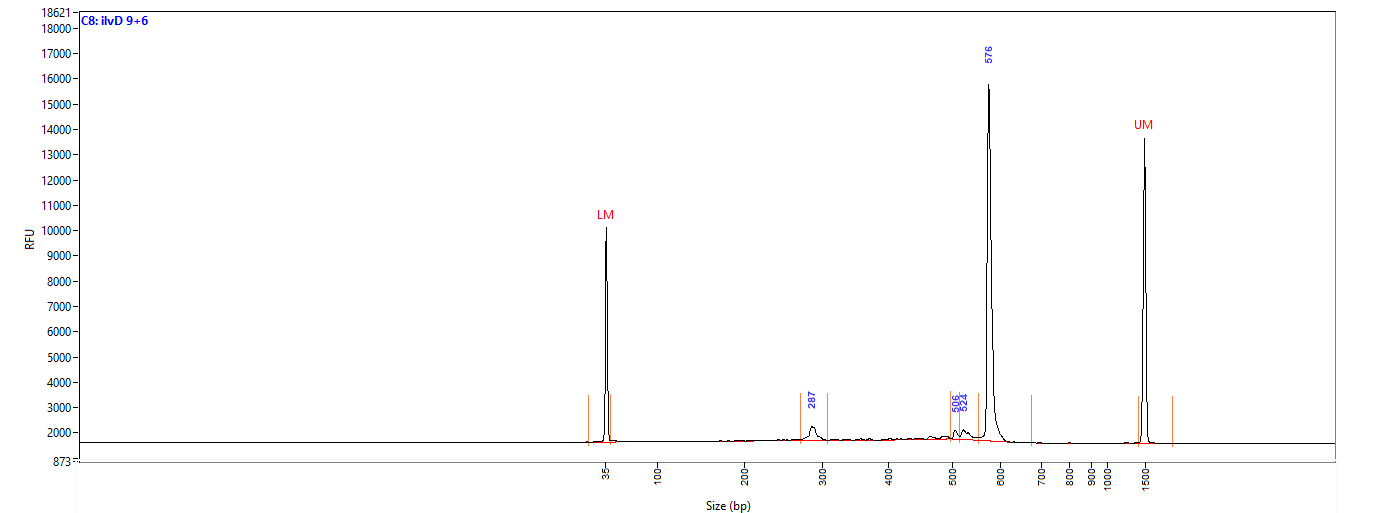


BCER3 (allele 9) vs BCER9 (allele 9): 0 SNP


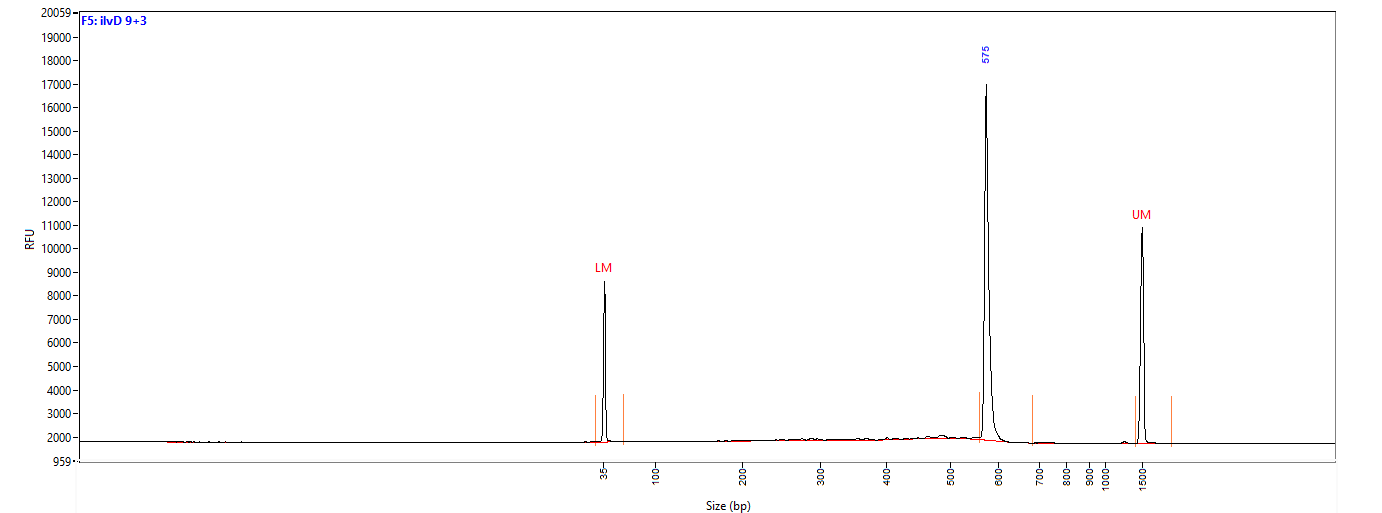


BCER11 (allele 14) vs BCER7 (allele 16): 2 SNP expected peaks 139, 191, 286, 310, 457, 576 bp

Gene: *Pta*


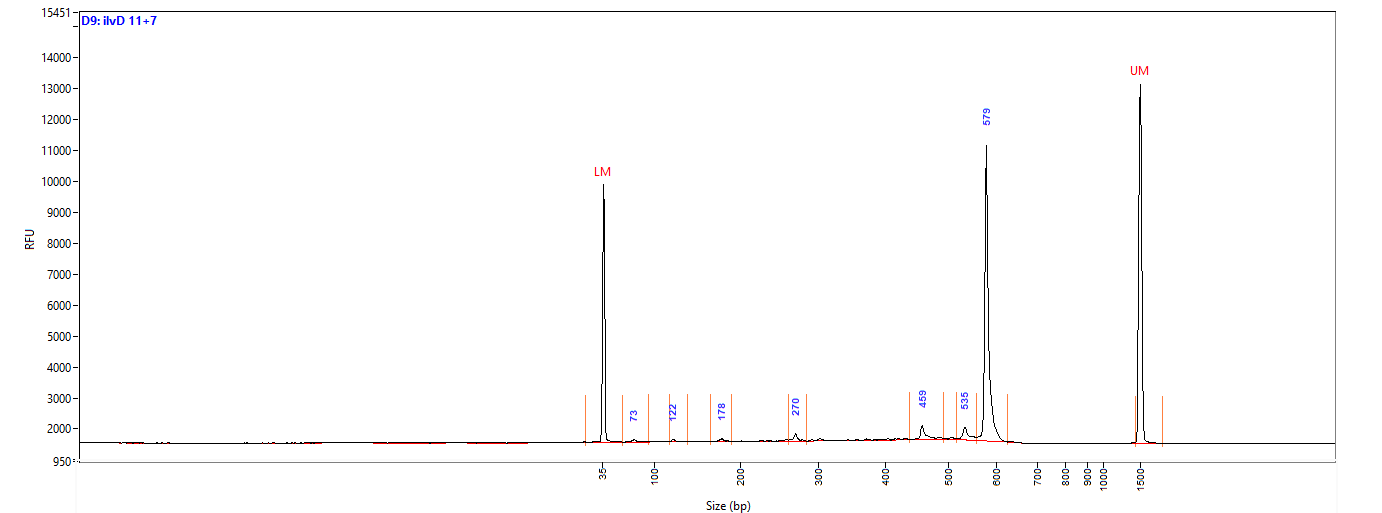


BCER1 (allele 4) vs BCER4 (allele 5): 1 SNP 170, 433, 605 bp


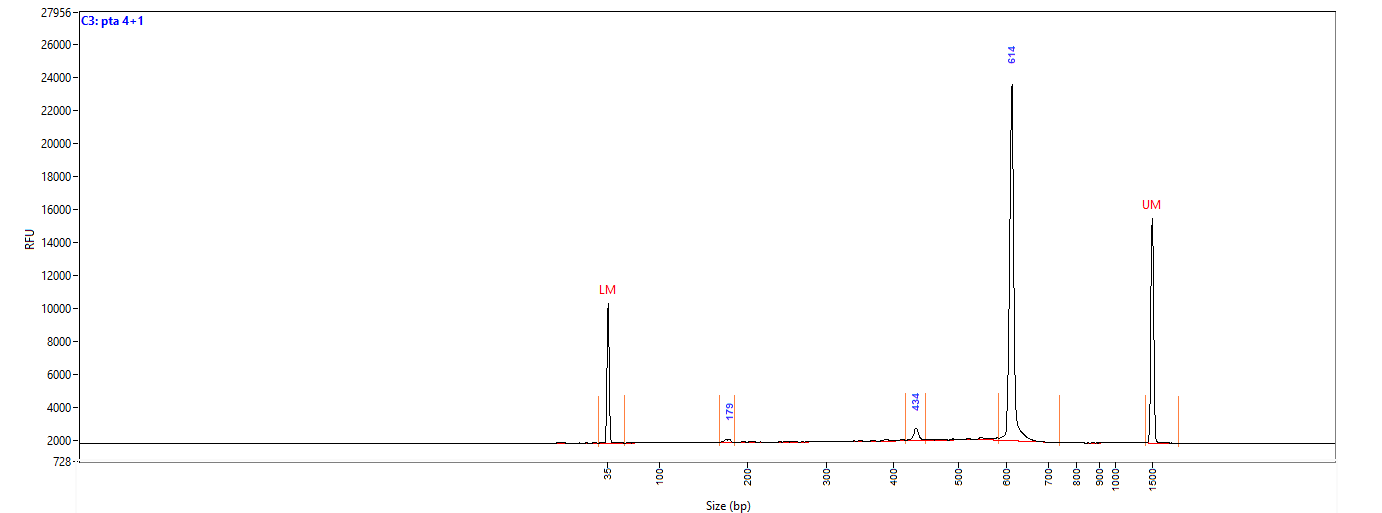


BCER1 (allele 4) vs BCER 5 (allele 5): 1 SNP 170, 433, 605 bp


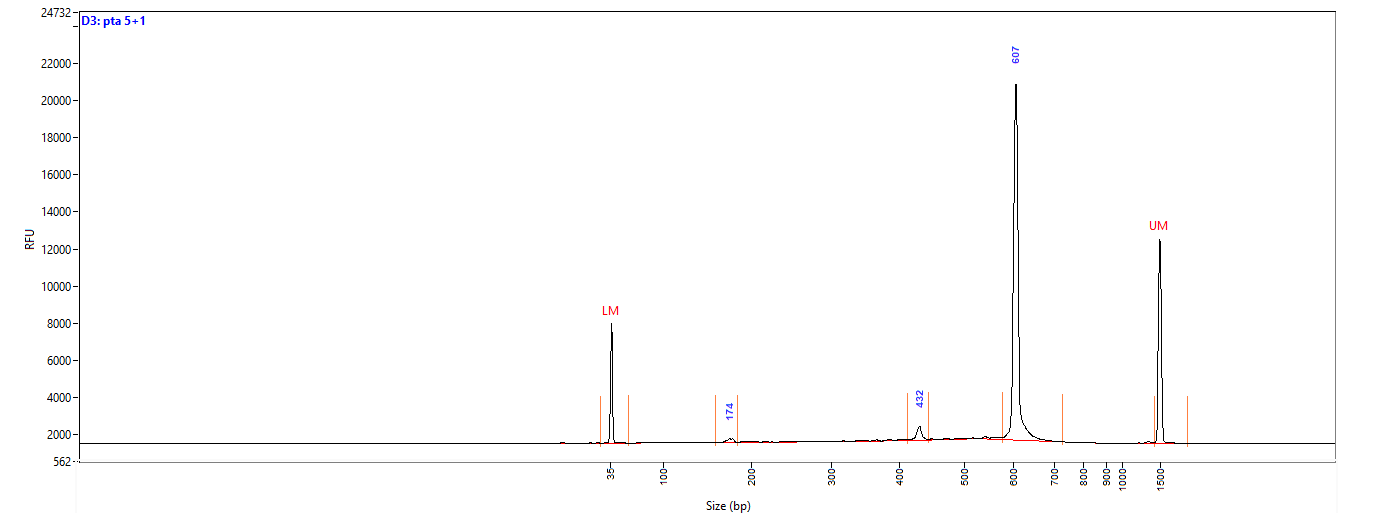


BCER1 (allele 4) vs BCER10 (allele 5): 1 SNP 170, 433, 605 bp


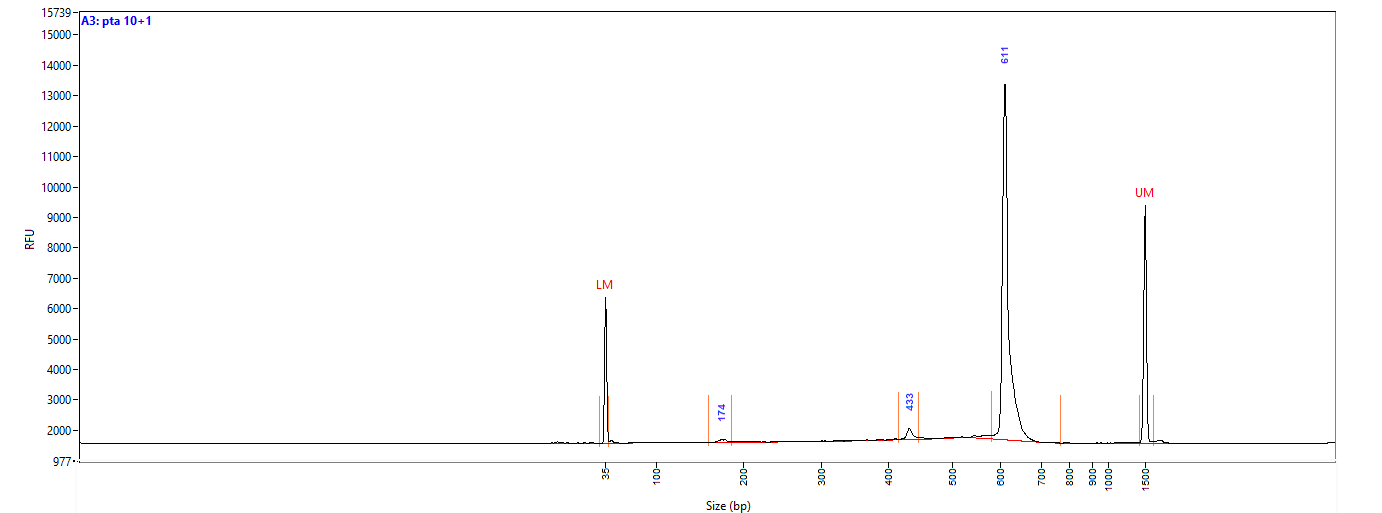


BCER1 (allele 4) vs BCER12 (allele 5): 1 SNP 170, 433, 605 bp


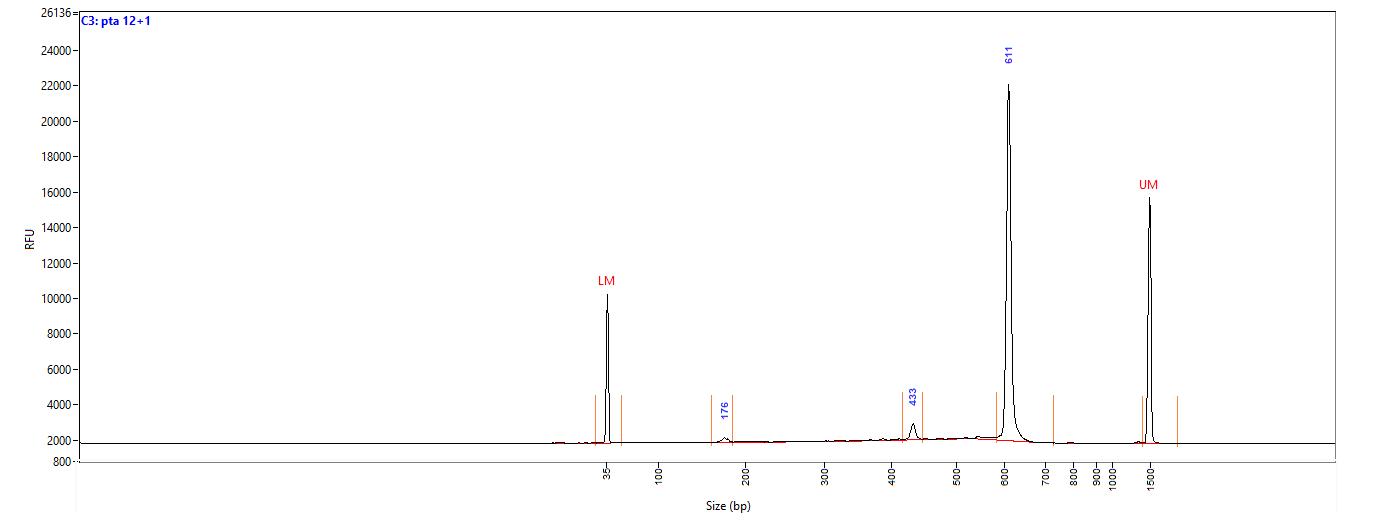


BCER1 (allele 4) vs BCER13 (allele 5): 1 SNP 170, 433, 605 bp


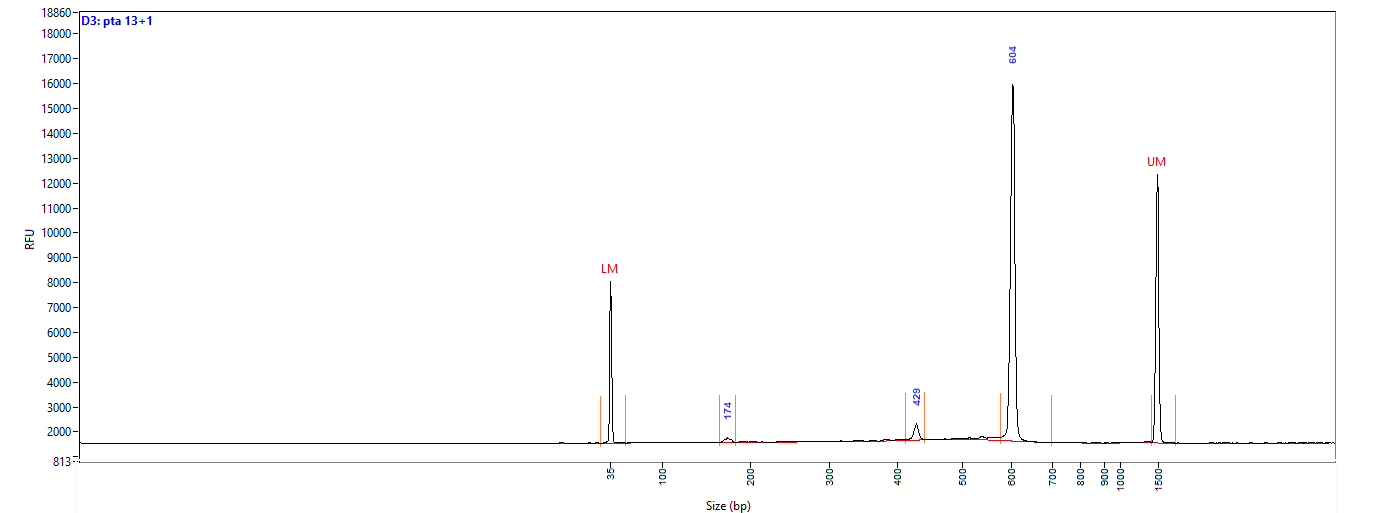


BCER1 (allele 4) vs BCER8 (allele 4): 0 SNP


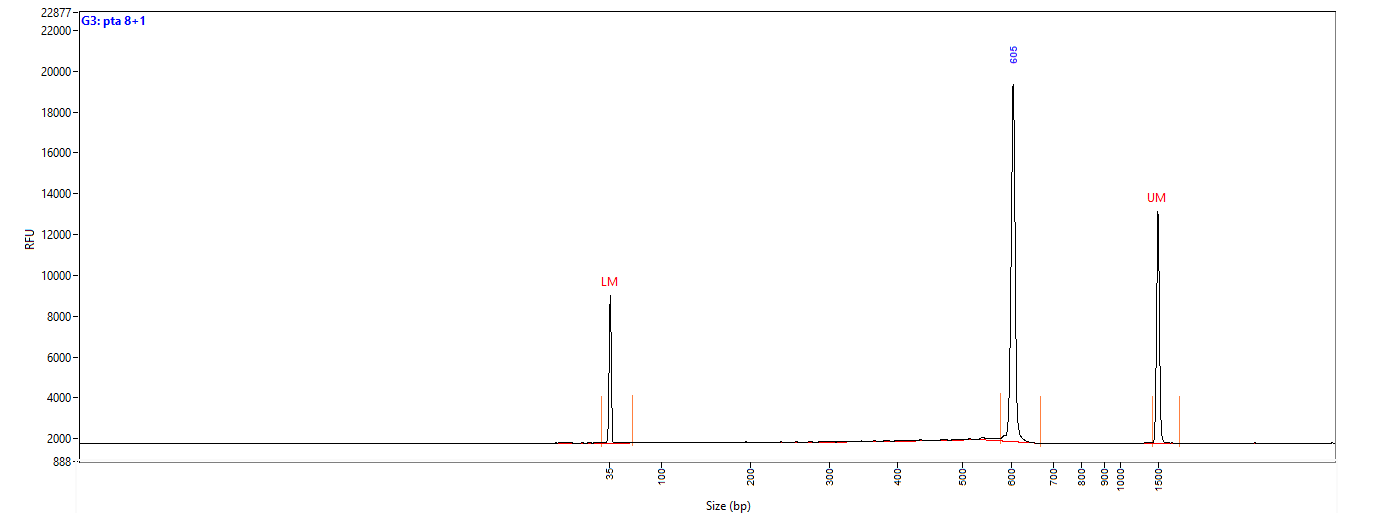


BCER8 (allele 4) vs BCER4 (allele 5): 1 SNP 170, 433, 605 bp


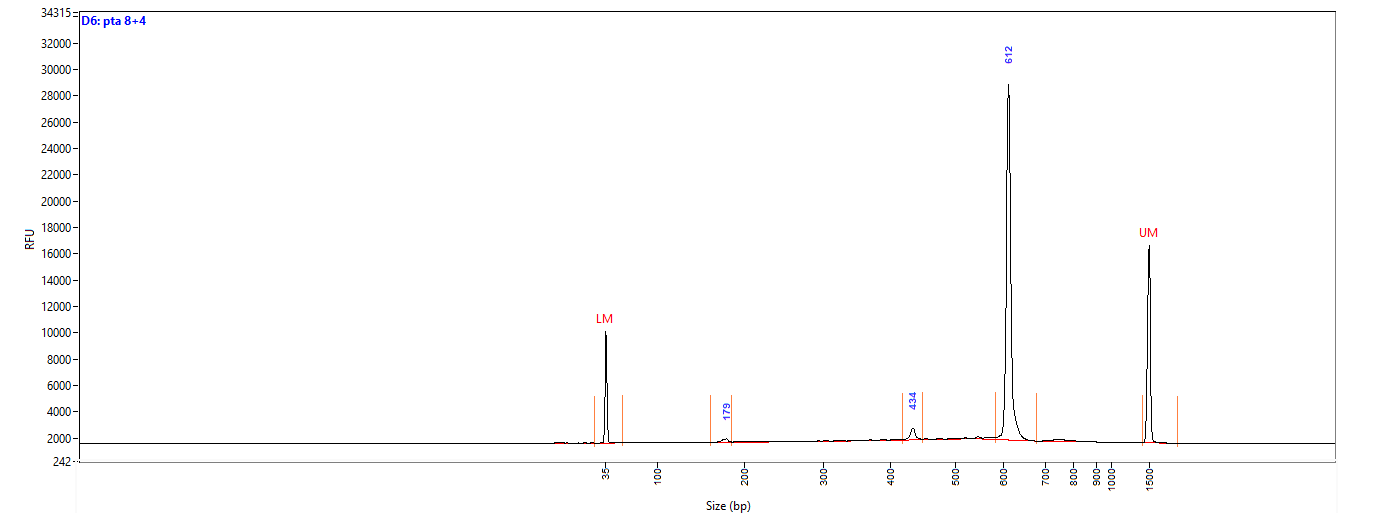


BCER8 (allele 4) vs BCER5 (allele 5): 1 SNP 170, 433, 605 bp


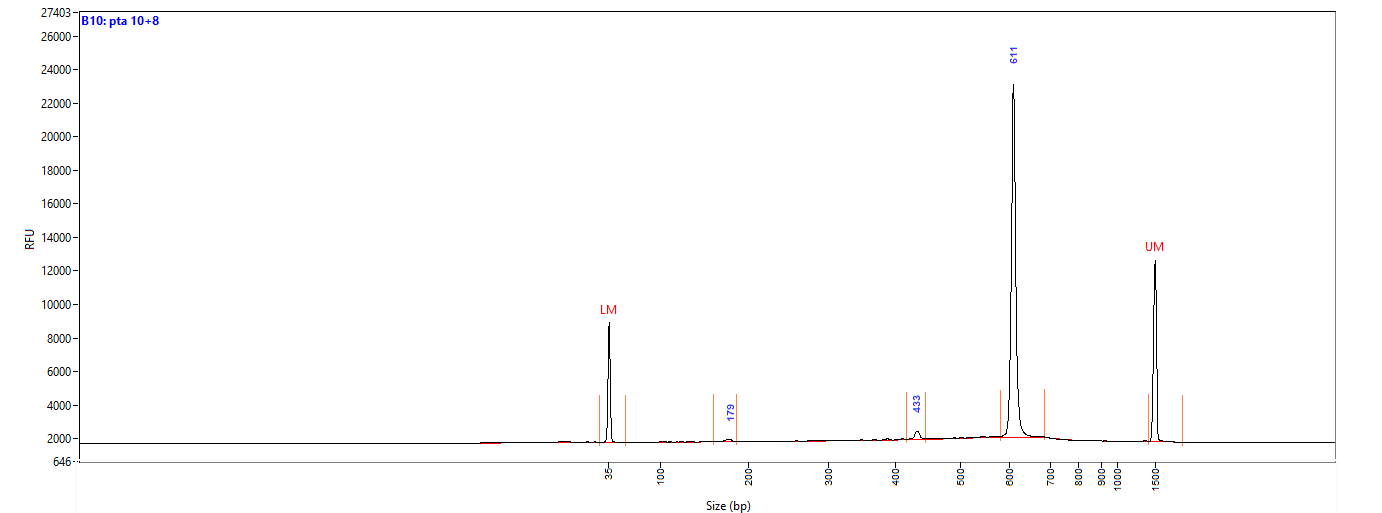


BCER8 (allele 4) vs BCER10 (allele 5): 1 SNP 170, 433, 605 bp


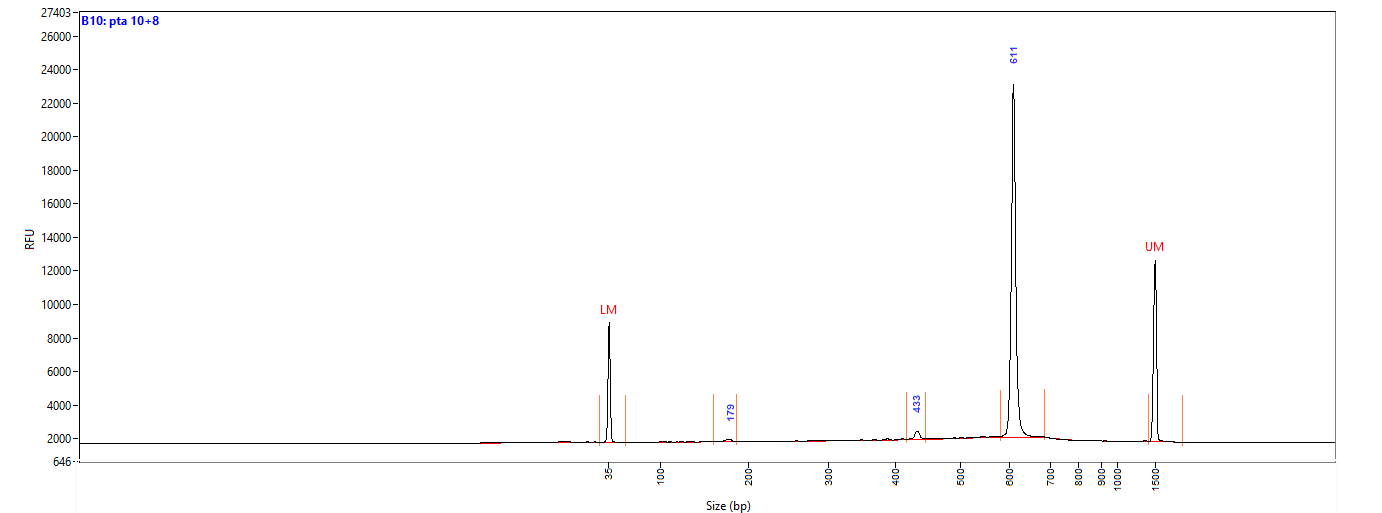


BCER8 (allele 4) vs BCER12 (allele 5): 1 SNP 170, 433, 605 bp


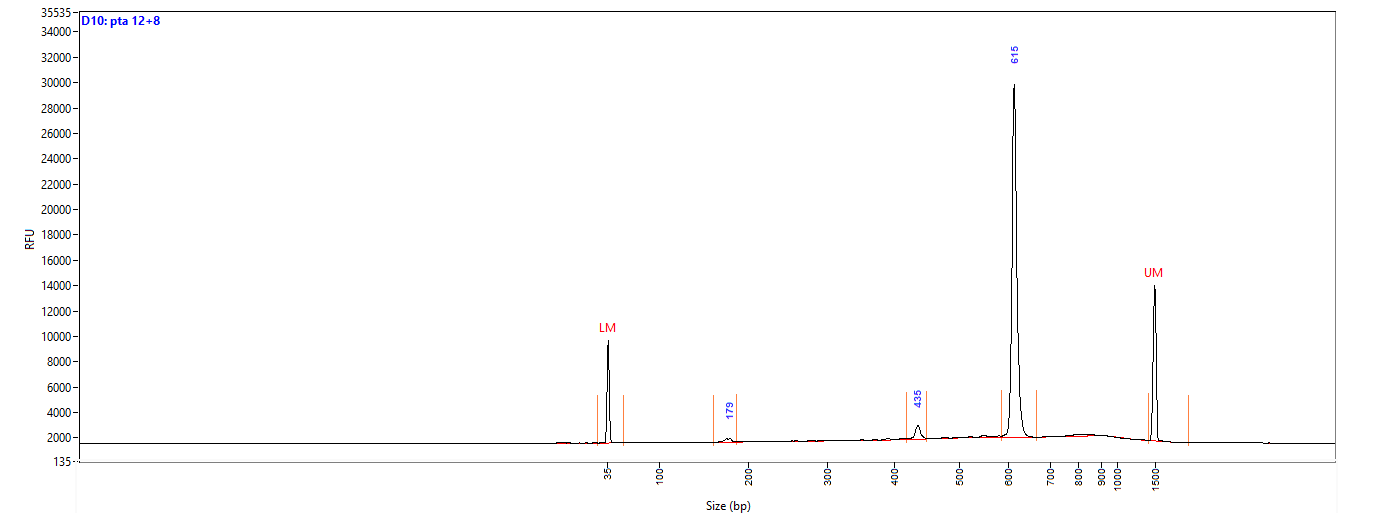


BCER8 (allele 4) vs BCER13 (allele 5): 1 SNP 170, 433, 605 bp


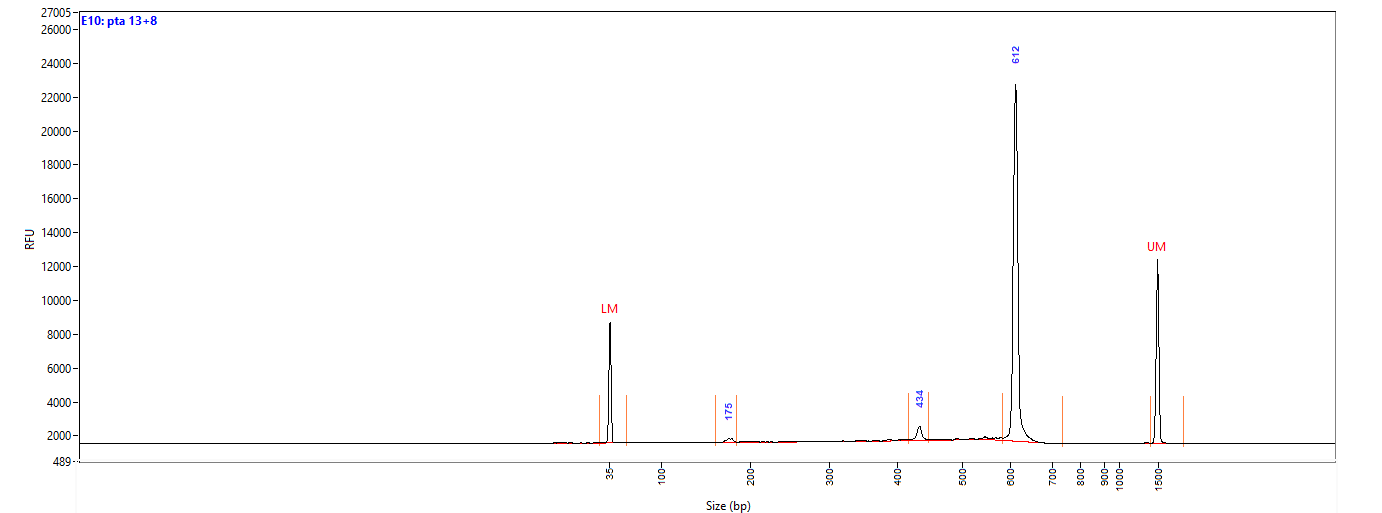


BCER4 (allele 5) vs BCER5 (allele 5): 0 SNP


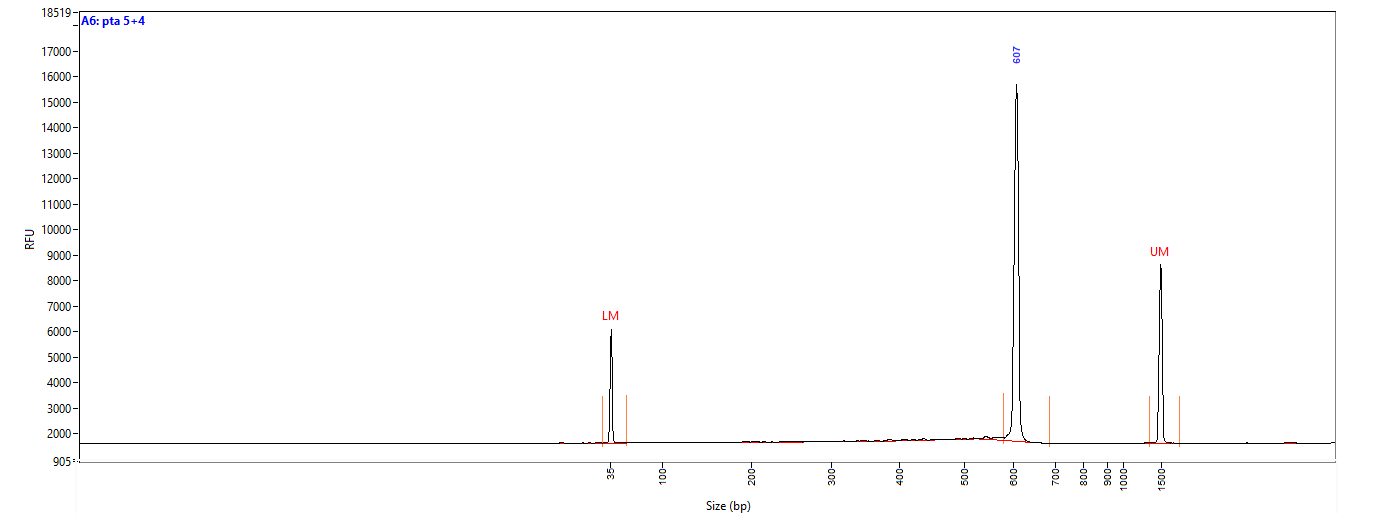


BCER4(allele 5) vs BCER10 (allele 5): 0 SNP


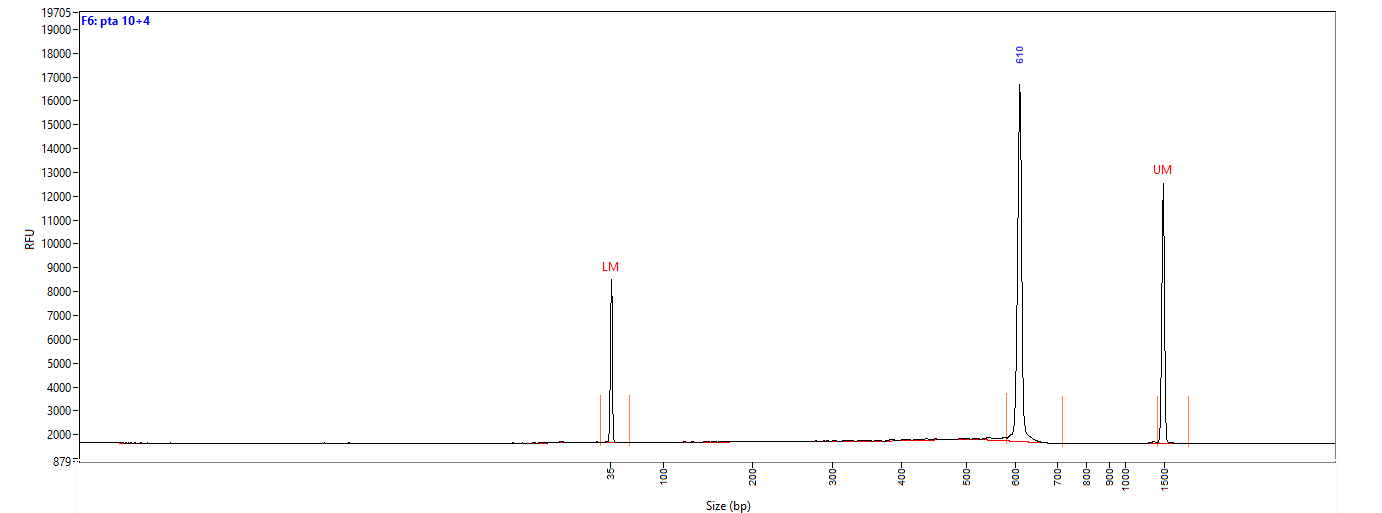


BCER4 (allele 5) vs BCER12 (allele 5): 0 SNP


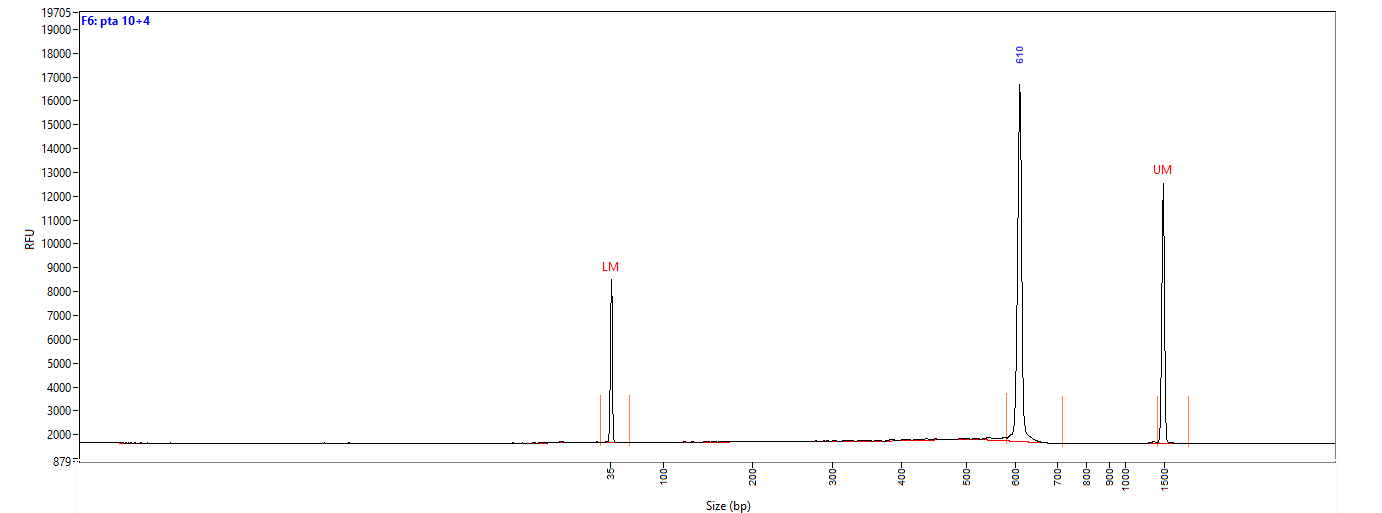


BCER6 (allele 11) vs BCER3 (allele 14): 2 SNP excpected peaks 110, 120,378,500,560, 605 bp


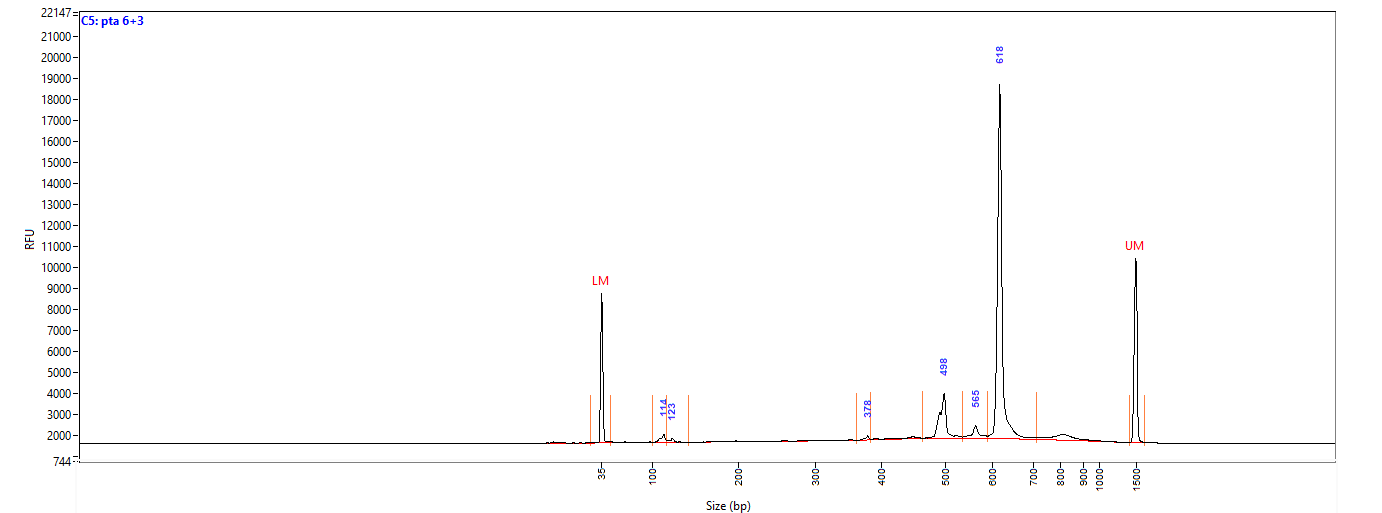


BCER6 (allele 11) vs BCER9 (allele 14): 2 SNP excpected peaks 110, 120,378,500,560, 605 bp


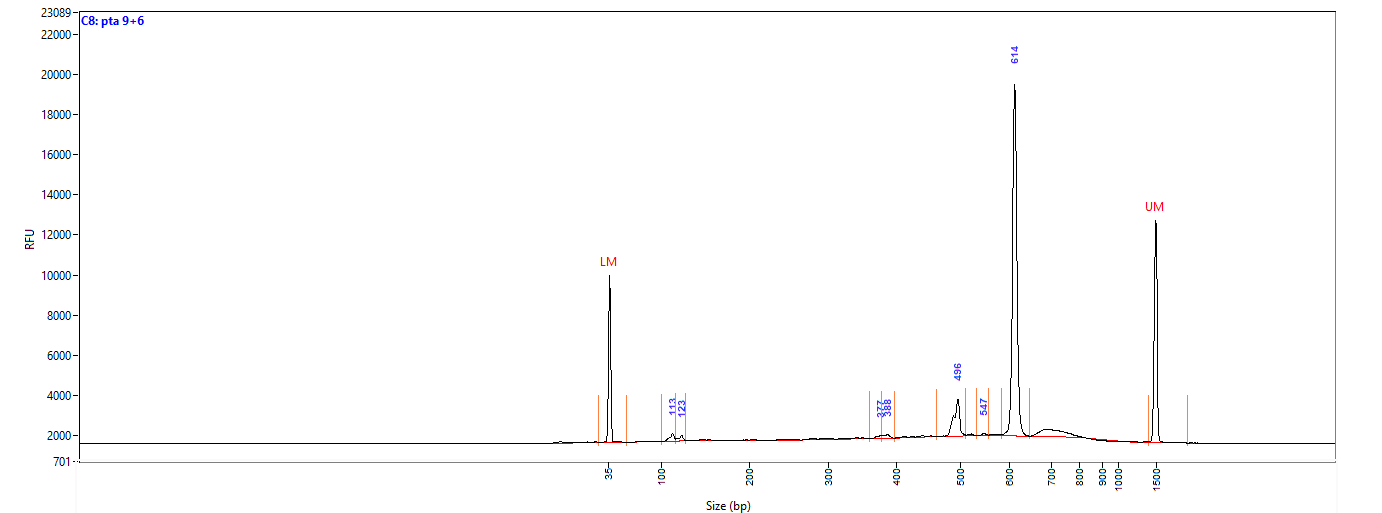


BCER3 (allele 14) vs BCER11 (allele 12): 3 SNP excpected peaks 50, 110, 150, 220, 275, 320, 430, 480, 485, 540, 605 bp


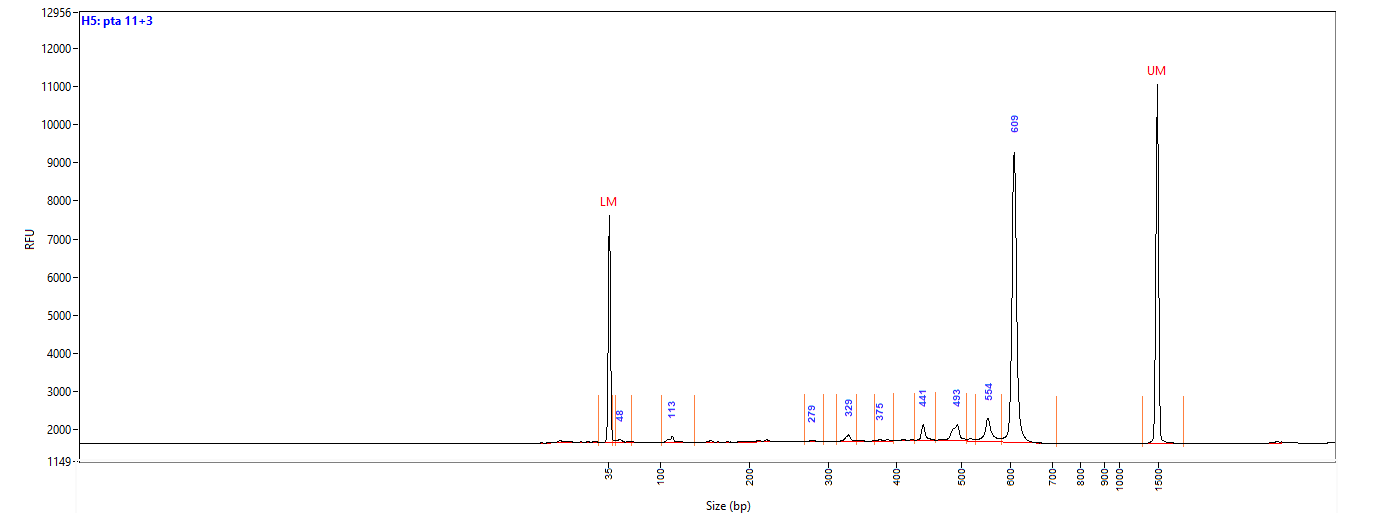


BCER9 (allele 14) vs BCER11 (allele 12): 3 SNP expected peaks 50, 110, 275, 320, 390, 430, 480, 485, 540, 605 bp


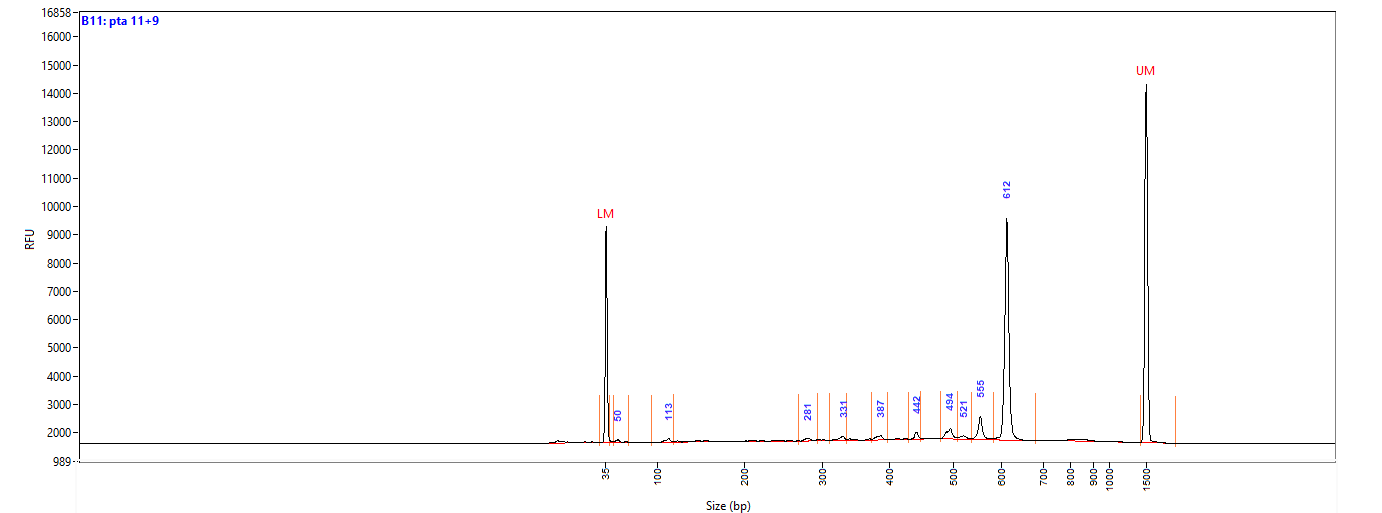


Gene: *pur*

BCER9 (allele 9) vs BCER3 (allele 11): 1 SNP 243, 293, 536 bp


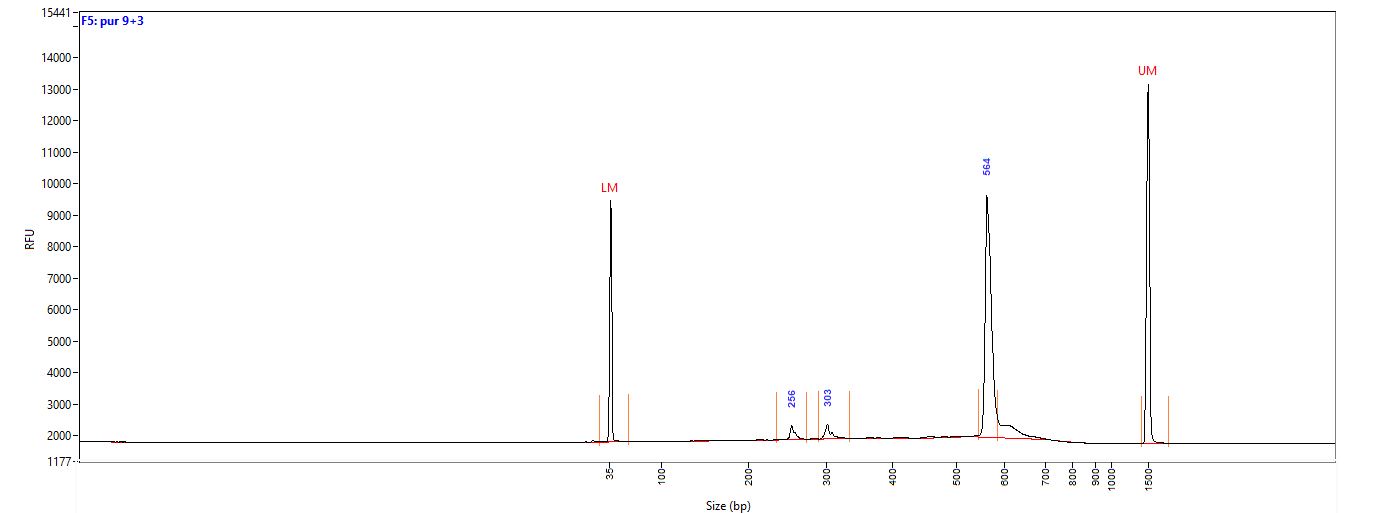


BCER9 (allele 9) vs BCER6 (allele 11): 1 SNP 243, 293, 536 bp


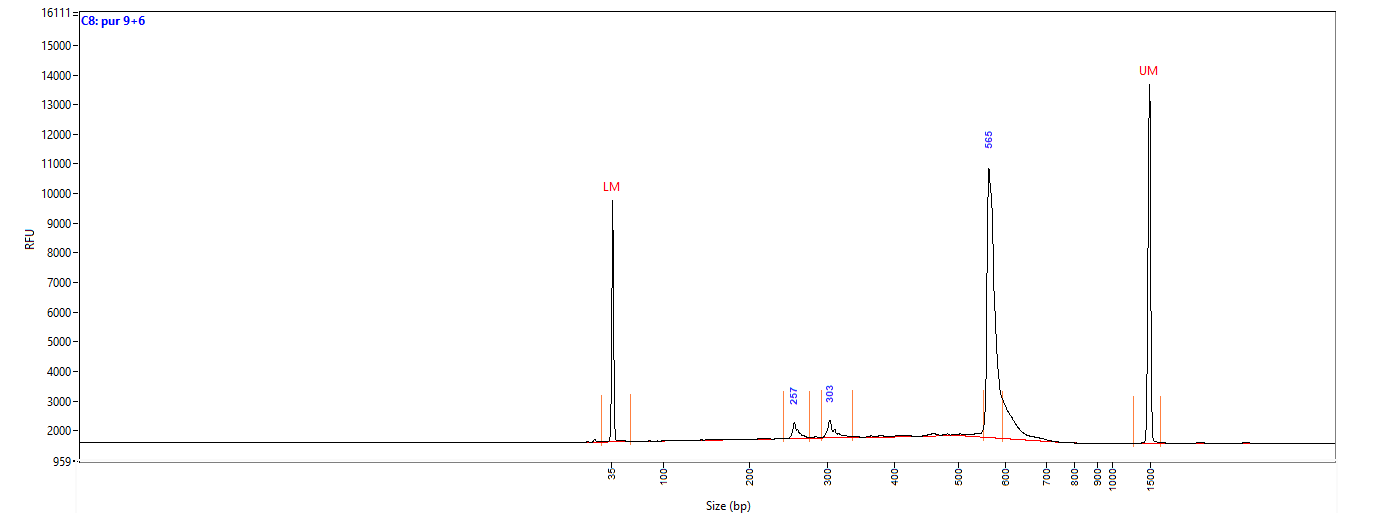


BCER6 (allele 11) vs BCER3 (allele 11): 0 SNP


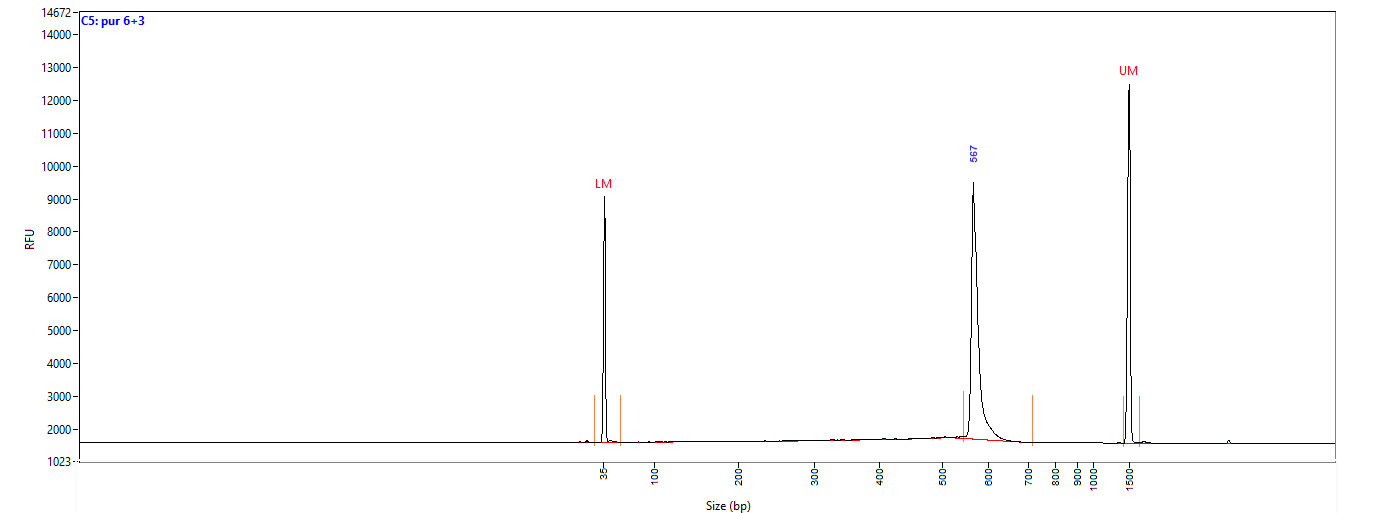


Gene: *pycA*

BCER3 (allele 12) vs BCER7 (allele 16)


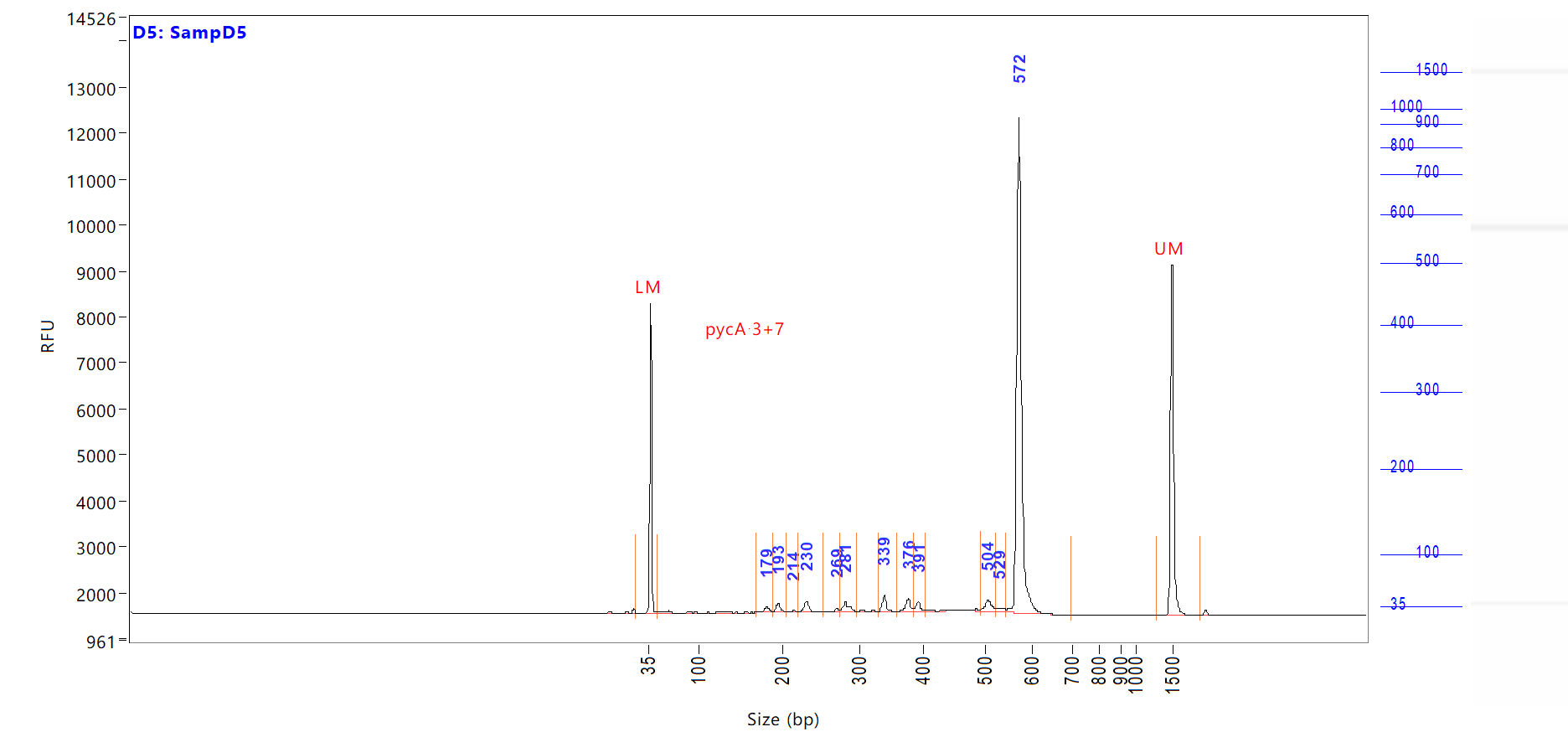


BCER6 (allele 12) vs BCER7 (allele 16)


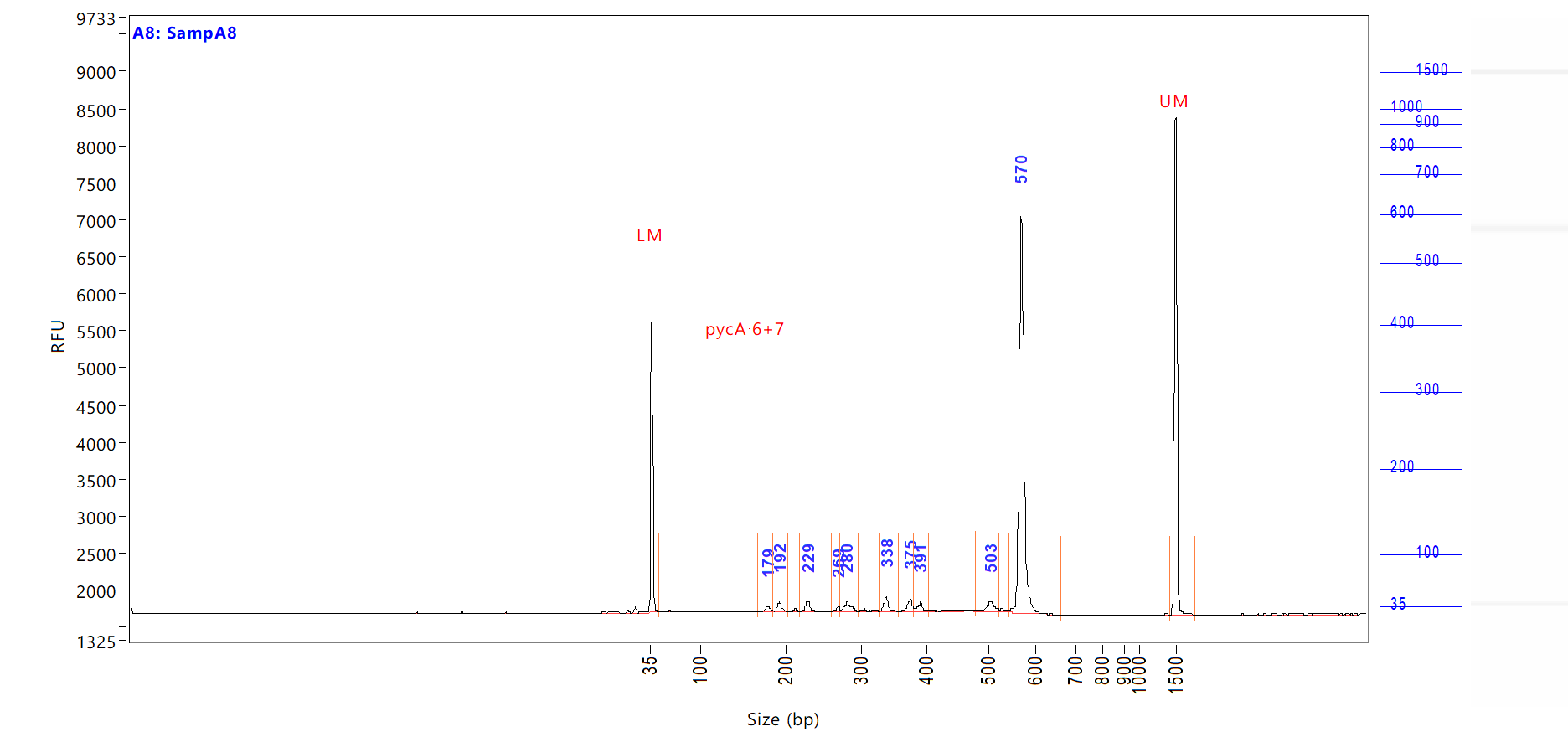


BCER9 (allele 12) vs BCER7 (allele 16)


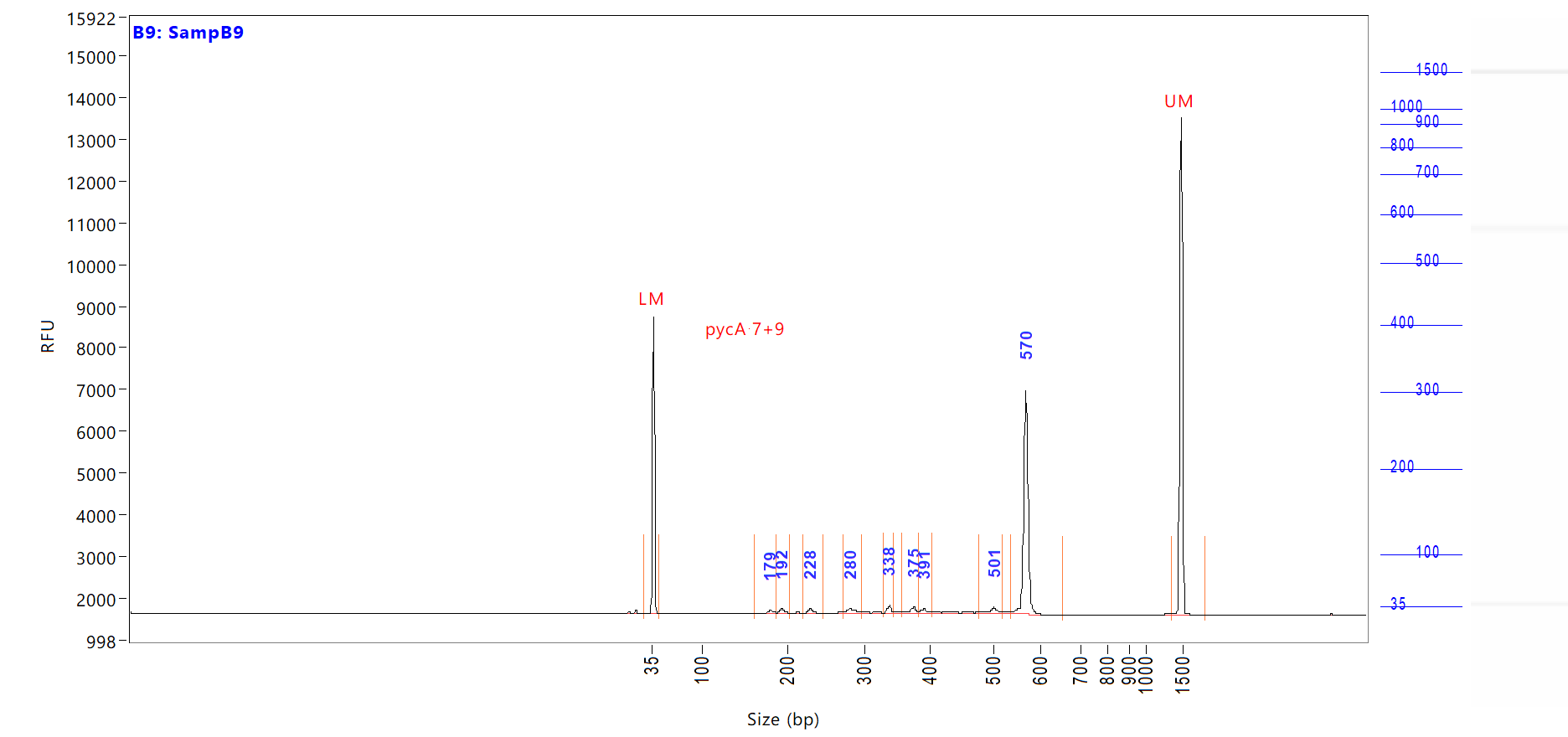


BCER3 (allele 12) vs BCER6 (allele 12): 0 SNP


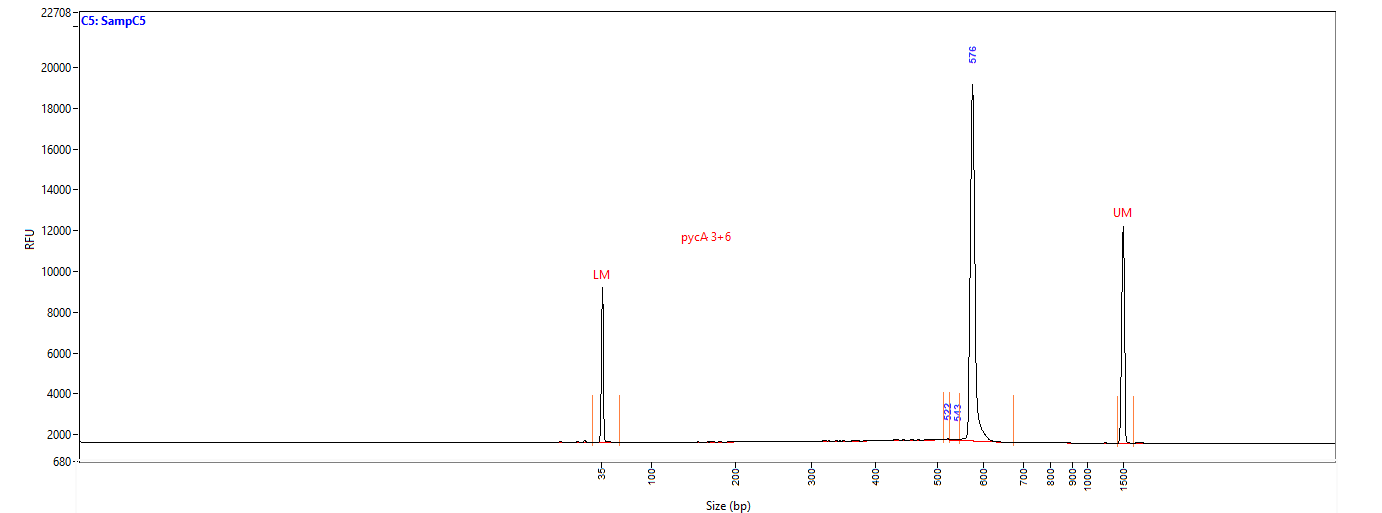


BCER3 (allele 12) vs BCER9 (allele 12): 0 SNP


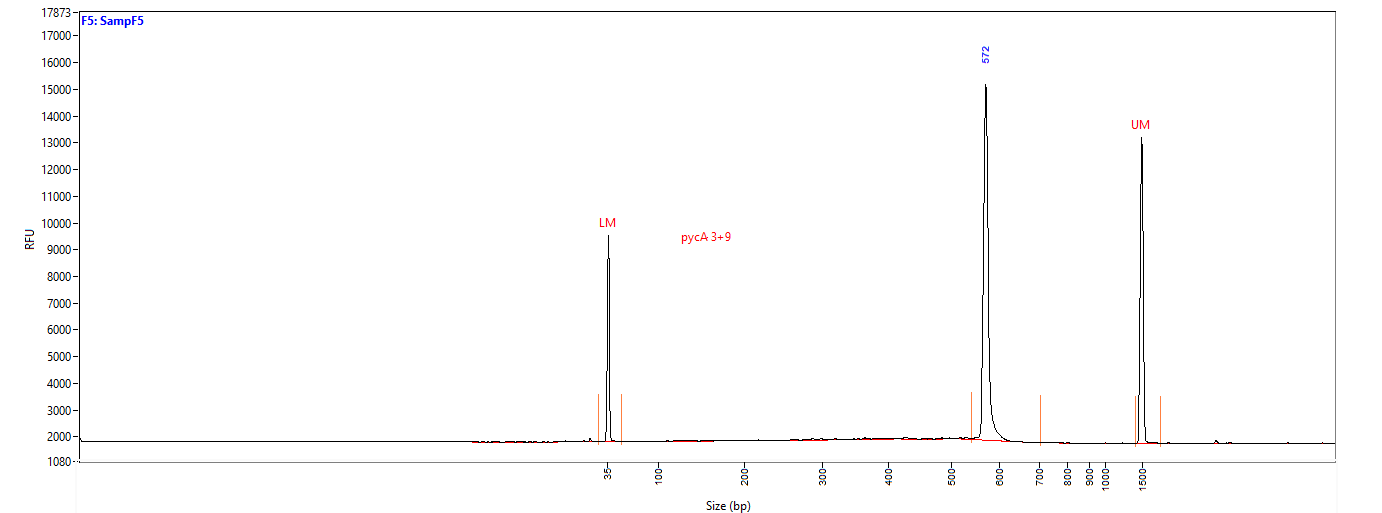


BCER6 (allele 12) vs BCER9 (allele 12): 0 SNP


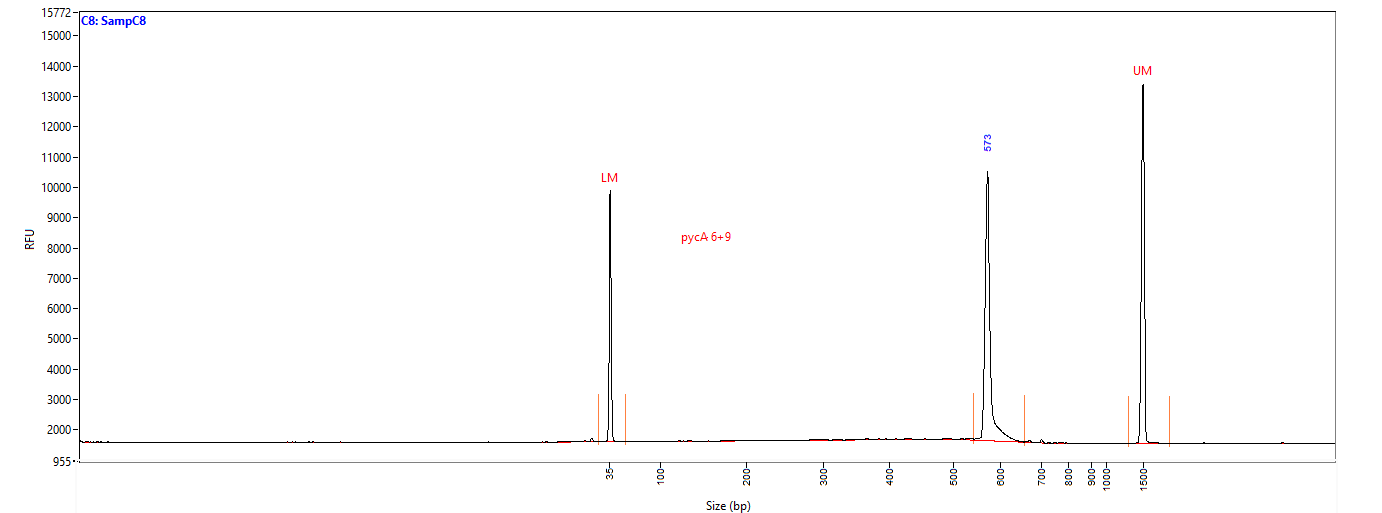


**SUPPLEMENTARY DATA D: Table describing isolate information and allelic profiles for *S. haemolyticus* shortlisted isolates, followed by an example of electropherograms for locus *SH_1431***

| **Isolates** | **Group** | **Origin** | **Neonatal center** | **ST** | **Allele combination** | | | | | | |
| --- | --- | --- | --- | --- | --- | --- | --- | --- | --- | --- | --- |
|  | | | | | ***arcC*** | ***SH_1200*** | ***hemH*** | ***leuB*** | ***SH_1431*** | ***cfxE*** | ***ribABC*** |
| SH1 | Outbreak/inv1 | Blood culture | NC-1 | ST29 | *1* | *1* | *1* | *1* | *2* | *1* | *4* |
| SH2 | Outbreak/inv1 | Blood culture | NC-1 | ST29 | *1* | *1* | *1* | *1* | *2* | *1* | *4* |
| SH14 | Outbreak/inv1 | Blood culture | NC-1 | ST49 | *1* | *5* | *1* | *1* | *1* | *1* | *4* |
| SH29 | Outbreak/inv2 | Blood culture | NC-2 | ST30 | *2* | *5* | *1* | *1* | *1* | *1* | *4* |
| SH30 | Outbreak/inv2 | Blood culture | NC-1 | ST29 | *1* | *1* | *1* | *1* | *2* | *1* | *4* |
| SH38 | Control | Blood culture | NT | ST52 | *2* | *1* | *6* | *1* | *2* | *1* | *4* |
| SH39 | Control | Blood culture | NT | ST25 | *2* | *1* | *1* | *1* | *5* | *1* | *4* |
| SH40 | Control | Blood culture | NT | ST29 | *1* | *1* | *1* | *1* | *2* | *1* | *4* |


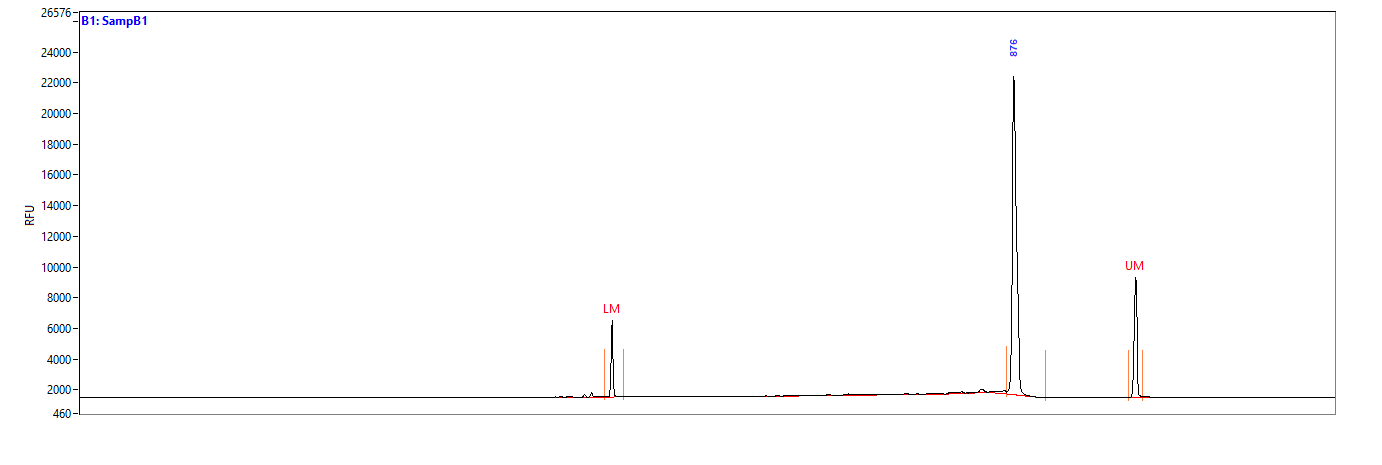
SHAE locus *SH_1431*: (ST29) Allele 2 vs (ST29) Allele 2

\

SHAE locus *SH_1431*: (ST29) Allele 2 vs (ST52) Allele 2


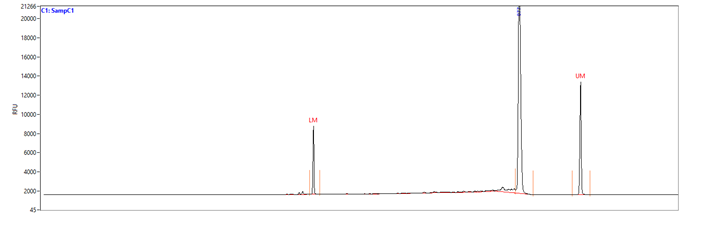


SHAE locus *SH_1431*: (ST29) Allele 2 vs (ST30) Allele 1


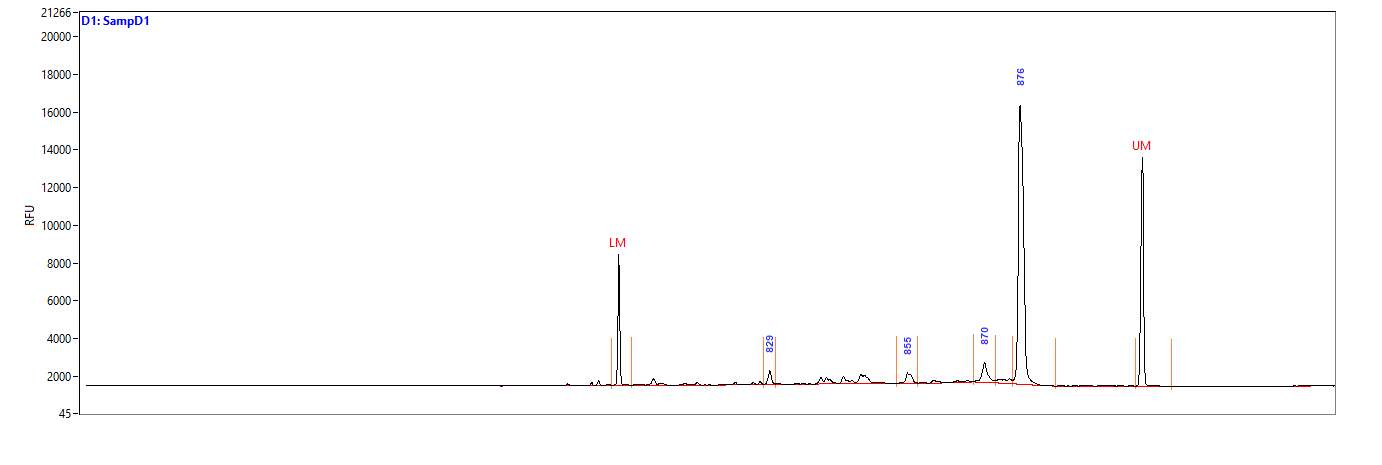


SHAE locus *SH_1431*: (ST29) Allele 2 vs (ST49) Allele 1


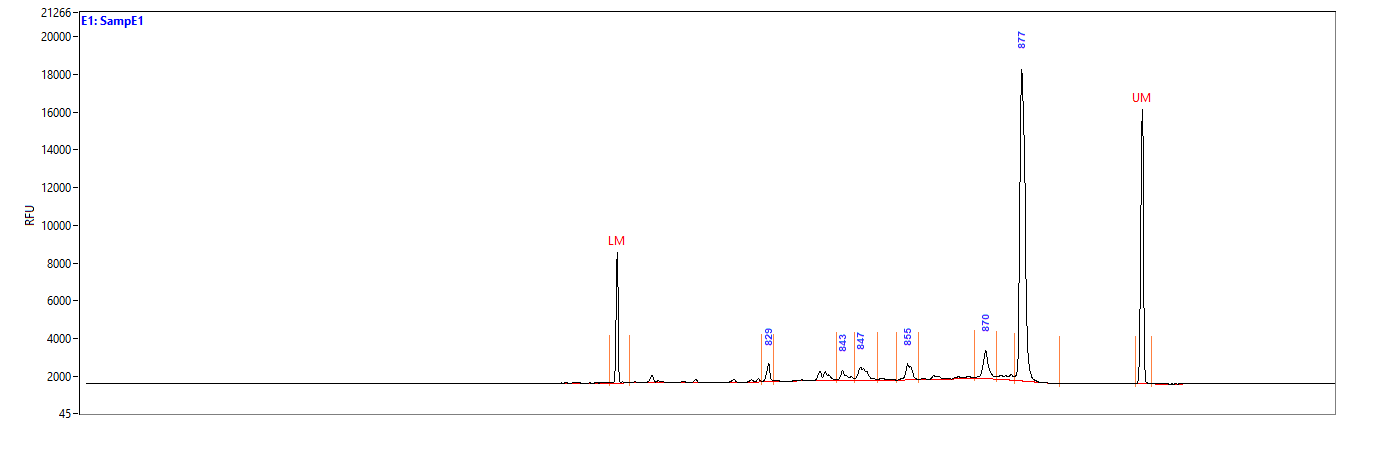


SHAE locus *SH_1431*: (ST29) Allele 2 vs (ST25) Allele 5


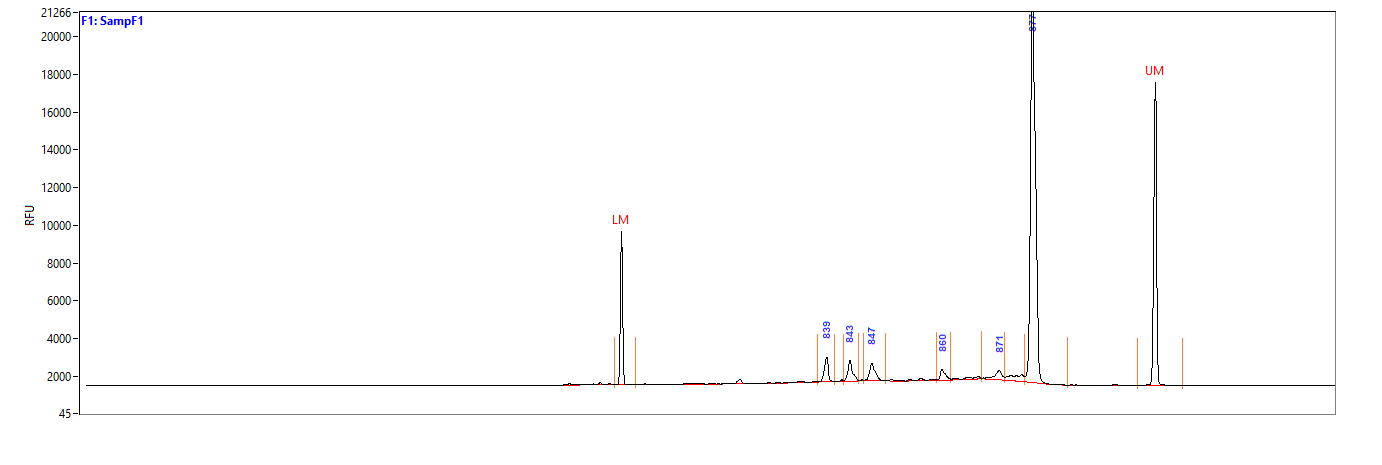


SHAE locus *SH_1431*: (ST52) Allele 2 vs (ST30) Allele 1


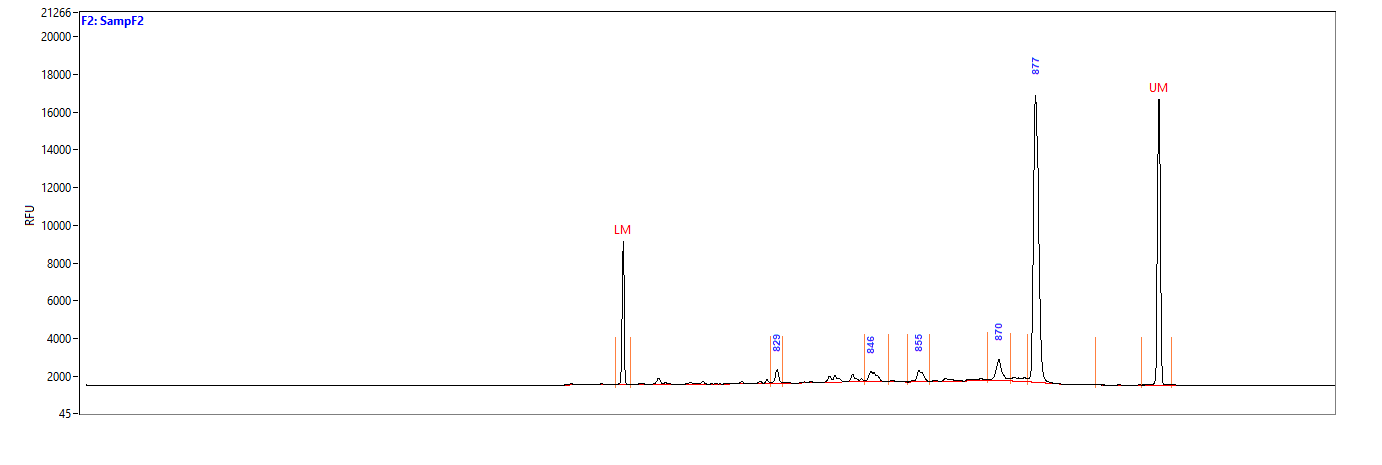


SHAE locus *SH_1431*: (ST52) Allele 2 vs (ST25) Allele 5


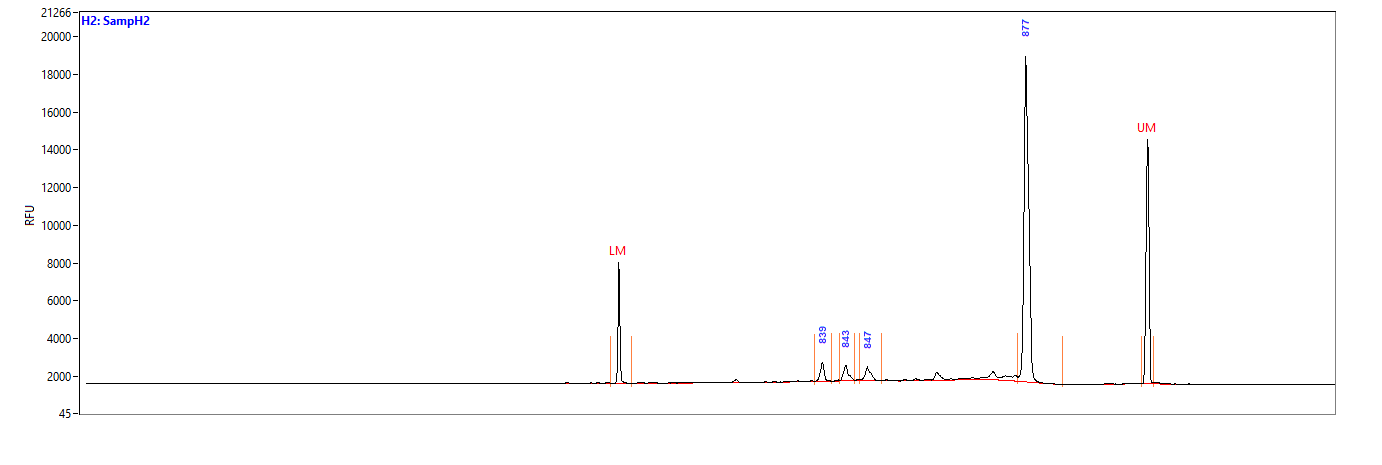


SHAE locus *SH_1431*: (ST52) Allele 2 vs (ST49) Allele 1


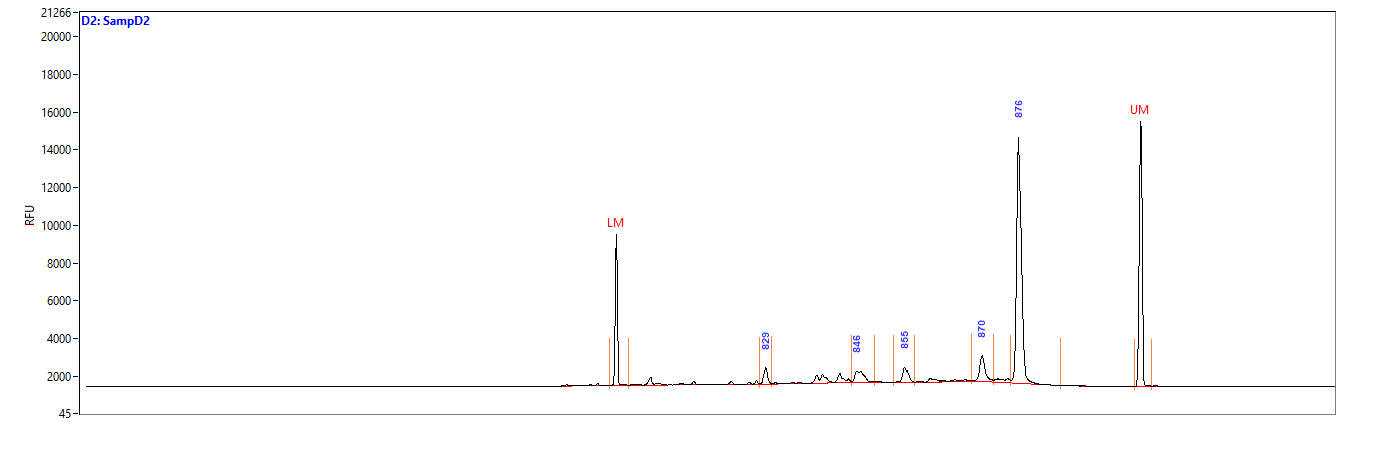


SHAE locus *SH_1431*: (ST30) Allele 1 vs (ST49) Allele 1


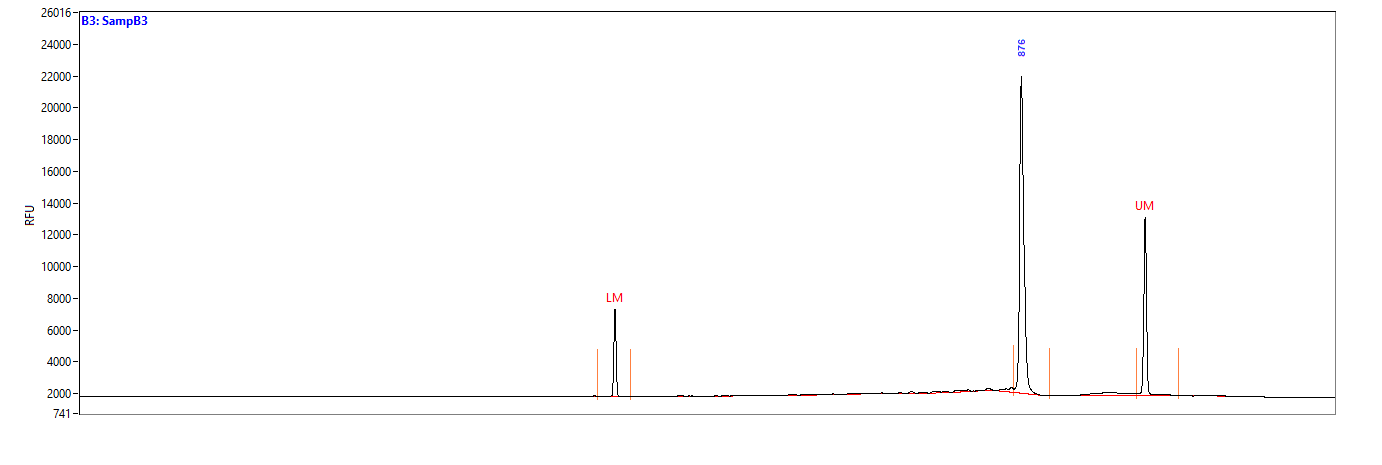


SHAE locus *SH_1431*: (ST30) Allele 1 vs (ST25) Allele 5


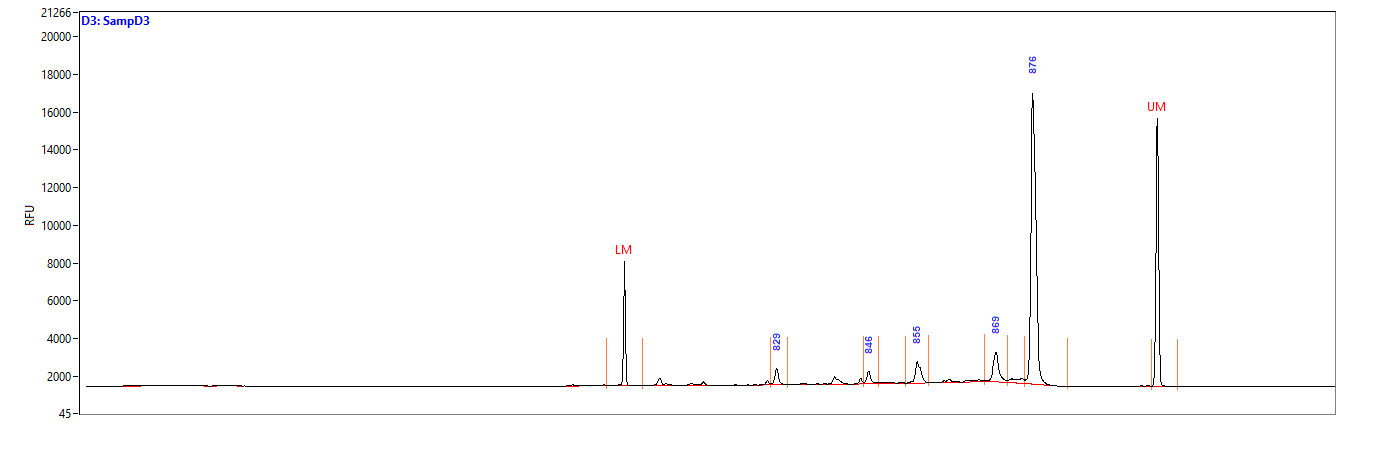


SHAE locus *SH_1431*: (ST49) Allele 1 vs (ST25) Allele 5


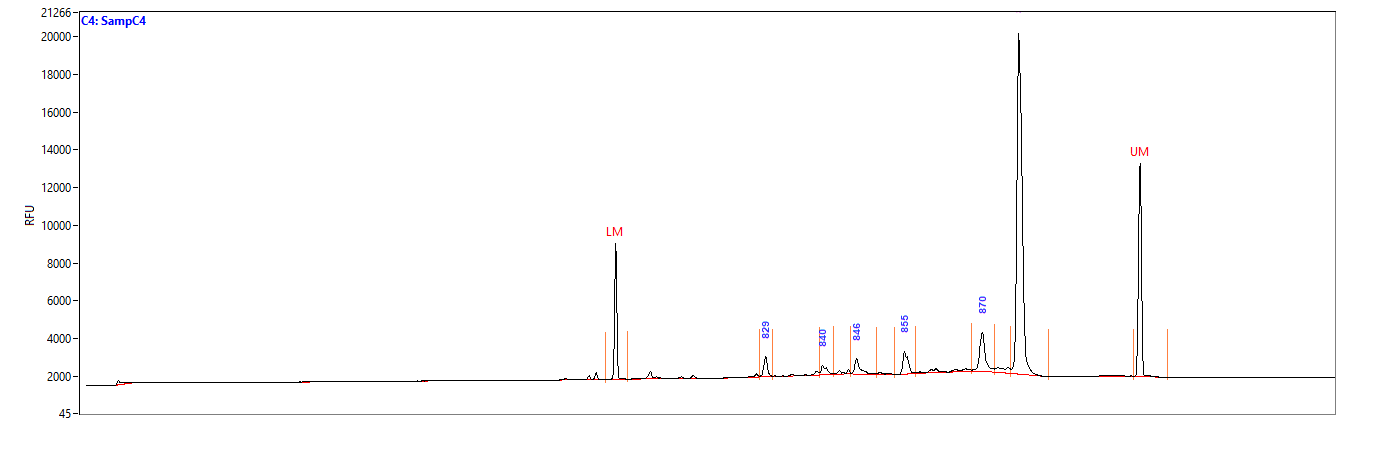

Supplement: Supplementary file 1 [file DataSheet1.docx]
